# Comparative effects of hypnotic agents on sleep architecture and respiratory outcomes in obstructive sleep apnea: A systematic review and network meta‐analysis

**DOI:** 10.1111/pcn.70036

**Published:** 2026-02-10

**Authors:** Taro Kishi, Toshikazu Ikuta, Kenji Sakuma, Masakazu Hatano, Tatsuhiko Kishi, Tsuyoshi Kitajima, Nakao Iwata

**Affiliations:** ^1^ Department of Psychiatry Fujita Health University School of Medicine Toyoake Japan; ^2^ Department of Communication Sciences and Disorders, School of Applied Sciences University of Mississippi University Mississippi USA; ^3^ Department of Pharmacotherapeutics and Informatics Fujita Health University School of Medicine Toyoake Japan; ^4^ Kishi Ear Nose Throat Clinic Ichinomiya Japan

**Keywords:** hypnotics, obstructive sleep apnea, respiratory outcome, sleep architecture, systematic review and network meta‐analysis

## Abstract

**Aim:**

This network meta‐analysis of randomized controlled trials (RCTs) aimed to investigate which hypnotics are associated with the most favorable sleep architecture and respiratory outcomes in adults with obstructive sleep apnea.

**Methods:**

Primary outcomes included total sleep time (TST) and apnea–hypopnea index (AHI) during TST. Other outcomes were rapid eye movement (REM) sleep time, latency to persistent sleep (LPS), wake after sleep onset (WASO), sleep efficiency (SE), AHI during non‐REM or REM sleep, mean peripheral oxygen saturation (SpO_2_) during TST, mean SpO_2_ nadir during TST, arousal index (AI), all‐cause discontinuation, adverse event‐related discontinuation, and incidence of individual adverse events. Effect sizes with 95% confidence intervals were calculated.

**Results:**

This systematic review included 32 RCTs (*n* = 1871, average age = 51.60 years, 62.52% male, mean AHI = 23.60). Our network meta‐analysis evaluated brotizolam, daridorexant, eszopiclone, flurazepam, lemborexant, nitrazepam, ramelteon, temazepam, triazolam, zaleplon, zolpidem, zopiclone, and placebo. Compared with placebo, lemborexant increased TST, REM sleep time, and SE and decreased LPS and WASO, whereas both daridorexant and zolpidem increased TST and SE and decreased WASO. These three medications demonstrated respiratory safety and discontinuation profiles similar to those of placebo. Eszopiclone increased TST and SE and decreased LPS, WASO, AHI during TST, and AI, but its effects on LPS, WASO, AHI during TST, and AI disappeared in the sensitivity analysis, excluding continuous positive airway pressure titration studies.

**Conclusion:**

Our network meta‐analysis identified different effects of various hypnotics on sleep architecture and respiratory parameters; however, the lack of data prevented a formal synthesis of subjective outcomes. Therefore, these results should be interpreted with caution in clinical practice.

Obstructive sleep apnea (OSA) is a sleep disorder characterized by recurrent episodes of complete (apnea) or partial (hypopnea) upper airway collapse, causing oxygen desaturation or arousal from sleep.^1^, ^2^, ^3^ This condition affects approximately 25% of adults in the United States.^4^, ^5^ OSA is the leading cause of excessive sleepiness, reduced quality of life, and impaired work performance. Further, it increases the risk of hypertension, diabetes, heart disease, and stroke.^3^, ^6^

Up to 39%–58% of individuals with OSA experience insomnia symptoms, and between 29% and 67% of individuals with insomnia disorder have an apnea–hypopnea index (AHI, events/h sleep) of ≥5.^7^, ^8^ Comorbid insomnia and sleep apnea (COMISA) is associated with a significantly greater symptom burden compared with either condition alone. COMISA represents a complex clinical challenge characterized by highly fragmented sleep and a state of persistent physiological hyperarousal.^9^ Compared with individuals with OSA alone, patients with COMISA demonstrate longer sleep onset latency, poorer overall sleep quality, and marked reductions in restorative slow‐wave sleep and rapid eye movement (REM) sleep. The pathophysiology of COMISA is induced by a lowered arousal threshold, triggering exaggerated sympathetic nervous system responses to apneic events. This hyperarousal frequently shifts the daytime symptom profile from classic excessive daytime sleepiness to persistent mental exhaustion, cognitive impairment, and increased anxiety and depression levels.

Recent clinical practical guidelines (CPGs) recommend against the initial use of hypnotics to treat COMISA, instead prioritizing OSA treatment.^10^, ^11^ Oral appliance (OA) treatment is recommended for patients with mild to moderate OSA who cannot use or are not indicated for continuous positive airway pressure (CPAP) treatment.^3^, ^10^, ^11^, ^12^ CPAP is recommended for moderate to severe OSA.^3^, ^10^, ^11^, ^12^ However, comorbid insomnia complicates CPAP treatment, as patients with chronic insomnia are more sensitive to bedtime disruptions,^13^ causing poor CPAP adherence.^14^, ^15^

Other CPGs recommend cognitive behavioral therapy for insomnia symptoms in individuals with OSA.^16^, ^17^, ^18^ However, pharmacotherapy is sometimes used and may be preferred by patients in clinical practice.^19^, ^20^ The major categories of hypnotics approved by the US Food and Drug Administration (FDA) for primary insomnia treatment include benzodiazepines (BENZs), non‐BENZs (also called ‘Z‐drugs’), melatonin receptor agonists, and, most recently, dual orexin receptor antagonists (DORAs).^21^ BENZs and Z‐drugs act on γ‐aminobutyric acid type A receptors by modulating inhibitory neurotransmission in the brain to produce hypnotic, anxiolytic, muscle‐relaxing, and anticonvulsant effects.^22^ The CPGs indicate that potential adverse effects of these medications include the aggravation of underlying OSA, including a decrease in respiratory events due to reduced upper airway hypotonia and ventilatory response, along with prolonged respiratory event time.^10^ However, some studies revealed that ramelteon, daridorexant, and lemborexant did not worsen AHI in individuals with OSA compared with placebo. Thus, the effects on respiratory parameters in OSA might be class‐specific, with distinct differences observed across pharmacological categories (Table S1).

This systematic review and network meta‐analysis aimed to identify the hypnotics providing optimal sleep architecture and respiratory safety in adults with OSA. Using a network meta‐analysis framework, which synthesizes direct and indirect evidence to estimate relative treatment effects,^23^ we assessed 17 distinct outcomes categorized into sleep architecture, respiratory function, treatment acceptability, and treatment tolerability.

## Methods

This study adhered to the Preferred Reporting Items for Systematic Reviews and Meta‐Analyses guidelines^24^ (Table S2) and the Cochrane Handbook for Systematic Reviews of Interventions^23^ and was registered on the Open Science Framework (https://osf.io/rpv8m). At least two of the authors verified the literature search, data transfer accuracy, and calculations. This was a systematic review and meta‐analysis of previously published data; thus, institutional review board approval and patient consent were not required.

### Search strategy and inclusion criteria

We conducted a systematic review using the ‘PICO’ framework, including randomized controlled trials (RCTs) of hypnotics *versus* placebo or other active treatments in adults with OSA, with outcomes focusing on sleep architecture, respiratory function, treatment acceptability, and treatment tolerability. Inclusion criteria were (i) studies including individuals with any severity of OSA; and (ii) studies involving individuals with or without concurrent OA or CPAP treatment. Participants were not required to have a formal insomnia diagnosis based on standardized criteria, including the Diagnostic and Statistical Manual of Mental Disorders^25^ at baseline. Our systematic review included only hypnotics categorized into the major categories by the US FDA for primary insomnia treatment (i.e., BENZs, Z‐drugs, melatonin receptor agonists, and DORAs).^21^ Exclusion criteria were (i) studies only including arms with doses not recommended in the United States,^26^ Europe,^27^ or Japan^28^; and (ii) studies investigating combination therapy of hypnotics with other agents, including atomoxetine. We searched PubMed, the Cochrane Library, and Embase databases for studies published before December 1, 2025, without language restriction (Fig. S1).

### Outcome measures, data synthesis, and data extraction

Primary outcomes include total sleep time (TST) and the AHI during TST. These polysomnography‐derived parameters were selected as they represented the prespecified primary endpoints in most studies included in this systematic review. Other outcomes associated with sleep architecture included REM sleep time, latency to persistent sleep (LPS), wake after sleep onset (WASO), and sleep efficiency (SE). Other safety outcomes associated with respiratory function were AHI during non‐REM and REM sleep, mean peripheral oxygen saturation (SpO_2_) during TST, mean SpO_2_ nadir during TST, arousal index (AI), and CPAP use per night on all nights (for network meta‐analysis, including only CPAP titration studies). Further, all‐cause discontinuation, adverse event‐related discontinuation, and incidence of individual adverse events were included. We analyzed data utilizing intention‐to‐treat or modified intention‐to‐treat principles, and supplemented missing data with data from published systematic reviews.^29^, ^30^

### Meta‐analysis methods

We employed a frequentist network meta‐analysis with a random‐effects model.^31^, ^32^ Standardized mean differences (SMDs) were calculated for continuous variables, whereas odds ratios were calculated for dichotomous variables, with 95% confidence intervals (CIs). Network heterogeneity was assessed utilizing *𝜏* ^2^ statistics. To evaluate coherence, we applied the design‐by‐treatment test globally and the Separating Indirect from Direct Evidence test locally.^33^, ^34^ Treatments were ranked based on the surface under the cumulative ranking curve (SUCRA) values for each outcome. We examined the sufficiency of the distribution differences by comparing the distribution of potential effect modifiers across treatments using the Kruskal–Wallis test (continuous variables) and the Pearson Chi‐square test or Fisher's exact test (categorical variables) and by evaluating their actual influence on the treatment effect through network meta‐regression analyses.^35^, ^36^, ^37^ Potential confounding factors included the proportion of males, mean age, mean AHI, mean body mass index, CPAP usage, study duration, sponsorship, study design (including crossover trials and washout period), total participants, publication year, and overall risk of bias (Table S3). The Cochrane risk of bias tool for randomized trials (version 2) was used to assess the overall risk of bias for each trial (https://www.riskofbias.info/). Further, a sensitivity analysis was performed, excluding CPAP titration studies for polysomnography‐measured outcomes. Moreover, we conducted subgroup analyses: one including studies that involved only non‐CPAP users for polysomnography‐measured outcomes, and another including only CPAP titration for polysomnography‐measured outcomes and CPAP adherence. In this network meta‐analysis, one study on ramelteon that utilized auto‐titrating positive airway pressure was categorized within the CPAP study group.^38^ Finally, we conducted a network meta‐analysis classifying medications by drug class (BENZs, DORAs, ramelteon, or Z‐drugs) for polysomnography‐measured safety outcomes.

The Confidence in Network Meta‐Analysis application, which is an adaptation of the Grading of Recommendations Assessment, Development, and Evaluation approach, was used to evaluate the results to assess the credibility of our findings.^39^, ^40^, ^41^ Publication bias was planned to be assessed using a funnel plot.^23^

## Results

### Study characteristics

Figure S1 details the literature search process. Initially, 236 articles were identified, of which 100 duplicates were removed. After title and abstract screening, 107 articles were excluded, and an additional 5 were excluded after full‐text review. Eight more studies were identified through previous reviews. Finally, the systematic review included 32 articles (reporting 32 RCTs).^38^, ^42^, ^43^, ^44^, ^45^, ^46^, ^47^, ^48^, ^49^, ^50^, ^51^, ^52^, ^53^, ^54^, ^55^, ^56^, ^57^, ^58^, ^59^, ^60^, ^61^, ^62^, ^63^, ^64^, ^65^, ^66^, ^67^, ^68^, ^69^, ^70^, ^71^, ^72^ These studies involved 1871 adults (average age = 51.60 years, 62.52% male, mean AHI = 23.60).

Seven studies were excluded from our network meta‐analysis: two used unrecommended doses,^60^, ^71^ one included both healthy volunteers and patients with OSA,^47^ three investigated combination therapy of hypnotics with other agents,^55^, ^65^, ^69^ and one utilized supplemental oxygen therapy.^57^ Further, the zolpidem 20 mg/day arm from the Cirignotta 1988 study was excluded.^54^ Our network meta‐analysis assessed the following treatment arms: brotizolam, daridorexant, eszopiclone, flurazepam, lemborexant, nitrazepam, ramelteon, temazepam, triazolam, zaleplon, zolpidem, zopiclone, and placebo. Table S1 summarizes the study characteristics. Among the trials included in the network meta‐analysis, 40.74% included only non‐CPAP users, and 48.15% were industry‐sponsored studies. The severity of OSA in the included studies differed. Three studies included only participants with COMISA, diagnosed based on international consensus criteria.^38^, ^46^, ^61^ The overall risk of bias in most studies was rated as ‘some concerns’ or ‘low risk’ (Table S4). Polysomnography‐measured outcomes could not include zaleplon due to a lack of data. No evidence indicated a transitivity assumption violation when comparing study characteristics across different comparisons (Table S3).

### Network meta‐analysis results

#### Outcomes related to sleep architecture

**Fig. 1:**
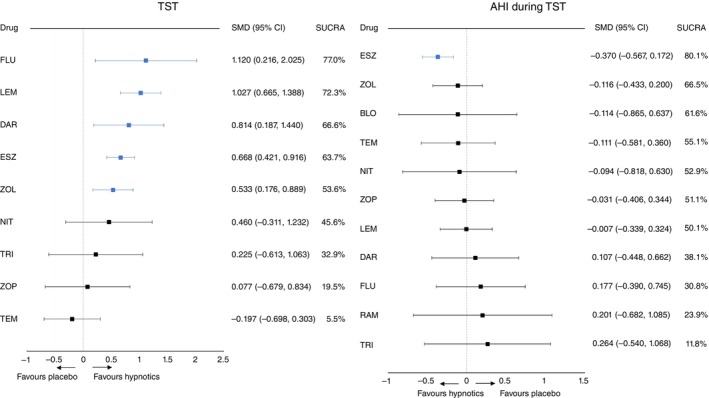
Forest plots. Colors indicate the presence or absence of a statistically significant difference, with blue denoting the superiority of the drug over the placebo and black representing the comparability of the drug to the placebo. 95% CI, 95% confidence interval; AHI, apnea–hypopnea index; BLO, brotizolam; DAR, daridorexant; ESZ, eszopiclone; FLU, flurazepam; LEM, lemborexant; NIT, nitrazepam; RAM, ramelteon; SMD, standardized mean difference; TEM, temazepam; TRI, triazolam; TST, total sleep time; ZOL, zolpidem; ZOP, zopiclone.

Daridorexant, eszopiclone, flurazepam, lemborexant, and zolpidem significantly increased TST compared with placebo (Fig. 1, Table 1, and Appendix S1). The SMDs (95% CI) ranged from 1.120 (0.216, 2.025) for flurazepam to 0.533 (0.176, 0.889) for zolpidem. Further, these medications significantly increased TST compared with temazepam. Moreover, lemborexant significantly increased TST compared with zopiclone.

**Table 1:** Outcomes related to sleep architecture

	TST	REM sleep time	LPS	WASO	SE
Daridorexant	**0.814 (0.187, 1.440)** ^†^	0.247 (−0.309, 0.804)	−0.477 (−1.040, 0.086)	**−0.663 (−1.233, −0.092)** ^†^	**0.813 (0.234, 1.391)** ^†^
Eszopiclone	**0.668 (0.421, 0.916)** ^‡^	0.150 (−0.524, 0.823)	**−0.362 (−0.568, −0.155)** ^§^	**−0.661 (−0.871, −0.450)** ^§^	**0.753 (0.550, 0.956)** ^‡^
Flurazepam	**1.120 (0.216, 2.025)** ^†^		−0.042 (−0.842, 0.759)	**−1.326 (−2.225, −0.426)** ^†^	**1.801 (0.826, 2.777)** ^†^
Lemborexant	**1.027 (0.665, 1.388)** ^‡^	**0.782 (0.539, 1.025)** ^‡^	**−0.560 (−0.792, −0.328)** ^‡^	**−0.779 (−1.045, −0.513)** ^‡^	**1.011 (0.740, 1.282)** ^‡^
Nitrazepam	0.460 (−0.311, 1.232)	−0.661 (−1.405, 0.082)			
Ramelteon			**−0.942 (−1.877, −0.006)** ^†^		0.374 (−0.515, 1.264)
Temazepam	−0.197 (−0.698, 0.303)	−0.511 (−1.142, 0.120)	−0.024 (−0.644, 0.596)	**−0.938 (−1.698, −0.178)** ^†^	0.017 (−0.452, 0.486)
Triazolam	0.225 (−0.613, 1.063)				
Zolpidem	**0.533 (0.176, 0.889)** ^‡^	0.048 (−0.176, 0.272)	0.042 (−0.586, 0.670)	**−0.660 (−1.024, −0.296)** ^‡^	**0.764 (0.112, 1.417)** ^‡^
Zopiclone	0.077 (−0.679, 0.834)	−0.053 (−0.427, 0.322)	0.353 (−0.455, 1.160)	−0.270 (−0.991, 0.451)	0.497 (−0.049, 1.043)

^†^ Statistical significance persisted after excluding CPAP titration studies in the sensitivity analysis.

^‡^ Statistical significance persisted after excluding CPAP titration studies in the sensitivity analysis and the subgroup analysis of studies involving only non‐CPAP users.

^§^ Statistical significance disappeared after excluding CPAP titration studies in the sensitivity analysis and the subgroup analysis of studies involving only non‐CPAP users.

The values in each cell represent the standardized mean differences and their 95% confidence intervals for each hypnotic compared with placebo.

LPS, latency to persistent sleep; REM, rapid eye movement; SE, sleep efficiency; TST, total sleep time; WASO, wake after sleep onset. Boldface values indicate statistically significant results.

Lemborexant significantly increased REM sleep time compared with placebo (Table 1 and Appendix S2). Eszopiclone, lemborexant, and ramelteon significantly reduced LPS compared with placebo (Table 1 and Appendix S3). Daridorexant, eszopiclone, flurazepam, lemborexant, temazepam, and zolpidem significantly decreased WASO compared with placebo (Table 1 and Appendix S4). Daridorexant, eszopiclone, flurazepam, lemborexant, and zolpidem significantly increased SE compared with placebo (Table 1 and Appendix S5).

#### Outcomes related to respiratory function

Eszopiclone significantly decreased AHI during TST compared with placebo (SMDs [95% CI] of −0.370 [−0.567, −0.172], Fig. 1, Table 2, and Appendix S6).

**Table 2:** Outcomes related to respiratory function

	AHI	AHI during non‐REM sleep	AHI during REM sleep	Mean SpO_2_	Mean SpO_2_ nadir	Arousal index
Brotizolam	−0.114 (−0.865, 0.637)			−0.089 (−0.830, 0.653)	−0.065 (−0.814, 0.683)	
Daridorexant	0.107 (−0.448, 0.662)	0.074 (−0.480, 0.629)	0.100 (−0.455, 0.655)	0.181 (−0.374, 0.737)		
Eszopiclone	**−0.370 (−0.567, −0.172)** ^†^	−0.332 (−1.009, 0.346)	−0.172 (−0.845, 0.502)	0.464 (−0.218, 1.147)	−0.137 (−0.479, 0.205)	**−0.420 (−0.754, −0.086)** ^†^
Flurazepam	0.177 (−0.390, 0.745)			−0.304 (−0.832, 0.224)	−0.131 (−0.698, 0.437)	
Lemborexant	−0.007 (−0.339, 0.324)	−0.015 (−0.347, 0.316)	−0.114 (−0.446, 0.217)	0.024 (−0.307, 0.356)		
Nitrazepam	−0.094 (−0.818, 0.630)					
Ramelteon	0.201 (−0.682, 1.085)					
Temazepam	−0.111 (−0.581, 0.360)			**−0.777 (−1.523, −0.031)** ^‡^	0.190 (−0.432, 0.811)	0.158 (−0.463, 0.779)
Triazolam	0.264 (−0.540, 1.068)					
Zolpidem	−0.116 (−0.433, 0.200)	−0.101 (−0.794, 0.593)	−0.083 (−0.448, 0.281)	−0.119 (−0.473, 0.235)	−0.060 (−0.525, 0.406)	−0.245 (−0.876, 0.385)
Zopiclone	−0.031 (−0.406, 0.344)	−0.011 (−0.450, 0.427)	−0.223 (−0.662, 0.217)	−0.226 (−0.603, 0.151)	−0.144 (−0.862, 0.575)	0.034 (−0.341, 0.408)

^†^ Statistical significance disappeared after excluding CPAP titration studies in the sensitivity analysis and the subgroup analysis of studies involving only non‐CPAP users.

^‡^ Statistical significance persisted after excluding CPAP titration studies in the sensitivity analysis.

The values in each cell represent the standardized mean differences and their 95% confidence intervals for each hypnotic compared with placebo.

AHI, apnea–hypopnea index; REM, rapid eye movement. Boldface values indicate statistically significant results.

Temazepam significantly decreased mean SpO_2_ during TST compared with placebo (Table 2 and Appendix S9). Eszopiclone significantly decreased the AI during TST compared with placebo (Table 2 and Appendix S11). However, no significant differences were observed in AHI during non‐REM sleep, AHI during REM sleep, and mean SpO_2_ nadir during TST between any of the drugs and placebo (Table 2 and Appendices S7,S8,S10).

#### Acceptability, tolerability, and other safety outcomes

No significant differences were observed between any of the drugs and placebo regarding all‐cause discontinuation, adverse event‐related discontinuation, incidence of at least one adverse event, headache, or somnolence (Appendices S12–S16).

#### Network meta‐regression analysis results

We identified no potential confounding factors for the effect sizes in the primary outcomes (Appendices S1,S6).

### Sensitivity analysis excluding CPAP titration studies

The previously observed effect of eszopiclone in decreasing LPS and WASO in the primary network meta‐analysis was no longer significant in this sensitivity analysis (Table 1 and Appendices S3,S4). The previously observed effect of eszopiclone in reducing AHI during TST and AI in the primary network meta‐analysis disappeared in this sensitivity analysis (Table 2 and Appendices S6,S11). Other results of the primary network meta‐analysis were similar to those of this sensitivity analysis (Tables 1, 2 and Appendices S1–S11).

### Subgroup analysis including studies involving only non‐CPAP users

This subgroup analysis revealed that the effect of eszopiclone in decreasing LPS and WASO initially observed in the primary network meta‐analysis disappeared (Table 1 and Appendices S3,S4). Further, this subgroup analysis revealed that the effect of eszopiclone in reducing AHI during TST and AI initially observed in the primary network meta‐analysis disappeared (Table 2 and Appendices S6,S11). Other results of the primary network meta‐analysis were similar to those of this sensitivity analysis (Tables 1, 2 and Appendices S1–S11). However, this subgroup analysis did not assess brotizolam, daridorexant, flurazepam, nitrazepam, ramelteon, temazepam, and triazolam due to the lack of data. Moreover, zolpidem was not included in the analysis of AHI during non‐REM sleep in this subgroup due to the lack of data.

### Subgroup analysis including only CPAP titration studies

Eszopiclone was associated with TST increase, LPS decrease, WASO decrease, SE increase, and AHI decrease during TST compared with placebo (Appendices S1,S3–S6). No significant differences were observed in nightly CPAP use between eszopiclone or zolpidem and placebo (Appendix S17). However, this subgroup analysis did not evaluate brotizolam, daridorexant, flurazepam, lemborexant, nitrazepam, ramelteon, temazepam, triazolam, and zopiclone due to the lack of data.

### Network meta‐analysis by classifying medications according to drug class

Pooled BENZs were associated with decreased mean SpO_2_ during TST compared with placebo (Appendix S9). However, no significant differences were observed in other polysomnography‐measured safety outcomes between pooled BENZs or pooled Z‐drugs and placebo (Appendices S6–S11).

### Heterogeneity, inconsistency, and network meta‐analysis results graded using the CINeMA application

Appendices S1–S17 depict the results for heterogeneity and consistency across all outcomes. Global heterogeneity was high for CPAP use per night on all nights, low to moderate for TST, and low for all other outcomes. Some local heterogeneity was observed for TST, at least one adverse event, and CPAP use per night on all nights in specific comparisons, but not for most other outcomes. Statistical evaluation of incoherence was not possible for 8 out of 17 outcomes due to a lack of head‐to‐head studies. However, no significant global or local inconsistency was observed for the remaining nine outcomes. Most comparisons had ‘some concerns’ regarding within‐study bias. None of the comparisons included 10 or more studies; thus, the assessment of publication bias using a funnel plot was not conducted.^23^ Therefore, all comparisons for publication bias were rated as ‘some concerns’. Comparisons with only indirect evidence were downgraded by one level. Consequently, overall confidence in the evidence in most outcomes was generally rated as low or very low.

## Discussion

To the best of our knowledge, this is the first systematic review and network meta‐analysis to comprehensively assess the effects of various hypnotics on sleep architecture and respiratory parameters in adults with OSA. This provides a novel synthesis of the evidence, whereas the interpretation of our findings must be balanced against several methodological constraints. Specifically, the number of included RCTs and participants remains limited, and the study populations demonstrate heterogeneity regarding insomnia symptoms (i.e., the presence or absence of COMISA), CPAP use, and sleep apnea severity. Such heterogeneity poses challenges in a network meta‐analysis, where the validity of indirect comparisons depends on the transitivity assumption. Our analyses identified no evidence of transitivity violations or significant confounding factors for the primary outcomes; however, the inherent variability across studies might still affect the robustness of the pooled estimates. In addition, the scarcity of available data prevented a formal synthesis of subjective outcomes. In this section, results were discussed by hypnotic class, and the broader limitations of the current evidence base were addressed.

### BENZs

Our network meta‐analysis revealed that temazepam reduced WASO but did not affect TST, REM sleep time, LPS, or SE and decreased mean SpO_2_ during TST. Nitrazepam and triazolam did not influence TST or AHI during TST. Brotizolam did not affect AHI, mean SpO_2_, or mean SpO_2_ nadir during TST; however, its influence on sleep architecture could not be comprehensively assessed due to insufficient data.

Despite grouping by hypnotic class to increase statistical power, our network meta‐analysis revealed that pooled BENZs decreased mean SpO_2_ during TST but did not affect other respiratory outcomes measured with polysomnography. Unlike some guidelines indicating BENZs worsen AHI,^10^, ^11^, ^12^ our network meta‐analysis did not confirm this risk. However, considering the established safety concerns associated with BENZs and Z‐drugs, particularly regarding respiratory function and other adverse effects,^10^, ^11^, ^12^, ^22^, ^73^, ^74^ the administration of these agents in individuals with OSA warrants strict clinical caution.

### DORAs

Lemborexant increased TST, REM sleep time, and SE and decreased LPS and WASO. REM sleep, which is essential for memory consolidation, particularly for procedural, emotional, and spatial memories,^75^ is reduced in patients with OSA.^12^ Furthermore, lemborexant did not affect respiratory safety outcomes and demonstrated superior acceptability and tolerability. Recent network meta‐analyses for adults with primary insomnia demonstrated that lemborexant has a good risk–benefit balance.^22^, ^73^, ^76^, ^77^ DORAs do not carry a risk of dependence or withdrawal symptoms.^22^, ^76^, ^77^ However, several limitations regarding the evidence for lemborexant in this network meta‐analysis warrant consideration. Our results were derived from only three industry‐sponsored trials, which lacked direct head‐to‐head comparisons between different hypnotic agents.^51^, ^52^, ^61^ Notably, these trials excluded adults receiving CPAP therapy, thereby precluding a direct assessment of lemborexant's respiratory safety in this specific population. Consequently, the current data may be insufficient to draw definitive conclusions, and further well‐designed, independent studies are warranted to clarify the utility of lemborexant when administered concomitantly with CPAP therapy.

Our network meta‐analysis revealed that daridorexant increased TST and SE and decreased WASO in adults with OSA; however, daridorexant did not affect REM sleep time or LPS. Daridorexant did not affect respiratory outcomes. However, notably, these findings are derived from a single clinical trial, which may limit their generalizability.^44^

One trial of suvorexant 40 mg/day revealed increased AHI during TST compared with placebo.^71^ However, it was excluded from our network meta‐analysis due to the high dose. Further research is warranted to identify whether approved suvorexant doses affect respiratory function in adults with OSA.

### Ramelteon

Our network meta‐analysis revealed that ramelteon decreased LPS in adults with OSA and did not affect SE and AHI during TST. However, other polysomnography‐based outcomes could not be assessed due to limited data, and the results are based on a single trial.^38^ A recent network meta‐analysis revealed that ramelteon improved LPS in adults with primary insomnia but not TST and WASO.^73^

### Z‐drugs

Our network meta‐analysis revealed that zolpidem increased TST and SE and decreased WASO, with consistent results in sensitivity analysis excluding CPAP titration studies and subgroup analysis including studies that enrolled only non‐CPAP users. Moreover, zolpidem did not affect any safety outcomes measured with polysomnography. However, caution is required due to potential risks similar to those associated with BENZs.

Our network meta‐analysis revealed that eszopiclone increased TST and SE and decreased LPS, WASO, AHI during TST, and AI in adults with OSA. The subgroup analysis, including only CPAP titration studies, revealed that eszopiclone increased TST and SE and decreased LPS, WASO, and AHI during TST. However, the benefits for LPS, WASO, AHI during TST, and AI were not sustained when excluding CPAP titration studies (sensitivity analysis) or focusing on non‐CPAP users (subgroup analysis). The eszopiclone studies in the sensitivity and subgroup analyses were identical. These results indicate that CPAP titration studies may be affecting eszopiclone's results in our network meta‐analysis. CPAP titration may contribute to sleep fragmentation, manifested as frequent awakenings or arousals, owing to pressure‐related factors such as suboptimal pressure settings or device‐related issues, including poor mask fit or low adherence. Consequently, sleep quality may be adversely affected despite effective control of OSA. Under these conditions, our findings should be interpreted with caution, as it remains uncertain whether the observed effects on sleep architecture and respiratory parameters reflect direct or indirect pharmacological actions of the hypnotic agent, mask‐ or device‐related influences, or a combination of these factors, each of which may exert either beneficial or adverse effects. Notably, eszopiclone did not improve CPAP adherence in individuals with OSA, even when administered during CPAP titration.

Zopiclone did not affect sleep architecture. Further, a paucity of data prevented the evaluation of any outcomes associated with polysomnography for zaleplon within the current network meta‐analysis.

### Study limitations

First, our meta‐analysis included a relatively small number of participants and studies. Because only three studies included patients diagnosed with COMISA according to international consensus diagnostic criteria,^38^, ^46^, ^61^ we did not perform a subgroup analysis restricted to this specific population. However, a narrative review of seven observational studies revealed that 39%–58% of individuals with OSA had insomnia symptoms.^7^ However, as highlighted in the recent review,^78^ the COMISA phenotype is the most clinically relevant population for assessing the efficacy and safety of hypnotics in OSA. Consequently, the investigation of patients with confirmed COMISA represents a critical priority for future research. Second, we were unable to perform a meta‐analysis on subjective outcomes, such as insomnia severity, sleep quality, daytime functioning, and quality of life, due to insufficient data. This limitation may indicate that these findings should not be directly extrapolated to patient‐centered clinical decisions. Third, the studies included in our meta‐analysis were primarily of short duration; thus, this limitation is particularly pertinent to safety outcome assessment. Short‐term studies are unlikely to capture adverse events associated with chronic exposure, cumulative dosing, physiological tolerance, and withdrawal‐related phenomena. Notably, approximately 60% of the included studies in our network meta‐analysis reported a duration of only 1–3 days, with a mean duration of 9.63 days and a median of 2 days. Fourth, the sleep laboratory environment may introduce potential confounding variables, whereas positive findings obtained *via* polysomnography are considered to possess high objective reliability and significant clinical use. Fifth, a potential confounding factor exists in studies that involved CPAP titration. Therefore, we conducted sensitivity and subgroup analyses specifically focusing on CPAP titration studies. Further, most of the included studies (60.71%) in this network meta‐analysis involved participants receiving CPAP therapy. CPAP might mask the depressant effects of certain medications on respiratory function; thus, the safety outcomes regarding respiratory stability should be interpreted with caution. Sixth, approximately 50% studies were industry‐sponsored. However, our meta‐regression analysis of this factor (industry‐sponsorship studies *vs*. others) was not associated with the effect sizes in our primary outcomes. Seventh, SUCRA values should be viewed as indicative rather than conclusive, and interpreted with appropriate caution, considering the sparse nature of the network. Eighth, we discovered ‘considerable’ global heterogeneity in CPAP use per night on all nights. This may be associated with the insufficient sample size. Nineth, we utilized crossover data because most studies used a crossover design. Further, the overall risk of bias was rated as ‘low’ or ‘some concerns’, whereas meta‐regression analyses revealed no significant association between these factors and the magnitude of effect sizes for the primary outcomes. Tenth, our analysis did not incorporate novel metrics of OSA severity, including hypoxic burden or heart rate variability, which may provide deeper information into cardiovascular risk because of insufficient data for conducting a network meta‐analysis. Eleventh, this systematic review included only hypnotic agents classified by the US FDA into the major categories for primary insomnia treatment (i.e., BENZs, Z‐drugs, melatonin receptor agonists, and DORAs),^21^ other agents—including doxepin—that are not classified as hypnotics but may improve insomnia symptoms in individuals with OSA warrant assessment in future systematic reviews. Finally, our study did not address several aspects of informed clinical decision‐making, such as integrating pharmacotherapy with other nonpharmacological interventions, including cognitive behavior therapy and cost‐effectiveness analysis.

## Conclusions

Our network meta‐analysis revealed distinct differences across various hypnotics in terms of their effects on sleep architecture and respiratory outcomes in adults with OSA. However, the scarcity of available data prevented a formal synthesis of subjective outcomes. Given this and other inherent methodological limitations, these results warrant a cautious interpretation in the context of clinical decision‐making.

## Author contributions

Prof. T.K. had full access to all the data in the study and takes responsibility for the integrity of the data along with the accuracy of the data analysis. Prof. T.K. and Dr. T.K. were involved in the study concept and design. Prof. Kishi and Dr. Ikuta performed the statistical analysis. All the authors performed acquisition and interpretation of the data and wrote the manuscript. Prof. Iwata and Prof. Kitajima supervised the review.

## Disclosure statement

All authors have no conflicts of interest to declare concerning this study. They also declare any potential competing interests that have arisen in the last 3 years. Prof. Taro Kishi has received speaker's honoraria from Daiichisankyo, Eisai, Janssen, Boehringer Ingelheim, Meiji, Otsuka, Sumitomo, Takeda, Mitsubishi‐Tanabe, Kyowa, Yoshitomi, Shionogi, and Viatris and research grants from a Fujita Health University School of Medicine Research Grant, JSPS KAKENHI (19K08082, 23K06998, and 25K10874), Japan Agency for Medical Research and Development (JP22dk0307107, JP22wm0525024, JP23dk0307122, and 24dk0307129), and the Japanese Ministry of Health, Labour and Welfare (21GC1018). Dr. Ikuta has nothing to disclose. Dr. Sakuma has received speaker's honoraria from Daiichisankyo, Eisai, Janssen, Kyowa, Meiji, Otsuka, Sumitomo, and Takeda and has received a Fujita Health University School of Medicine Research Grant for Early‐Career Scientists, Grant‐in‐Aid for Scientific Research (C)(23K06998), and Japan Agency for Medical Research and Development (JP22dk0307107 and JP23dk0307122). Dr. Hatano received the speaker's honoraria from Meiji and Sumitomo, and has received Grant‐in‐Aid for Early‐Career Scientists (23K14827). Dr. Tatsuhiko Kishi has nothing to disclose. Prof. Kitajima has received speaker honoraria from Eisai, Mitsubishi Tanabe, Otsuka, Takeda, MSD, Meiji, Sumitomo, Shionogi, and Viatris, and research grants from a Fujita Health University School of Medicine Research Grant, Grant‐in‐Aid for Scientific Research(A)(20H00569), and Grant‐in‐Aid for Scientific Research (C) (18K07573, 21K10702, 22K07627, 24K10721, and 25K10825). Prof. Iwata has received speaker's honoraria from Eisai, Janssen, Meiji, Otsuka, Sumitomo, Takeda, Mitsubishi‐Tanabe, and Viatris and research grants from Daiichi Sankyo, Eisai, Meiji, Otsuka, Sumitomo, Takeda, Tanabe‐Mitsubishi, Grant‐in‐Aid for Scientific Research (B)(22H03003), and Japan Agency for Medical Research and Development (JP22wm0425008).

## Supporting information


**Figure S1.** Flow chart showing screening process of literature review.
**Table S1.** Study and patient characteristics of randomized controlled trials.
**Table S2.** PRISMA for network meta‐analyses checklist.
**Table S3.** Transitivity assessment.
**Table S4.** Risk of bias summary for the trials included in the network meta‐analysis.
**Appendix S1.** Total sleep time.
**Appendix S2.** Rapid eye movement sleep time.
**Appendix S3.** Latency to persistent sleep.
**Appendix S4.** Wake after sleep onset.
**Appendix S5.** Sleep efficiency.
**Appendix S6.** Apnea–hypopnea index during total sleep time.
**Appendix S7.** Apnea–hypopnea index during non‐rapid eye movement sleep.
**Appendix S8.** Apnea–hypopnea index during rapid eye movement sleep.
**Appendix S9.** Mean SpO_2_ during total sleep time.
**Appendix S10.** Mean SpO_2_ nadir during total sleep time.
**Appendix S11.** Arousal index during total sleep time.
**Appendix S12.** All‐cause discontinuation.
**Appendix S13.** Adverse event‐related discontinuation.
**Appendix S14.** At least one adverse event.
**Appendix S15.** Headache.
**Appendix S16.** Somnolence.
**Appendix S17.** CPAP use per night on all nights.

## Data Availability

The data that support the findings of this study are available from the corresponding author upon reasonable request.
