# Supplementary material for: Comparative effects of hypnotic agents on sleep architecture and respiratory outcomes in obstructive sleep apnea: A systematic review and network meta‐analysis
Source: Psychiatry Clin Neurosci. 2026 Feb 10;80(5):390–7. doi: 10.1111/pcn.70036 (PMC13139812; doi:10.1111/pcn.70036)
Supplement: Supplementary file 1 — Figure S1. Flow chart showing screening process of literature review. Table S1. Study and patient characteristics of randomized controlled trials. Table S2. PRISMA for network meta‐analyses checklist. Table S3. Transitivity assessment. Table S4. Risk of bias summary for the trials included in the network meta‐analysis. Appendix S1. Total sleep time. Appendix S2. Rapid eye movement sleep time. Appendix S3. Latency to persistent sleep. Appendix S4. Wake after sleep onset. Appendix S5. Sleep efficiency. Appendix S6. Apnea–hypopnea index during total sleep time. Appendix S7. Apnea–hypopnea index during non‐rapid eye movement sleep. Appendix S8. Apnea–hypopnea index during rapid eye movement sleep. Appendix S9. Mean SpO2 during total sleep time. Appendix S10. Mean SpO2 nadir during total sleep time. Appendix S11. Arousal index during total sleep time. Appendix S12. All‐cause discontinuation. Appendix S13. Adverse event‐related discontinuation. Appendix S14. At least one adverse event. Appendix S15. Headache. Appendix S16. Somnolence. Appendix S17. CPAP use per night on all nights. [file PCN-80-390-s001.pdf]

Figure S1. Flow chart showing screening process of literature review

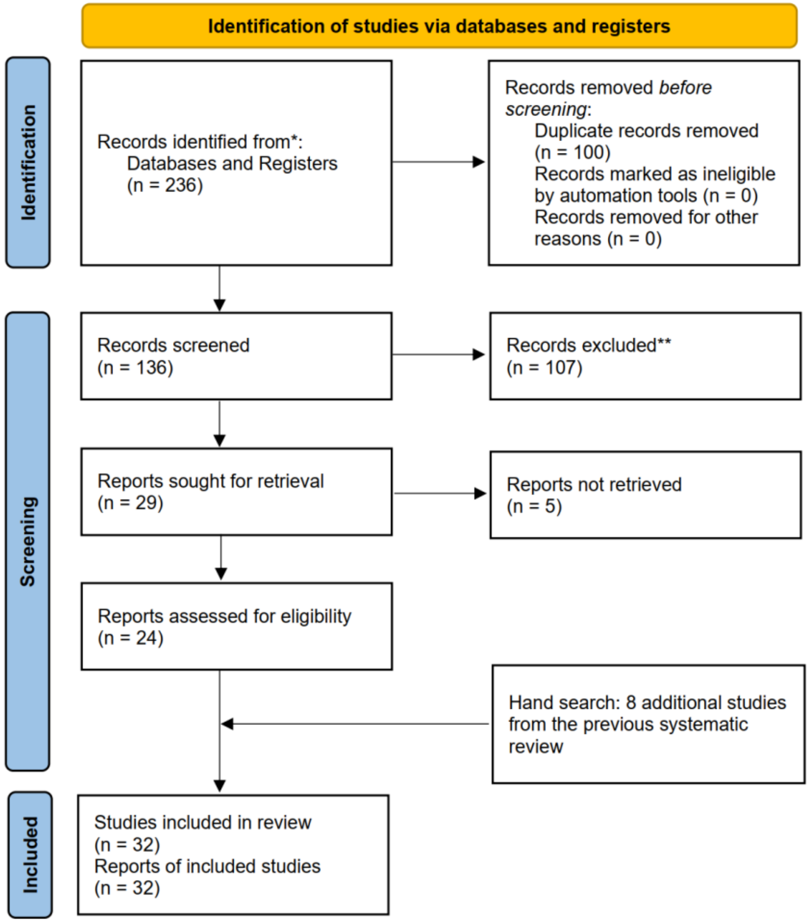

We searched the PubMed, the Cochrane Library, and Embase databases for studies published before December 1, 2025, without language restriction. The search terms for PubMed and the Cochrane Library included (Obstructive Sleep Apnea Syndrome OR obstructive sleep apnea OR OSA OR OSAS OR SAS) AND (random*) AND (Daridorexant OR Lemborexant OR Suvorexant OR Zolpidem OR Eszopiclone OR Zopiclone OR Zaleplon OR Ramelteon). The search terms for Embase included ('obstructive sleep apnea'/exp OR 'obstructive sleep apnea') AND ('randomized controlled trial'/exp OR 'randomized controlled trial') AND ('daridorexant'/exp OR 'daridorexant' OR 'lemborexant'/exp OR 'lemborexant' OR 'suvorexant'/exp OR 'suvorexant' OR 'zolpidem'/exp OR 'zolpidem' OR 'eszopiclone'/exp OR 'eszopiclone' OR 'zopiclone'/exp OR 'zopiclone' OR 'zaleplon'/exp OR 'zaleplon' OR 'ramelteon'/exp OR 'ramelteon'). We selected studies of benzodiazepines from the review articles.

Review articles and clinical practice guidelines that we read for the literature search

1. Akashiba T, Inoue Y, Uchimura N, et al. Sleep Apnea Syndrome (SAS) Clinical Practice Guidelines 2020. Respir Investig. Jan 2022;60(1):3-32. doi:10.1016/j.resinv.2021.08.010

2. Akashiba T, Inoue Y, Uchimura N, et al. Sleep Apnea Syndrome (SAS) Clinical Practice Guidelines 2020. Sleep Biol Rhythms. Jan 2022;20(1):5-37. doi:10.1007/s41105-021-00353-6

3. ASA. Australasian Sleep Association. https://wwwsleepprimarycareresourcesorgau/. 2025;

4. Gaisl T, Haile SR, Thiel S, Osswald M, Kohler M. Efficacy of pharmacotherapy for OSA in adults: A systematic review and network meta-analysis. Sleep Med Rev. Aug 2019;46:74-86. doi:10.1016/j.smrv.2019.04.009

5. Gottlieb DJ, Punjabi NM. Diagnosis and Management of Obstructive Sleep Apnea: A Review. JAMA. Apr 14 2020;323(14):1389-1400. doi:10.1001/jama.2020.3514

6. Kasai T, Kohno T, Shimizu W, et al. JCS 2023 Guideline on Diagnosis and Treatment of Sleep Disordered Breathing in Cardiovascular Disease. Circ J. Oct 25 2024;88(11):1865-1935. doi:10.1253/circj.CJ-23-0489

7. Lee YC, Lu CT, Chuang LP, et al. Pharmacotherapy for obstructive sleep apnea - A systematic review and meta-analysis of randomized controlled trials. Sleep Med Rev. Aug 2023;70:101809. doi:10.1016/j.smrv.2023.101809

8. Mason M, Cates CJ, Smith I. Effects of opioid, hypnotic and sedating medications on sleep-disordered breathing in adults with obstructive sleep apnoea. Cochrane Database Syst Rev. Jul 14 2015;(7):CD011090. doi:10.1002/14651858.CD011090.pub2

9. Mason M, Welsh EJ, Smith I. Drug therapy for obstructive sleep apnoea in adults. Cochrane Database Syst Rev. May 31 2013;2013(5):CD003002. doi:10.1002/14651858.CD003002.pub3

10. Neshat SS, Heidari A, Henriquez-Beltran M, et al. Evaluating pharmacological treatments for excessive daytime sleepiness in obstructive sleep apnea: A comprehensive network meta-analysis and systematic review. Sleep Med Rev. Aug 2024;76:101934. doi:10.1016/j.smrv.2024.101934

11. Riemann D, Espie CA, Altena E, et al. The European Insomnia Guideline: An update on the diagnosis and treatment of insomnia 2023. J Sleep Res. Dec 2023;32(6):e14035. doi:10.1111/jsr.14035

12. SHF. Sleep Apnoea. Sleep Health Foundation. 2025;https://www.sleephealthfoundation.org.au/

13. Wang D, Tang Y, Chen Y, et al. The effect of non-benzodiazepine sedative hypnotics on CPAP adherence in patients with OSA: a systematic review and meta-analysis. Sleep. Aug 13 2021;44(8)doi:10.1093/sleep/zsab077

14. Yeh WC, Li YS, Chang YP, Hsu CY. The efficacy and safety of dual orexin receptor antagonists in obstructive sleep apnea: A systematic review and meta-analysis of randomised controlled trials. J Sleep Res. Nov 14 2024:e14399. doi:10.1111/jsr.14399

Articles which we have found in the current literature search (K = 24)

1. Berry RB, Patel PB. Effect of zolpidem on the efficacy of continuous positive airway pressure as treatment for obstructive sleep apnea. Sleep. Aug 2006;29(8):1052-6. doi:10.1093/sleep/29.8.1052

2. Boof ML, Dingemanse J, Lederer K, Fietze I, Ufer M. Effect of the new dual orexin receptor antagonist daridorexant on nighttime respiratory function and sleep in patients with mild and moderate obstructive sleep apnea. Sleep. Jun 11 2021;44(6)doi:10.1093/sleep/zsaa275

3. Bradshaw DA, Ruff GA, Murphy DP. An oral hypnotic medication does not improve continuous positive airway pressure compliance in men with obstructive sleep apnea. Chest. Nov 2006;130(5):1369-76. doi:10.1378/chest.130.5.1369

4. Carberry JC, Fisher LP, Grunstein RR, et al. Role of common hypnotics on the phenotypic causes of obstructive sleep apnoea: paradoxical effects of zolpidem. Eur Respir J. Dec 2017;50(6)doi:10.1183/13993003.01344-2017

5. Carter SG, Berger MS, Carberry JC, et al. Zopiclone Increases the Arousal Threshold without Impairing Genioglossus Activity in Obstructive Sleep Apnea. Sleep. Apr 1 2016;39(4):757-66. doi:10.5665/sleep.5622

6. Carter SG, Carberry JC, Cho G, et al. Effect of 1 month of zopiclone on obstructive sleep apnoea severity and symptoms: a randomised controlled trial. Eur Respir J. Jul 2018;52(1)doi:10.1183/13993003.00149-2018

7. Carter SG, Carberry JC, Grunstein RR, Eckert DJ. Randomized Trial on the Effects of High-Dose Zopiclone on OSA Severity, Upper Airway Physiology, and Alertness. Chest. Jul 2020;158(1):374-385. doi:10.1016/j.chest.2020.02.057

8. Cheng JY, Filippov G, Moline M, Zammit GK, Bsharat M, Hall N. Respiratory safety of lemborexant in healthy adult and elderly subjects with mild obstructive sleep apnea: A randomized, double-blind, placebo-controlled, crossover study. J Sleep Res. Aug 2020;29(4):e13021. doi:10.1111/jsr.13021

9. Cheng JY, Lorch D, Lowe AD, et al. A randomized, double-blind, placebo-controlled, crossover study of respiratory safety of lemborexant in moderate to severe obstructive sleep apnea. J Clin Sleep Med. Jan 1 2024;20(1):57-65. doi:10.5664/jcsm.10788

10. Corser B, Eves E, Warren-McCormick J, Rucosky G. Effects of atomoxetine plus a hypnotic on obstructive sleep apnea severity in patients with a moderately collapsible pharyngeal airway. J Clin Sleep Med. Jun 1 2023;19(6):1035-1042. doi:10.5664/jcsm.10464

11. Edwards BA, Sands SA, Owens RL, et al. The Combination of Supplemental Oxygen and a Hypnotic Markedly Improves Obstructive Sleep Apnea in Patients with a Mild to Moderate Upper Airway Collapsibility. Sleep. Nov 1 2016;39(11):1973-1983. doi:10.5665/sleep.6226

12. George CF, Feldman N, Inhaber N, et al. A safety trial of sodium oxybate in patients with obstructive sleep apnea: Acute effects on sleep-disordered breathing. Sleep Med. Jan 2010;11(1):38-42. doi:10.1016/j.sleep.2009.06.006

13. Gooneratne NS, Gehrman P, Gurubhagavatula I, Al-Shehabi E, Marie E, Schwab R. Effectiveness of ramelteon for insomnia symptoms in older adults with obstructive sleep apnea: a randomized placebo-controlled pilot study. J Clin Sleep Med. Dec 15 2010;6(6):572-80.

14. Kryger M, Wang-Weigand S, Roth T. Safety of ramelteon in individuals with mild to moderate obstructive sleep apnea. Sleep Breath. Sep 2007;11(3):159-64. doi:10.1007/s11325-006-0096-4

15. Kushida CA, Zammit GK, Cheng JY, Kumar D, Moline M. Effect of lemborexant on sleep architecture in participants with insomnia disorder and mild obstructive sleep apnea. Sleep Med. Mar 2025;127:170-177. doi:10.1016/j.sleep.2024.12.023

16. Lettieri CJ, Collen JF, Eliasson AH, Quast TM. Sedative use during continuous positive airway pressure titration improves subsequent compliance: a randomized, double-blind, placebo-controlled trial. Chest. Nov 2009;136(5):1263-1268. doi:10.1378/chest.09-0811

17. Lettieri CJ, Quast TN, Eliasson AH, Andrada T. Eszopiclone improves overnight polysomnography and continuous positive airway pressure titration: a prospective, randomized, placebo-controlled trial. Sleep. Sep 2008;31(9):1310-6.

18. Lettieri CJ, Shah AA, Holley AB, et al. Effects of a short course of eszopiclone on continuous positive airway pressure adherence: a randomized trial. Ann Intern Med. Nov 17 2009;151(10):696-702. doi:10.7326/0003-4819-151-10-200911170-00006

19. Messineo L, Carter SG, Taranto-Montemurro L, et al. Addition of zolpidem to combination therapy with atomoxetine-oxybutynin increases sleep efficiency and the respiratory arousal threshold in obstructive sleep apnoea: A randomized trial. Respirology. Sep 2021;26(9):878-886. doi:10.1111/resp.14110

20. Messineo L, Eckert DJ, Lim R, et al. Zolpidem increases sleep efficiency and the respiratory arousal threshold without changing sleep apnoea severity and pharyngeal muscle activity. J Physiol. Oct 2020;598(20):4681-4692. doi:10.1113/JP280173

21. Park JG, Olson EJ, Morgenthaler TI. Impact of zaleplon on continuous positive airway pressure therapy compliance. J Clin Sleep Med. May 15 2013;9(5):439-44. doi:10.5664/jcsm.2660

22. Rosenberg R, Roach JM, Scharf M, Amato DA. A pilot study evaluating acute use of eszopiclone in patients with mild to moderate obstructive sleep apnea syndrome. Sleep Med. Aug 2007;8(5):464-70. doi:10.1016/j.sleep.2006.10.007

23. Schmickl CN, Orr JE, Alex RM, et al. Combination Drug Therapy with Acetazolamide, Eszopiclone +/- Venlafaxine for Obstructive Sleep Apnea (RESCUE-Combo): A

Randomized, Double-Blind, Placebo-controlled Clinical Trial. Ann Am Thorac Soc. Feb 2025;22(2):263-273. doi:10.1513/AnnalsATS.202407-736OC

24. Sun H, Palcza J, Card D, et al. Effects of Suvorexant, an Orexin Receptor Antagonist, on Respiration during Sleep In Patients with Obstructive Sleep Apnea. J Clin Sleep Med. Jan 2016;12(1):9-17. doi:10.5664/jcsm.5382

Articles included in the review articles and clinical practice guidelines (K = 8)

1. Berry RB, Kouchi K, Bower J, Prosise G, Light RW. Triazolam in patients with obstructive sleep apnea. Am J Respir Crit Care Med. Feb 1995;151(2 Pt 1):450-4. doi:10.1164/ajrccm.151.2.7842205

2. Camacho ME, Morin CM. The effect of temazepam on respiration in elderly insomniacs with mild sleep apnea. Sleep. Oct 1995;18(8):644-5. doi:10.1093/sleep/18.8.644

3. Cirignotta F, Mondini S, Gerardi R, Zucconi M. Effect of brotizolam on sleep-disordered breathing in heavy snorers with obstructive apnea. Current Therapeutic Research, Clinical and Experimental. 1992;51:360-6.

4. Cirignotta F, Mondini S, Zucconi M, Gerardi R, Farolfi A, Lugaresi E. Zolpidem-polysomnographic study of the effect of a new hypnotic drug in sleep apnea syndrome. Pharmacol Biochem Behav. Apr 1988;29(4):807-9. doi:10.1016/0091-3057(88)90212-2

5. Eckert DJ, Owens RL, Kehlmann GB, et al. Eszopiclone increases the respiratory arousal threshold and lowers the apnoea/hypopnoea index in obstructive sleep apnoea patients with a low arousal threshold. Clin Sci (Lond). Jun 2011;120(12):505-14. doi:10.1042/CS20100588

6. Hoijer U, Hedner J, Ejnell H, Grunstein R, Odelberg E, Elam M. Nitrazepam in patients with sleep apnoea: a double-blind placebo-controlled study. Eur Respir J. Nov 1994;7(11):2011-5.

7. Shah A, Lettieri C, Holley A, Kelly W, Roop S. Eszopiclone improves short and intermediate term continuous positive airway pressure adherence. Sleep. 2009;32(Suppl. S):A184-A185.

8. Wang D, Marshall NS, Duffin J, et al. Phenotyping interindividual variability in obstructive sleep apnoea response to temazepam using ventilatory chemoreflexes during wakefulness. J Sleep Res. Dec 2011;20(4):526-32. doi:10.1111/j.1365-2869.2011.00931.x

Table S1. Study and patient characteristics of randomized controlled trials

Study (country, sponsor)	Trial ID	Total n	Study duration [‡]	Diagnosis (key inclusion criteria, diagnostic criteria)	Washout period [§]	Mean age (y ± SD)	Male (%)	Mean AHI±SD (events/h) at baseline	Mean BMI±SD (kg/m^2)	Treatment (mg/d, dosage)	CPAP used?	AHI at the study endpoint	TST at the study endpoint
Berry 1995 (USA, NR) [†]	NR	12	1 d	Severe OSA (NR, NR)	1 d	46.6±14.1	100	NR	NR	TRI (0.25, fixed) or PLA	Yes (some PT received CPAP)	TRI was similar to PLA.	TRI was similar to PLA.
Berry 2006 (USA, academia) [†]	NR	16	1 d	Severe OSA (AHI>30, NR)	6 d	49.4±12.4	87.5	NR	36.1±4.8	ZOL (10, fixed) or PLA	Yes (all PT received CPAP)	ZOL was similar to PLA.	ZOL was similar to PLA.
Boof 2021 (Germany, industry) [†]	NCT03765294	28	5 d	Mild to moderate OSA (AHI: 5 to <30, AASM)	1-2 w	60.1	64.3	17.0±8.49	28.6	DAR (50, fixed) or PLA	Yes (some PT received CPAP)	DAR was similar to PLA.	DAR was longer than PLA.
Bradshaw 2006 (USA, academia)	NR	72	14 d	OSA (AHI>5, NR)	NA	38.1±7.4	100	41.8±27.6	32.1±5.2	ZOL (10, fixed), PLA or ST	Yes (all PT underwent CPAP titration study)	NR	NR
Camacho 1995 (USA, academia)	NR	15	56 d	Mild OSA (AHI: 5 to 15, NR) with insomnia (DSM-III-R)	NA	65±5.8	60.0	9.0±4.0	NR	TEM (15-30, flexible)+BT or PLA+BT	Yes (some PT received CPAP)	TEM was similar to PLA (RDI).	TEM was similar to PLA.
Carberry 2017 (Australia, academia) [†]	ACTRN12612001004853	28	1 d	OSA (AHI>10, NR) [‡ ‡]	7 d	40±15	66.7	19±22	28±7	TEM (10, fixed), ZOL (10, fixed), ZOP (7.5, fixed) or PLA	Yes (all PT received CPAP)	NR	NR
Carter 2016 (Australia, academia) [†]	ACTRN12614000364673	14	1 d	OSA (AHI≥5, NR)	7 d	49±3	75.0	41±8	NR	ZOP (7.5, fixed) or PLA	No	ZOP was similar to PLA.	NR
Carter 2018 (Australia, academia)	ANZCTRN12613001106729	31	30 d	OSA (AHI≥5, NR)	NA	47.9±11,0	83.3	21.9±10.8	28.5±4.0	ZOP (7.5, fixed) or PLA	No	ZOP was similar to PLA.	ZOP was similar to PLA.
Carter 2020 (Australia, academia) [†]	ACTRN12617000988358	30	1 d	OSA (AHI≥5, NR)	7 d	45 ±13	75.0	29±20	29±5	ZOP (15, fixed) or PLA	No	ZOP was similar to PLA.	NR
Cheng 2020 (USA, industry) [†]	NCT03471871	39	8 d	Mild OSA (AHI: 5 to <15, ICSD)	≥14 d	57.2±13.1	38.5	9.00±3.10	29.1±4.6	LEM (10, fixed) or PLA	No	LEM was similar to PLA.	NR
Cheng 2024 (USA, industry) [†]	NCT04647383	33	8 d	Moderate to severe OSA (AHI≥15, ICSD)	≥14 d	60.6±9.2	69.7	44.2±24.2	31.9±4.4	LEM (10, fixed) or PLA	No	LEM was similar to PLA.	NR
Cirignotta 1988 (Italy, NR) [†]	NR	12	1 d	Mild OSA (NR, Lugaresi's criteria)	6 d	49	91.7	NR	NR	ZOL (20, fixed), FLU (30, fixed) or PLA	Yes (some PT received CPAP)	ZOL and FLU were similar to PLA.	ZOL and FLU were longer than PLA.
Cirignotta 1992 (Italy, industry) [†]	NR	12	2 d	Mild OSA (NR, NR)	10	49	100	NR	28.2	BLO (0.25, fixed), FLU (30, fixed) or PLA	Yes (some PT received CPAP)	BLO and FLU were similar to PLA (RDI).	NR
Corser 2023 (USA, industry) [†]	NCT04645524	15	1 d	OSA (AHI: 10 to 55, NR)	1 w	52	73.3	15.2	33.6	LEM (10, fixed)+ATO (80, fixed), TRA (100, fixed)+ATO (80, fixed), or	No	ATO+TRA was lower than PLA. ATO+LEM was similar to PLA.	ATO+TRA and ATO+LEM were similar to PLA.

										PLA			
Eckert 2011 (USA, industry) †	NCT01102270	17	1 d	OSA (AHI>5, NR)	1 w	45±15	58.8	31	33	ESZ (3, fixed) or PLA	No	ESZ was lower than PLA.	ESZ was longer than PLA.
Edwards 2016 (USA, academia) †	NCT01633827	22	≥2 d	OSA (AHI>10, NR)	1 w	50.9±11.8	60.0	NR	31.6	ESZ (3, fixed)+40%O_2 or PLA	Yes (27% of PT received CPAP)	ESZ+40%O_2 was lower than PLA.	ESZ+40%O_2 was longer than PLA.
George 2010 (USA and Canada, industry) †	NR	60	1 d	Mild to moderate OSA (AHI: 10 to 40, NR)	No	Range: 24–77	76.7	24.7±9.2	Range: 23.8–44.6	ZOL (10, fixed), SOD (9000, fixed), SOD (9000, fixed)+MOD (200, fixed), or PLA	No	SOD, SOD+MOD and ZOL were similar to PLA.	SOD and ZOL were longer than PLA. SOD+MOD was similar to PLA.
Gooneratne 2010 (USA, industry)	NR	27	30 d	OSA (AHI≥5, ICSD) with insomnia (DSM-IV-TR)	NA	71.7±4.5	61.9	12.0±11.0	27.6±5.6	RAM (8, fixed) or PLA	Yes (all PT received APAP)	RAM was similar to PLA.	NR
Höijer 1994 (Sweden, academia) †	NR	14	3 d	Mild to moderate OSA (60–180 apnoeas per 6 h of self-reported sleep time, NR)	7 d	47	85.7	15	27	NIT (5, fixed), NIT (10, fixed), or PLA	Yes (some PT received CPAP)	NIT5 and NIT10 were similar to PLA.	NIT5 and NIT10 were longer than PLA.
Kryger 2007 (USA, industry) †	NR	26	1 d	Mild to moderate OSA (AHI: 5 to 20, NR)	5-12 d	47.4±9.45	69.2	12.2±7.7	30.2±4.8	RAM (16. fixed) or PLA	No	RAM was similar to PLA.	RAM was similar to PLA.
Kushida 2025 (International, industry) † †	NCT02783729	410	30 d	Mild OSA (AHI: 5 to <15, AASM) with insomnia (DSM-5)	NA	65.0±7.1	16.1	9.3±2.9	28.1±4.8	LEM (5, fixed), LEM (10, fixed), ZOL-ER (6.25, fixed) or PLA	No	NR	LEM5, LEM10 and ZOL-ER were longer than PLA.
Lettieri 2008 (USA, industry)	NCT00507117	226	1 d	Undergoing a diagnostic PSG for suspected OSA or a CPAP titration study to treat OSA previously diagnosed by PSG (NR)	NA	44.3±10.0	72.6	29.3±28.4	30.3±4.9	ESZ (3, fixed) or PLA	Yes (66% of PT underwent CPAP titration study)	ESZ was similar to PLA.	ESZ was longer than PLA.
Lettieri 2009 (Ann Intern Med) (USA, industry)	NCT00612157	160	14 d	Newly diagnosed with OSA who are prescribed CPAP (AASM)	NA	45.7±7.3	78.5	36.4±23.1	30.4±4.0	ESZ (3, fixed) or PLA	Yes (all PT underwent CPAP titration study)	NR	NR
Lettieri 2009 (Chest) (USA, academia)	NCT00507117	117	1 d	Newly diagnosed with OSA who are prescribed CPAP (AASM)	NA	44.9±6.7	78.6	29.2±24.3	NR	ESZ (3, fixed) or PLA	Yes (all PT underwent CPAP titration study)	ESZ was similar to PLA.	ESZ was longer than PLA.
Messineo 2020 (Australia, academia) †	ACTRN12618001287224	20	1 d	OSA (AHI>10, NR)	7 d	47±12	89.5	27.2±16.1	29.4±4.5	ZOL (10, fixed) or PLA	No	ZOL was similar to PLA.	ZOL was longer than PLA
Messineo 2021 (Australia, academia) †	ACTRN12619001427167	13	1 d	OSA (AHI>10, NR)	7 d	55±14	83.3	NR	27±5	ZOL (10, fixed)+ATO (80, fixed)+OXY (5, fixed) or ATO (80, fixed)+OXY (5,	No	ATO+OXY+ZOL was similar to ATO+OXY +PLA.	ATO+OXY+ZOL was longer than ATO+OXY +PLA.

										fixed)+PLA			
Park 2013 (USA, academia)	NR	176	1 d	OSA (AHI≥5, NR)	NA	49.8±11.3	68.7	NR	36.0±8.7	ZAL (10, fixed) or PLA	Yes (all PT underwent CPAP titration study)	ZAL was similar to PLA.	NR
Rosenberg 2007 (USA, industry)[†]	NR	22	2 d	Mild to moderate OSA required CPAP (AHI: 10 to 40, NR)	5-7 d	48.4	68.2	17.2±8.8	35.2	ESZ (3, fixed) or PLA	No	ESZ was similar to PLA.	ESZ was longer than PLA.
Schmickl 2025 (USA, academia)[†]	NCT04639193	20	3 d	Untreated OSA (AHI>15, NR)	4-10 d	49	80.0	39.2	29.1	ESZ (2, fixed)+ACE (500, fixed) or PLA	NR	ACE+ESZ was lower than PLA.	ACE+ESZ was longer than PLA.
Shah 2009 (NR, NR) ¶	NR	136	14 d	Newly diagnosed with OSA who are prescribed CPAP (NR)	NA	45.6±9.0	NR	36.1±27.7	30.4±5.3	ESZ (3, fixed) or PLA	Yes (all PT underwent CPAP titration study)	NR	NR
Sun 2016 (USA, industry)[†]	NCT01300455	26	4 d	Mild to moderate OSA (AHI: 5 to 29, ICSD)	≥7 d	49	73.1	15.2	30.2	SUV (40, fixed) or PLA	No	SUV was higher than PLA.	SUV was longer than PLA.
Wang 2011 (Australia, NR)[†]	ACTRN12608000318381	22	1 d	Mild to moderate OSA (AHI: 5 to 30, NR)	7 d	44±11.6	100	16.8±14.1	27.5±7.5	TEM (10, fixed) or PLA	Yes (some PT received CPAP)	TEM was similar to PLA.	TEM was similar to PLA.

AASM: American Academy of Sleep Medicine, ACE: acetazolamide, AHI: apnea-hypopnea index, AIM : Annals of Internal Medicine, APAP: auto-titrating positive airway pressure, ATO: atomoxetine, BLO: brotizolam, BMI: body mass index, BT: behavior therapy, CHE: Chest, CPAP: continuous positive airway pressure, DAR: daridorexant, DSM(-TR): Diagnostic and Statistical Manual of Mental Disorders(-Text Revision), ESZ: eszopiclone, FLU: flurazepam, ICSD: International Classification of Sleep Disorders, LEM: lemborexant, MOD: modafinil, NA: not applicable, NIT: nitrazepam, NR: not report, O_2: oxygen, OSA: obstructive sleep apnoea, OXY: oxybutynin, PLA: placebo, PSG: polysomnogram, PT: patient, RAM: ramelteon, RDI: respiratory disturbance index, SOD: sodium oxybate, ST: standard treatment (no active drug or placebo pill), SUV: suvorexant, TEM: temazepam, TRA: trazodone, TRI: triazolam, USA: United States of America, VEN: venlafaxine, ZAL: zaleplon, ZOL(-ER): zolpidem(-extended release), ZOP: zopiclone

† Crossover study. The information at baseline in the first phase is shown.

‡ The number of days the participants have taken the medication.

§ The information is for the crossover study.

¶ Conference abstract

† † Post hoc study

‡ ‡ 47.6% of patients were healthy volunteers.

Table S2. PRISMA for network meta-analyses checklist

Section/Topic	Item #	Checklist Item	Reported on Page #
**TITLE**			
Title	1	Identify the report as a systematic review *incorporating a network meta-analysis (or related form of meta-analysis).*	1
**ABSTRACT**			
Structured summary	2	Provide a structured summary including, as applicable:<br>**Background:** main objectives<br>**Methods:** data sources; study eligibility criteria, participants, and interventions; study appraisal; and *synthesis methods, such as network meta-analysis.*<br>**Results:** number of studies and participants identified; summary estimates with corresponding confidence/credible intervals; *treatment rankings may also be discussed. Authors may choose to summarize pairwise comparisons against a chosen treatment included in their analyses for brevity.*<br>**Discussion/Conclusions:** limitations; conclusions and implications of findings.<br>**Other:** primary source of funding; systematic review registration number with registry name.	3-
**INTRODUCTION**			
Rationale	3	Describe the rationale for the review in the context of what is already known*, including mention of why a network meta-analysis has been conducted.*	5-
Objectives	4	Provide an explicit statement of questions being addressed, with reference to participants, interventions, comparisons, outcomes, and study design (PICOS).	5-
**METHODS**			
Protocol and registration	5	Indicate whether a review protocol exists and if and where it can be accessed (e.g., Web address); and, if available, provide registration information, including registration number.	6-
Eligibility criteria	6	Specify study characteristics (e.g., PICOS, length of follow-up) and report characteristics (e.g., years considered, language, publication status) used as criteria for eligibility, giving rationale. *Clearly describe eligible treatments included in the treatment network, and note whether any have been clustered or merged into the same node (with justification).*	6-
Information sources	7	Describe all information sources (e.g., databases with dates of coverage, contact with study authors to identify additional studies) in the search and date last searched.	6-
Search	8	Present full electronic search strategy for at least one database, including any limits used, such that it could be repeated.	6-
Study selection	9	State the process for selecting studies (i.e., screening, eligibility, included in systematic review, and, if applicable, included in the meta-analysis).	6-
Data collection process	10	Describe method of data extraction from reports (e.g., piloted forms, independently, in duplicate) and any processes for obtaining and confirming data from investigators.	6-
Data items	11	List and define all variables for which data were sought (e.g., PICOS, funding sources) and any assumptions and simplifications made.	6-

**Geometry of the network**	**S1**	Describe methods used to explore the geometry of the treatment network under study and potential biases related to it. This should include how the evidence base has been graphically summarized for presentation, and what characteristics were compiled and used to describe the evidence base to readers.	6-
Risk of bias within individual studies	12	Describe methods used for assessing risk of bias of individual studies (including specification of whether this was done at the study or outcome level), and how this information is to be used in any data synthesis.	6-
Summary measures	13	State the principal summary measures (e.g., risk ratio, difference in means). *Also describe the use of additional summary measures assessed, such as treatment rankings and surface under the cumulative ranking curve (SUCRA)* values, as well as modified approaches used to present summary findings from meta-analyses.*	6-
Planned methods of analysis	14	Describe the methods of handling data and combining results of studies for each network meta-analysis. This should include, but not be limited to:<br>• *Handling of multi-arm trials;*<br>• *Selection of variance structure;*<br>• *Selection of prior distributions in Bayesian analyses; and*<br>• *Assessment of model fit.*	6-
**Assessment of Inconsistency**	**S2**	Describe the statistical methods used to evaluate the agreement of direct and indirect evidence in the treatment network(s) studied. Describe efforts taken to address its presence when found.	6-
Risk of bias across studies	15	Specify any assessment of risk of bias that may affect the cumulative evidence (e.g., publication bias, selective reporting within studies).	6-
Additional analyses	16	Describe methods of additional analyses if done, indicating which were pre-specified. This may include, but not be limited to, the following:<br>• Sensitivity or subgroup analyses;<br>• Meta-regression analyses;<br>• *Alternative formulations of the treatment network; and*<br>• *Use of alternative prior distributions for Bayesian analyses (if applicable).*	6-
**RESULTS†**			
Study selection	17	Give numbers of studies screened, assessed for eligibility, and included in the review, with reasons for exclusions at each stage, ideally with a flow diagram.	10-
**Presentation of network structure**	**S3**	Provide a network graph of the included studies to enable visualization of the geometry of the treatment network.	10-
**Summary of network geometry**	**S4**	Provide a brief overview of characteristics of the treatment network. This may include commentary on the abundance of trials and randomized patients for the different interventions and pairwise comparisons in the network, gaps of evidence in the treatment network, and potential biases reflected by the network structure.	10-
Study characteristics	18	For each study, present characteristics for which data were extracted (e.g., study size, PICOS, follow-up period) and provide the citations.	10-
Risk of bias within studies	19	Present data on risk of bias of each study and, if available, any outcome level assessment.	10-

	Results of individual studies	20	For all outcomes considered (benefits or harms), present, for each study: 1) simple summary data for each intervention group, and 2) effect estimates and confidence intervals. *Modified approaches may be needed to deal with information from larger networks.*	10-
	Synthesis of results	21	Present results of each meta-analysis done, including confidence/credible intervals. *In larger networks, authors may focus on comparisons versus a particular comparator (e.g. placebo or standard care), with full findings presented in an appendix. League tables and forest plots may be considered to summarize pairwise comparisons.* If additional summary measures were explored (such as treatment rankings), these should also be presented.	10-
	**Exploration for inconsistency**	**S5**	Describe results from investigations of inconsistency. This may include such information as measures of model fit to compare consistency and inconsistency models, *P* values from statistical tests, or summary of inconsistency estimates from different parts of the treatment network.	10-
	Risk of bias across studies	22	Present results of any assessment of risk of bias across studies for the evidence base being studied.	10-
	Results of additional analyses	23	Give results of additional analyses, if done (e.g., sensitivity or subgroup analyses, meta-regression analyses*, alternative network geometries studied, alternative choice of prior distributions for Bayesian analyses,* and so forth).	10-
**DISCUSSION**				
	Summary of evidence	24	Summarize the main findings, including the strength of evidence for each main outcome; consider their relevance to key groups (e.g., healthcare providers, users, and policy-makers).	14-
	Limitations	25	Discuss limitations at study and outcome level (e.g., risk of bias), and at review level (e.g., incomplete retrieval of identified research, reporting bias). *Comment on the validity of the assumptions, such as transitivity and consistency. Comment on any concerns regarding network geometry (e.g., avoidance of certain comparisons).*	14-
	Conclusions	26	Provide a general interpretation of the results in the context of other evidence, and implications for future research.	14-
**FUNDING**				17
	Funding	27	Describe sources of funding for the systematic review and other support (e.g., supply of data); role of funders for the systematic review. This should also include information regarding whether funding has been received from manufacturers of treatments in the network and/or whether some of the authors are content experts with professional conflicts of interest that could affect use of treatments in the network.	

Table S3. Transitivity assessment

	Boxplot	Kruskal–Wallis equality of populations rank test for continuous variables or the Pearson chi-square test for binary and categorical variables (or the Fisher's exact test whether >20% of cells had an expected frequency below 5).*
Proportion of males	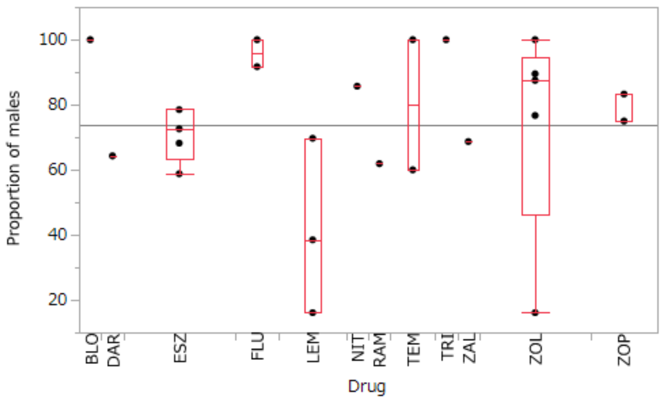	Chi-squared with ties = 13.96 (df = 11), p = 0.24
Mean age (years)	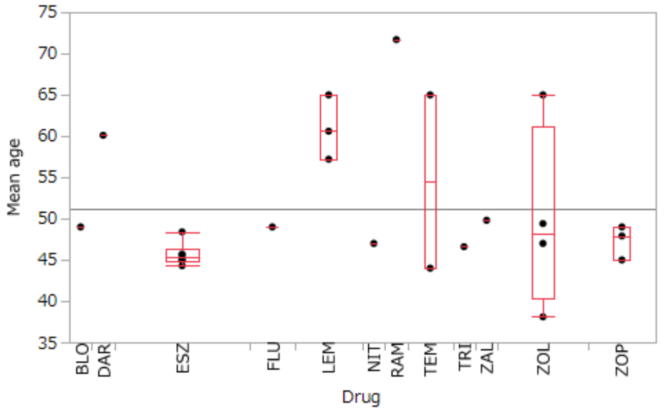	Chi-squared with ties = 13.64 (df = 11), p = 0.25

Mean AHI (events/h)	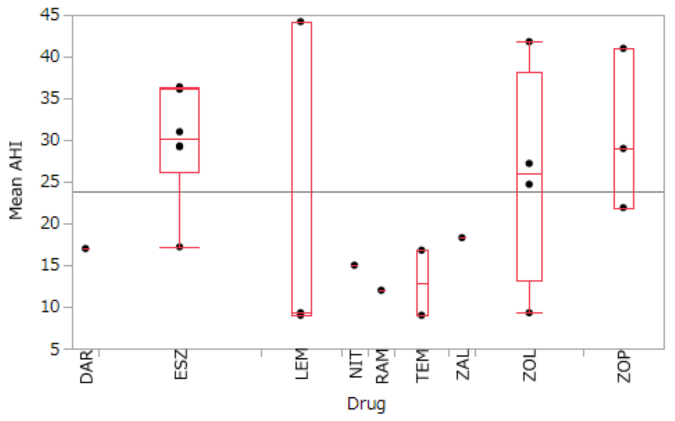	Chi-squared with ties = 8.43 (df = 8), $p = 0.39$
Mean BMI (kg/m^2)	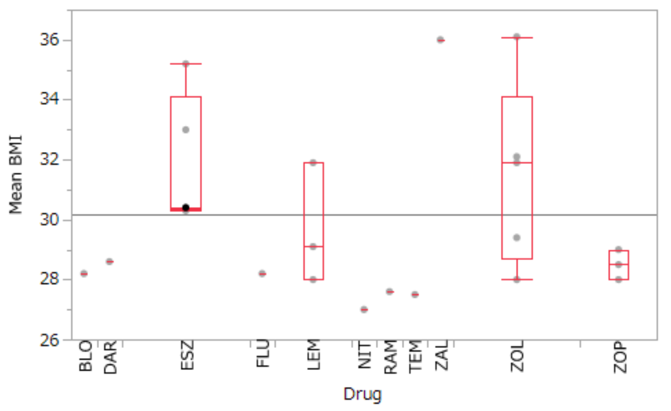	Chi-squared with ties = 14.92 (df = 10), $p = 0.13$
CPAP usage	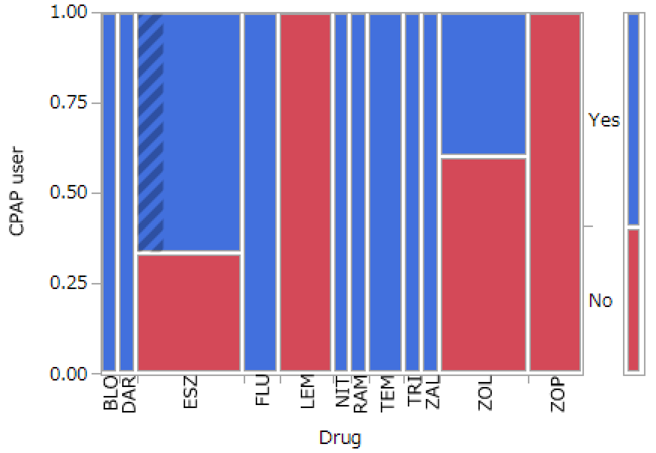	Chi-squared with ties = 16.51 (df = 11), $p = 0.13$

Study duration (d)	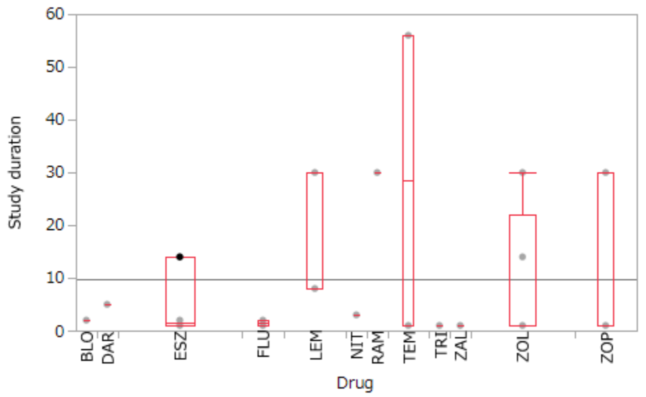	Chi-squared with ties = 7.51 (df = 11), p = 0.76
Sponsorship	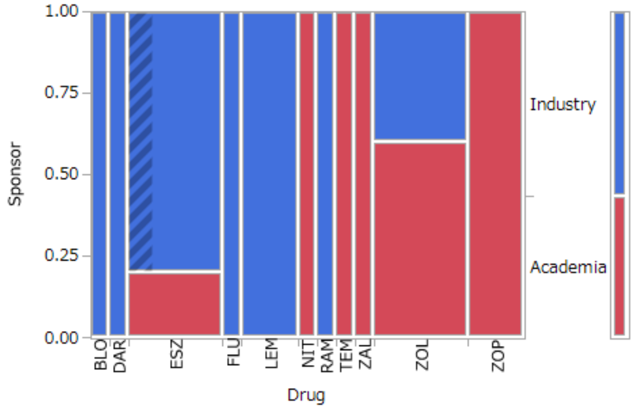	Chi-squared with ties = 14.86 (df = 10), p = 0.14
Crossover trial	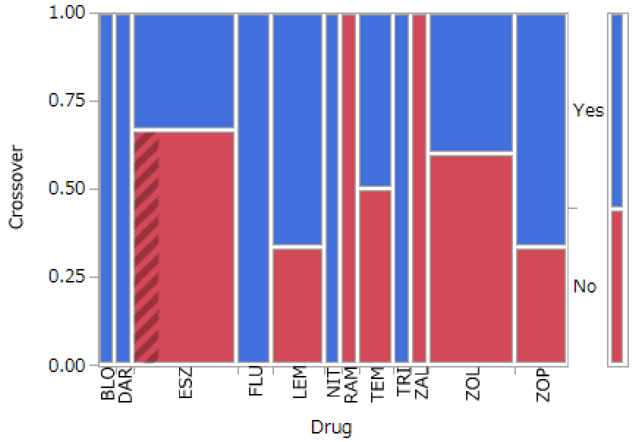	Chi-squared with ties = 9.32 (df = 11), p = 0.59

Washout period (d)	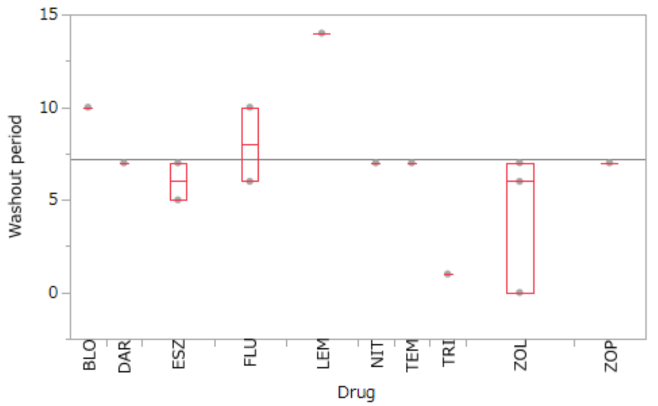	Chi-squared with ties = 10.6 (df = 9), p = 0.30
Total number of participants	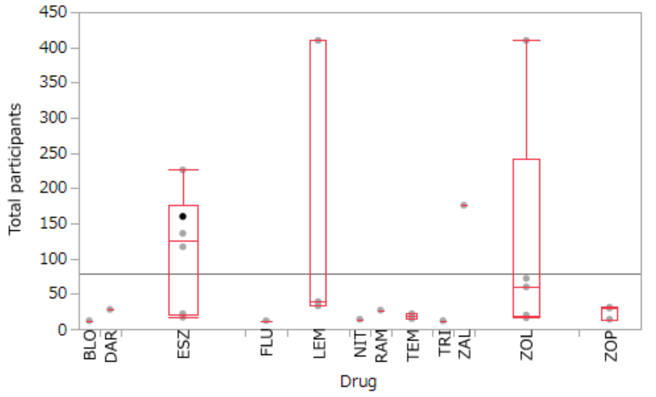	Chi-squared with ties = 16.7 (df = 11), p = 0.12
Publication year (y)	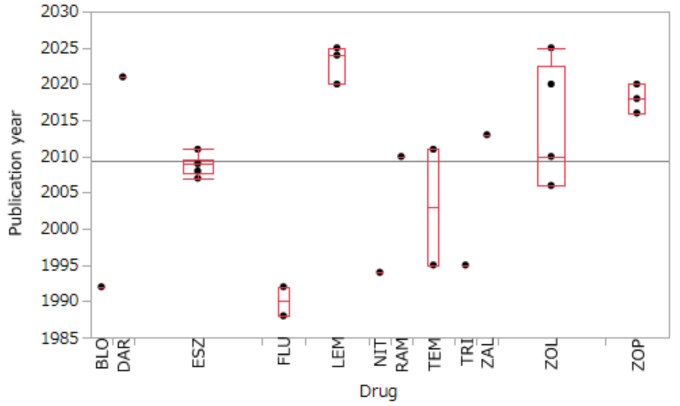	Chi-squared with ties = 19.59 (df = 11), p = 0.12

Overall risk of bias (high risk vs. others)	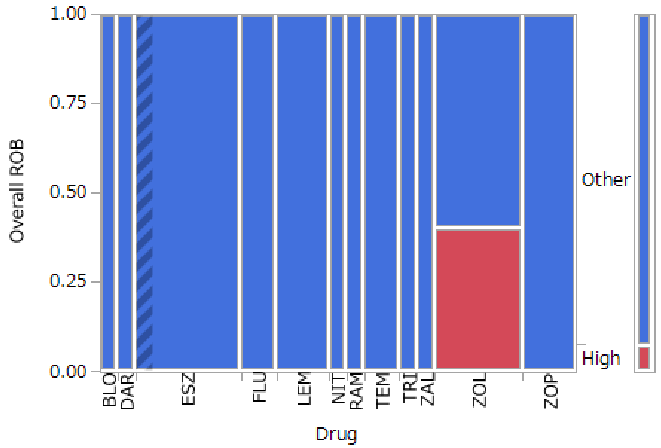	Chi-squared with ties = 9.50 (df = 11), p = 0.58

*When the *p*-value was <0.05, we considered that there was a difference in the data among the treatments.

AHI: apnea-hypopnea index, BMI: body mass index, CPAP: continuous positive airway pressure

eTable 4: Risk of bias summary for the trials included in the network meta-analysis

	Randomization process	Period and carryover effects†	Deviation from intended intervention	Missing outcome data	Measurement of the outcome	Selection of the reported result	Overall risk of bias
Berry 1995	Some concerns	Low risk	Some concerns	Low risk	Some concerns	Low risk	Some concerns
Berry 2006	Some concerns	Low risk	Some concerns	Low risk	Some concerns	Low risk	Some concerns
Boof 2021	Some concerns	Low risk	Some concerns	Low risk	Low risk	Low risk	Some concerns
Bradshaw 2006	Some concerns	NA	High risk	Low risk	Some concerns	Low risk	High risk
Camacho 1995	Some concerns	NA	Some concerns	Low risk	Some concerns	Low risk	Some concerns
Carter 2016	Some concerns	Low risk	Some concerns	Low risk	Some concerns	Low risk	Some concerns
Carter 2018	Some concerns	NA	Some concerns	Low risk	Some concerns	Low risk	Some concerns
Carter 2020	Some concerns	Low risk	Some concerns	Low risk	Some concerns	Low risk	Some concerns
Cheng 2020	Low risk	Low risk	Low risk	Low risk	Low risk	Low risk	Low risk
Cheng 2024	Low risk	Low risk	Low risk	Low risk	Low risk	Low risk	Low risk
Cirignotta 1988	Some concerns	Low risk	Some concerns	Low risk	Some concerns	Low risk	Some concerns
Cirignotta 1992	Some concerns	Low risk	Some concerns	Some concerns	Some concerns	Low risk	Some concerns
Eckert 2011	Some concerns	Low risk	Some concerns	Low risk	Some concerns	Low risk	Some concerns
George 2010	Some concerns	High risk	Some concerns	Low risk	Some concerns	Low risk	High risk
Gooneratne 2010	Some concerns	NA	Some concerns	Low risk	Some concerns	Low risk	Some concerns
Höijer 1994	Some concerns	Low risk	Some concerns	Some concerns	Some concerns	Low risk	Some concerns
Kushida 2025	Low risk	NA	Low risk	Low risk	Low risk	Some concerns‡	Some concerns
Lettieri 2008	Low risk	NA	Low risk	Low risk	Low risk	Low risk	Low risk
Lettieri 2009 (AIM)	Low risk	NA	Low risk	Low risk	Low risk	Low risk	Low risk
Lettieri 2009 (CHE)	Low risk	NA	Low risk	Low risk	Low risk	Low risk	Low risk
Messineo 2020	Some concerns	Low risk	Some concerns	Low risk	Some concerns	Low risk	Some concerns
Park 2013	Some concerns	NA	Low risk	Low risk	Low risk	Low risk	Some concerns
Rosenberg 2007	Some concerns	Low risk	Some concerns	Low risk	Some concerns	Low risk	Some concerns
Shah 2009	Some concerns	NA	Low risk	Low risk	Low risk	Low risk	Some concerns
Wang 2011	Low risk	Low risk	Low risk	Low risk	Low risk	Low risk	Low risk

Version 2 of the Cochrane risk-of-bias tool for randomized trials (https://www.riskofbias.info/)

AIM: Annals of Internal Medicine, CHE: Chest, NA: not applicable,

†The information is for the crossover study.

‡ This was a post hoc study. However, there were no significant differences regarding demographic and baseline characteristics among all treatment arms.

Appendix S1. Total sleep time

15 studies, 1084 participants

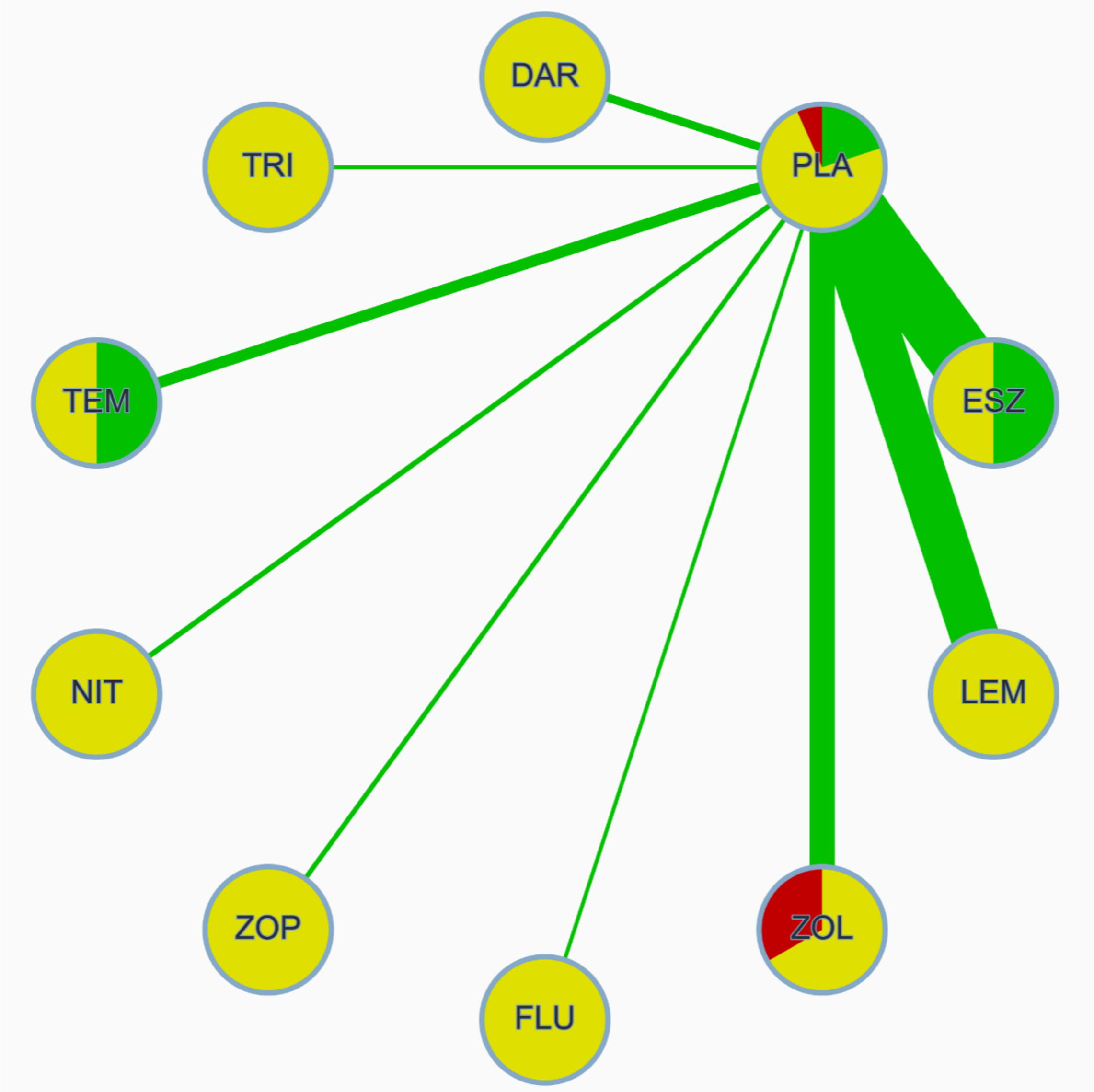

Node size by Equal size

Node color by risk of bias

Green: low overall risk of bias

Yellow: moderate overall risk of bias

Red: high overall risk of bias

Edge width by sample size

Edge color by average indirectness

Green: no indirectness

DAR: daridorexant, ESZ: eszopiclone, FLU: flurazepam, LEM: lemborexant, NIT: nitrazepam, PLA: placebo, RAM: ramelteon, TEM: temazepam, TRI: triazolam, ZO): zolpidem, ZOP: zopiclone

League table (SMD with 95% confidence interval)

DAR	0.145 (-0.528, 0.818)	-0.307 (-1.407, 0.793)	-0.213 (-0.936, 0.510)	0.353 (-0.641, 1.347)	**1.011 (0.209, 1.812)**	0.589 (-0.458, 1.635)	0.281 (-0.440, 1.001)	0.736 (-0.246, 1.718)	**0.814 (0.187, 1.440)**
	ESZ	-0.452 (-1.389, 0.486)	-0.358 (-0.797, 0.080)	0.208 (-0.602, 1.018)	**0.866 (0.307, 1.424)**	0.444 (-0.430, 1.317)	0.136 (-0.298, 0.570)	0.591 (-0.205, 1.387)	**0.668 (0.421, 0.916)**
		FLU	0.094 (-0.880, 1.067)	0.660 (-0.529, 1.848)	**1.317 (0.284, 2.351)**	0.895 (-0.337, 2.128)	0.588 (-0.384, 1.560)	1.043 (-0.136, 2.222)	**1.120 (0.216, 2.025)**
			LEM	0.566 (-0.286, 1.418)	**1.224 (0.606, 1.841)**	0.802 (-0.111, 1.714)	0.494 (-0.014, 1.002)	**0.949 (0.111, 1.788)**	**1.027 (0.665, 1.388)**
				NIT	0.658 (-0.262, 1.577)	0.236 (-0.903, 1.375)	-0.072 (-0.922, 0.778)	0.383 (-0.697, 1.464)	0.460 (-0.311, 1.232)
					TEM	-0.422 (-1.398, 0.554)	**-0.730 (-1.344, -0.116)**	-0.275 (-1.182, 0.632)	-0.197 (-0.698, 0.303)
						TRI	-0.308 (-1.218, 0.603)	0.148 (-0.981, 1.276)	0.225 (-0.613, 1.063)
							ZOL	0.455 (-0.381, 1.291)	**0.533 (0.176, 0.889)**
								ZOP	0.077 (-0.679, 0.834)
									PLA

Global heterogeneity

As previously suggested (Huhn 2019), the common τ^2 was compared to the empirical distributions of heterogeneity found in the meta-analyses of pharmacological treatments for mental health outcomes, with a median of the τ^2 distribution of 0.049 and an inter-quartile range of 0.010 to 0.242 (Rhodes 2015), and the heterogeneity was considered low when the estimated τ^2 was below the 25% quartile, moderate when between 25% and 50% of the quartile, and high when above the 50% quartile.

Huhn M, et al. Lancet 2019;394(10202):939-51

Rhodes KM, et al. J Clin Epidemiol 2015;68(1):52-60

Between study variance (τ^2): 0.015 (heterogeneity assessment: low to moderate)

Random-effects design-by-treatment interaction model

na

Local heterogeneity (I^2) and incoherence (SIDE test)

$I^2 \geq 50\%$ was considered as considerable heterogeneity. As a general rule, there are "no concerns" if the p-value is >0.10, independent of the position of the 95% CIs with respect to the range of equivalence, because the evidence for incoherence is weak ($p > 0.10$).

Nikolakopoulou A, et al., PLOS Medicine 2020 17 1-19, Papakonstantinou T, et al., Campbell Systematic Reviews 2020 16 e1080

	NMA SMD	Direct SMD	I^2	Indirect SMD	P value (SIDE test)
ESZ vs PLA		0.668 (0.421, 0.916)	0.0%		
TEM vs PLA		-0.197 (-0.698, 0.303)	0.0%		
ZOP vs PLA		0.077 (-0.679, 0.834)	**61.3%**		

Funnel plot (only placebo-controlled trials)

As none of the comparisons included 10 or more studies, the assessment of publication bias was not performed.

Meta-regression analysis (the placebo was the control)

Potential moderators	Median β (SE)
Proportion of males	41.855 (-263.120, 81.142)
Mean age (years)	35.856 (-71.070, 169.205)
Mean AHI (events/h)	221.933 (-0.314, 471.452)
Mean BMI (kg/m^2)	-7.814 (-176.026, 289.162)
CPAP usage	67.417 (-43.069, 96.284)
Study duration (d)	-0.032 (-98.094, 405.684)
Sponsorship	-35.779 (-91.792, 84.721)
Crossover trial	-232.937 (-497.066, 52.875)
Washout period (d)	-58.087 (-363.674, 133.585)
Total number of participants	-107.563 (-387.567, 97.307)
Publication year	0.402 (-290.519, 286.686)
Overall risk of bias	40.787 (-152.810, 250.658)

CINeMA confidence rating

CINeMA is a web application that simplifies the evaluation of confidence in the findings from a network meta-analysis. CINeMA is based on a methodological framework described in the following articles, which consider the following six domains: within-study bias, reporting bias, indirectness, imprecision, heterogeneity, and incoherence. CINeMA grades the confidence in the results of each treatment comparison as high, moderate, low, or very low. If the comparison had only indirect evidence, the comparison was downgraded one level.

Nikolakopoulou A, et al., PLOS Medicine 2020 17 1-19, Papakonstantinou T, et al., Campbell Systematic Reviews 2020 16 e1080

(1) Within-study bias: Risk of bias in RCTs for the main outcomes was assessed independently using the Cochrane risk-of-bias tool for randomized trials (RoB 2). In all studies, the

overall risk of bias was evaluated as “low risk.” Selected rule: Average

(2) Reporting bias: Comparison-adjusted funnel plots with less than 10 studies are not meaningful. Therefore, all comparisons were “Suspected.”

(3) Indirectness: No indirectness was assumed. Selected rule: Average

(4) Imprecision: For placebo comparisons the clinically meaningful threshold was set at a odds ratio of higher or lower than 1. For placebo comparisons the clinically meaningful threshold was set at a standardized mean difference of higher or lower than 0. For comparisons of two active treatments the clinically meaningful threshold was set at standardized mean differences of -0.1 and 0.1 for continuous outcomes and at a risk ratio of 0.8 and 1.25

for dichotomous outcomes.

(5) Heterogeneity: We used recommendations automatically provided by CINeMA.

(6) Incoherence: We used recommendations automatically provided by CINeMA.

Confidence rating: To counterweight the heavy impact of the risk of bias evaluation on the CINeMA evaluation we decided to downgrade by half a point for ‘some concerns’ and by one point for ‘major concerns.’ This allowed us to produce a clinically informative open range of judgements instead of a less helpful series of very-low confidence ratings, flattened down by the hypertrophic influence of the risk of bias evaluation.

Papola D, et al., Br J Psychiatry. 2022 Sep;221(3):507-519.

Comparison	Number of studies	Within-study bias	Reporting bias	Indirectness	Imprecision	Heterogeneity	Incoherence	Confidence rating
DAR vs PLA	1	Some concerns	Some concerns	No concerns	No concerns	Major concerns	Major concerns	Very low
ESZ vs PLA	4	No concerns	Some concerns	No concerns	No concerns	No concerns	Major concerns	Low
FLU vs PLA	1	Some concerns	Some concerns	No concerns	No concerns	Major concerns	Major concerns	Very low
LEM vs PLA	1	Some concerns	Some concerns	No concerns	No concerns	No concerns	Major concerns	Low
NIT vs PLA	1	Some concerns	Some concerns	No concerns	Major concerns	No concerns	Major concerns	Very low
TEM vs PLA	2	No concerns	Some concerns	No concerns	Major concerns	No concerns	Major concerns	Very low
TRI vs PLA	1	Some concerns	Some concerns	No concerns	Major concerns	No concerns	Major concerns	Very low
ZOL vs PLA	3	Some concerns	Some concerns	No concerns	No concerns	Major concerns	Major concerns	Very low
ZOP vs PLA	1	Some concerns	Some concerns	No concerns	Major concerns	No concerns	Major concerns	Very low
DAR vs ESZ	0	Some concerns	Some concerns	No concerns	Major concerns	No concerns	Major concerns	Very low
DAR vs FLU	0	Some concerns	Some concerns	No concerns	Major concerns	No concerns	Major concerns	Very low
DAR vs LEM	0	Some concerns	Some concerns	No concerns	Major concerns	No concerns	Major concerns	Very low
DAR vs NIT	0	Some concerns	Some concerns	No concerns	Major concerns	No concerns	Major concerns	Very low
DAR vs TEM	0	Some concerns	Some concerns	No concerns	No concerns	Major concerns	Major concerns	Very low
DAR vs TRI	0	Some concerns	Some concerns	No concerns	Major concerns	No concerns	Major concerns	Very low
DAR vs ZOL	0	Some concerns	Some concerns	No concerns	Major concerns	No concerns	Major concerns	Very low
DAR vs ZOP	0	Some concerns	Some concerns	No concerns	Major concerns	No concerns	Major concerns	Very low
ESZ vs FLU	0	Some concerns	Some concerns	No concerns	Major concerns	No concerns	Major concerns	Very low

ESZ vs LEM	0	Some concerns	Some concerns	No concerns	Major concerns	No concerns	Major concerns	Very low
ESZ vs NIT	0	Some concerns	Some concerns	No concerns	Major concerns	No concerns	Major concerns	Very low
ESZ vs TEM	0	No concerns	Some concerns	No concerns	No concerns	No concerns	Major concerns	Very low
ESZ vs TRI	0	Some concerns	Some concerns	No concerns	Major concerns	No concerns	Major concerns	Very low
ESZ vs ZOL	0	Some concerns	Some concerns	No concerns	Major concerns	No concerns	Major concerns	Very low
ESZ vs ZOP	0	Some concerns	Some concerns	No concerns	Major concerns	No concerns	Major concerns	Very low
FLU vs LEM	0	Some concerns	Some concerns	No concerns	Major concerns	No concerns	Major concerns	Very low
FLU vs NIT	0	Some concerns	Some concerns	No concerns	Major concerns	No concerns	Major concerns	Very low
FLU vs TEM	0	Some concerns	Some concerns	No concerns	No concerns	Major concerns	Major concerns	Very low
FLU vs TRI	0	Some concerns	Some concerns	No concerns	Major concerns	No concerns	Major concerns	Very low
FLU vs ZOL	0	Some concerns	Some concerns	No concerns	Major concerns	No concerns	Major concerns	Very low
FLU vs ZOP	0	Some concerns	Some concerns	No concerns	Major concerns	No concerns	Major concerns	Very low
LEM vs NIT	0	Some concerns	Some concerns	No concerns	Major concerns	No concerns	Major concerns	Very low
LEM vs TEM	0	Some concerns	Some concerns	No concerns	No concerns	No concerns	Major concerns	Very low
LEM vs TRI	0	Some concerns	Some concerns	No concerns	Major concerns	No concerns	Major concerns	Very low
LEM vs ZOL	0	Some concerns	Some concerns	No concerns	Major concerns	No concerns	Major concerns	Very low
LEM vs ZOP	0	Some concerns	Some concerns	No concerns	No concerns	Major concerns	Major concerns	Very low
NIT vs TEM	0	Some concerns	Some concerns	No concerns	Major concerns	No concerns	Major concerns	Very low
NIT vs TRI	0	Some concerns	Some concerns	No concerns	Major concerns	No concerns	Major concerns	Very low
NIT vs ZOL	0	Some concerns	Some concerns	No concerns	Major concerns	No concerns	Major concerns	Very low
NIT vs ZOP	0	Some concerns	Some concerns	No concerns	Major concerns	No concerns	Major concerns	Very low
TEM vs TRI	0	Some concerns	Some concerns	No concerns	Major concerns	No concerns	Major concerns	Very low
TEM vs ZOL	0	Some concerns	Some concerns	No concerns	No concerns	Major concerns	Major concerns	Very low
TEM vs ZOP	0	Some concerns	Some concerns	No concerns	Major concerns	No concerns	Major concerns	Very low
TRI vs ZOL	0	Some concerns	Some concerns	No concerns	Major concerns	No concerns	Major concerns	Very low
TRI vs ZOP	0	Some concerns	Some concerns	No concerns	Major concerns	No concerns	Major concerns	Very low
ZOL vs ZOP	0	Some concerns	Some concerns	No concerns	Major concerns	No concerns	Major concerns	Very low

Sensitivity analysis excluding CPAP titration studies

League table (SMD with 95% confidence interval)

DAR	0.046 (-0.926, 1.018)	-0.307 (-1.578, 0.964)	-0.213 (-1.177, 0.750)	0.353 (-0.827, 1.533)	**1.016 (0.043, 1.990)**	0.589 (-0.636, 1.813)	0.313 (-0.578, 1.205)	0.736 (-0.434, 1.906)	**0.814 (0.042, 1.585)**
	ESZ	-0.353 (-1.523, 0.818)	-0.259 (-1.085, 0.567)	0.307 (-0.764, 1.378)	**0.971 (0.132, 1.809)**	0.543 (-0.577, 1.663)	0.267 (-0.474, 1.009)	0.690 (-0.370, 1.750)	**0.768 (0.176, 1.359)**
		FLU	0.094 (-1.070, 1.257)	0.660 (-0.689, 2.008)	**1.323 (0.151, 2.495)**	0.895 (-0.492, 2.283)	0.620 (-0.484, 1.725)	1.043 (-0.297, 2.383)	**1.120 (0.110, 2.130)**

			LEM	0.566 (-0.498, 1.630)	**1.230 (0.401, 2.058)**	0.802 (-0.311, 1.915)	0.526 (-0.204, 1.257)	0.949 (-0.104, 2.002)	**1.027 (0.449, 1.604)**
				NIT	0.664 (-0.409, 1.736)	0.236 (-1.069, 1.540)	-0.040 (-1.038, 0.959)	0.383 (-0.871, 1.637)	0.460 (-0.433, 1.354)
					TEM	-0.428 (-1.550, 0.694)	-0.703 (-1.447, 0.041)	-0.280 (-1.342, 0.782)	-0.203 (-0.797, 0.391)
						TRI	-0.275 (-1.326, 0.776)	0.148 (-1.149, 1.444)	0.225 (-0.726, 1.176)
							ZOL	0.423 (-0.564, 1.410)	**0.500 (0.053, 0.947)**
								ZOP	0.077 (-0.803, 0.958)
									PLA

Global heterogeneity

Between study variance (τ^2): 0.068 (heterogeneity assessment: moderate to high)

Random-effects design-by-treatment interaction model

na

Subgroup analysis including studies involved only non-CPAP users

League table (SMD with 95% confidence interval)

ESZ	-0.275 (-0.814, 0.265)	0.004 (-0.589, 0.597)	0.674 (-0.181, 1.530)	**0.752 (0.286, 1.218)**
	LEM	0.279 (-0.177, 0.735)	**0.949 (0.182, 1.716)**	**1.027 (0.755, 1.298)**
		ZOL	0.670 (-0.135, 1.476)	**0.748 (0.381, 1.114)**
			ZOP	0.077 (-0.640, 0.795)
				PLA

Global heterogeneity

Between study variance (τ^2): 0.000 (heterogeneity assessment: low)

Random-effects design-by-treatment interaction model

na

Subgroup analysis including only CPAP titration

	Direct SMD	I^2
ESZ vs PLA	**0.632 (0.408, 0.855)**	0.0%

Appendix S2. Rapid eye movement sleep time

10 studies, 799 participants

League table (SMD with 95% confidence interval)

DAR	0.098 (-0.776, 0.971)	-0.535 (-1.142, 0.073)	0.909 (-0.020, 1.837)	0.759 (-0.083, 1.600)	0.200 (-0.400, 0.800)	0.300 (-0.371, 0.971)	0.247 (-0.309, 0.804)	
	ESZ	-0.632 (-1.348, 0.084)	0.811 (-0.192, 1.814)	0.661 (-0.262, 1.584)	0.102 (-0.608, 0.812)	0.202 (-0.568, 0.973)	0.150 (-0.524, 0.823)	
		LEM	**1.443 (0.661, 2.225)**	**1.293 (0.617, 1.969)**	**0.734 (0.512, 0.957)**	**0.835 (0.388, 1.281)**	**0.782 (0.539, 1.025)**	
			NIT	-0.150 (-1.125, 0.825)	-0.709 (-1.486, 0.067)	-0.609 (-1.441, 0.224)	-0.661 (-1.405, 0.082)	
				TEM	-0.559 (-1.228, 0.110)	-0.459 (-1.192, 0.275)	-0.511 (-1.142, 0.120)	
					ZOL	0.100 (-0.336, 0.536)	0.048 (-0.176, 0.272)	
						ZOP	-0.053 (-0.427, 0.322)	
							PLA	

SUCRA

DAR	71.7%
ESZ	55.0%
LEM	97.8%
NIT	15.9%
TEM	19.6%
ZOL	54.8%
ZOP	22.2%
PLA	27.4%

Global heterogeneity

Between study variance (τ^2): 0.000 (heterogeneity assessment: low)

Random-effects design-by-treatment interaction model

χ^2 statistic: 0.000 (1 degrees of freedom), P value: 0.995

Local heterogeneity (I^2) and incoherence (SIDE test)

	NMA SMD	Direct SMD	I^2	Indirect SMD	P value (SIDE test)
LEM vs ZOL	0.734 (0.512, 0.957)	0.734 (0.499, 0.969)		0.736 (0.033, 1.439)	0.996
LEM vs PLA	0.782 (0.539, 1.025)	0.782 (0.516, 1.048)		0.780 (0.186, 1.374)	0.996
ZOL vs PLA		0.048 (-0.176, 0.272)	0.0%		
ZOP vs PLA		-0.053 (-0.427, 0.322)	0.0%		

CINeMA confidence rating

Comparison	Number of studies	Within-study bias	Reporting bias	Indirectness	Imprecision	Heterogeneity	Incoherence	Confidence rating

DAR vs PLA	1	Some concerns	Some concerns	No concerns	Major concerns	No concerns	No concerns	Low
ESZ vs PLA	1	Some concerns	Some concerns	No concerns	Major concerns	No concerns	No concerns	Low
LEM vs ZOL	1	Some concerns	Some concerns	No concerns	No concerns	No concerns	No concerns	Moderate
LEM vs PLA	1	Some concerns	Some concerns	No concerns	No concerns	No concerns	No concerns	Moderate
NIT vs PLA	1	Some concerns	Some concerns	No concerns	Major concerns	No concerns	No concerns	Low
TEM vs PLA	1	No concerns	Some concerns	No concerns	Major concerns	No concerns	No concerns	Low
ZOL vs PLA	3	Some concerns	Some concerns	No concerns	Major concerns	No concerns	No concerns	Very low
ZOP vs PLA	3	Some concerns	Some concerns	No concerns	Major concerns	No concerns	No concerns	Very low
DAR vs ESZ	0	Some concerns	Some concerns	No concerns	Major concerns	No concerns	No concerns	Very low
DAR vs LEM	0	Some concerns	Some concerns	No concerns	Major concerns	No concerns	No concerns	Very low
DAR vs NIT	0	Some concerns	Some concerns	No concerns	Major concerns	No concerns	No concerns	Very low
DAR vs TEM	0	Some concerns	Some concerns	No concerns	Major concerns	No concerns	No concerns	Very low
DAR vs ZOL	0	Some concerns	Some concerns	No concerns	Major concerns	No concerns	No concerns	Very low
DAR vs ZOP	0	Some concerns	Some concerns	No concerns	Major concerns	No concerns	No concerns	Very low
ESZ vs LEM	0	Some concerns	Some concerns	No concerns	Major concerns	No concerns	No concerns	Very low
ESZ vs NIT	0	Some concerns	Some concerns	No concerns	Major concerns	No concerns	No concerns	Very low
ESZ vs TEM	0	Some concerns	Some concerns	No concerns	Major concerns	No concerns	No concerns	Very low
ESZ vs ZOL	0	Some concerns	Some concerns	No concerns	Major concerns	No concerns	No concerns	Very low
ESZ vs ZOP	0	Some concerns	Some concerns	No concerns	Major concerns	No concerns	No concerns	Very low
LEM vs NIT	0	Some concerns	Some concerns	No concerns	No concerns	No concerns	No concerns	Very low
LEM vs TEM	0	Some concerns	Some concerns	No concerns	No concerns	No concerns	No concerns	Very low
LEM vs ZOP	0	Some concerns	Some concerns	No concerns	No concerns	No concerns	No concerns	Very low
NIT vs TEM	0	Some concerns	Some concerns	No concerns	Major concerns	No concerns	No concerns	Very low
NIT vs ZOL	0	Some concerns	Some concerns	No concerns	Major concerns	No concerns	No concerns	Very low
NIT vs ZOP	0	Some concerns	Some concerns	No concerns	Major concerns	No concerns	No concerns	Very low
TEM vs ZOL	0	Some concerns	Some concerns	No concerns	Major concerns	No concerns	No concerns	Very low
TEM vs ZOP	0	Some concerns	Some concerns	No concerns	Major concerns	No concerns	No concerns	Very low
ZOL vs ZOP	0	Some concerns	Some concerns	No concerns	Major concerns	No concerns	No concerns	Very low

Sensitivity analysis excluding CPAP titration studies

League table (SMD with 95% confidence interval)

DAR	0.098 (-0.776, 0.971)	-0.535 (-1.142, 0.073)	0.909 (-0.020, 1.837)	0.759 (-0.083, 1.600)	0.200 (-0.400, 0.800)	0.300 (-0.371, 0.971)	0.247 (-0.309, 0.804)
	ESZ	-0.632 (-1.348, 0.084)	0.811 (-0.192, 1.814)	0.661 (-0.262, 1.584)	0.102 (-0.608, 0.812)	0.202 (-0.568, 0.973)	0.150 (-0.524, 0.823)

		LEM	**1.443 (0.661, 2.225)**	**1.293 (0.617, 1.969)**	**0.734 (0.512, 0.957)**	**0.835 (0.388, 1.281)**	**0.782 (0.539, 1.025)**
			NIT	-0.150 (-1.125, 0.825)	-0.709 (-1.486, 0.067)	-0.609 (-1.441, 0.224)	-0.661 (-1.405, 0.082)
				TEM	-0.559 (-1.228, 0.110)	-0.459 (-1.192, 0.275)	-0.511 (-1.142, 0.120)
					ZOL	0.100 (-0.336, 0.536)	0.048 (-0.176, 0.272)
						ZOP	-0.053 (-0.427, 0.322)
							PLA

Global heterogeneity

Between study variance (τ^2): 0.000 (heterogeneity assessment: low)

Random-effects design-by-treatment interaction model

χ^2 statistic: 0.137 (1 degrees of freedom), P value: 0.711

Local heterogeneity (I^2) and incoherence (SIDE test)

	NMA SMD	Direct SMD	I^2	Indirect SMD	P value (SIDE test)
LEM vs ZOL	0.734 (0.512, 0.957)	0.734 (0.499, 0.969)		0.736 (0.033, 1.439)	0.996
LEM vs PLA	0.782 (0.539, 1.025)	0.782 (0.516, 1.048)		0.780 (0.186, 1.374)	0.996
ZOL vs PLA		0.048 (-0.176, 0.272)	0.0%		
ZOP vs PLA		-0.053 (-0.427, 0.322)	0.0%		

Subgroup analysis including studies involved only non-CPAP users

League table (SMD with 95% confidence interval)

ESZ	-0.632 (-1.348, 0.084)	0.102 (-0.608, 0.812)	0.202 (-0.568, 0.973)	0.150 (-0.524, 0.823)
	LEM	**0.734 (0.512, 0.957)**	**0.835 (0.388, 1.281)**	**0.782 (0.539, 1.025)**
		ZOL	0.100 (-0.336, 0.536)	0.048 (-0.176, 0.272)
			ZOP	-0.053 (-0.427, 0.322)
				PLA

Global heterogeneity

Between study variance (τ^2): 0.000 (heterogeneity assessment: low)

Random-effects design-by-treatment interaction model

na

Local heterogeneity (I^2) and incoherence (SIDE test)

	NMA SMD	Direct SMD	I^2	Indirect SMD	P value (SIDE test)
LEM vs ZOL	0.734 (0.512, 0.957)	0.734 (0.499, 0.969)		0.736 (0.033, 1.439)	0.996
LEM vs PLA	0.782 (0.539, 1.025)	0.782 (0.516, 1.048)		0.780 (0.186, 1.374)	0.996
ZOL vs PLA		0.048 (-0.176, 0.272)	0.0%		
ZOP vs PLA		-0.053 (-0.427, 0.322)	0.0%		

Appendix S3. Latency to persistent sleep

10 studies, 896 participants

League table (SMD with 95% confidence interval) SUCRA

DAR	-0.115 (-0.715, 0.484)	-0.435 (-1.414, 0.543)	0.083 (-0.526, 0.691)	0.464 (-0.628, 1.556)	-0.453 (-1.290, 0.384)	-0.519 (-1.362, 0.324)	-0.830 (-1.814, 0.155)	-0.477 (-1.040, 0.086)		DAR	73.9%
	ESZ	-0.320 (-1.147, 0.506)	0.198 (-0.112, 0.508)	0.580 (-0.378, 1.538)	-0.338 (-0.991, 0.316)	-0.404 (-1.065, 0.258)	-0.714 (-1.548, 0.119)	**-0.362 (-0.568, -0.155)**		ESZ	72.5%
		FLU	0.518 (-0.315, 1.351)	0.900 (-0.331, 2.131)	-0.018 (-1.030, 0.995)	-0.083 (-1.101, 0.934)	-0.394 (-1.531, 0.743)	-0.042 (-0.842, 0.759)		FLU	60.6%
			LEM	0.382 (-0.582, 1.346)	-0.536 (-1.197, 0.126)	-0.602 (-1.271, 0.068)	**-0.912 (-1.752, -0.072)**	**-0.560 (-0.792, -0.328)**		LEM	82.1%
				RAM	-0.917 (-2.040, 0.205)	-0.983 (-2.110, 0.144)	**-1.294 (-2.530, -0.058)**	**-0.942 (-1.877, -0.006)**		RAM	92.5%
					TEM	-0.066 (-0.948, 0.816)	-0.377 (-1.395, 0.641)	-0.024 (-0.644, 0.596)		TEM	59.8%
						ZOL	-0.311 (-1.334, 0.712)	0.042 (-0.586, 0.670)		ZOL	15.1%
							ZOP	0.353 (-0.455, 1.160)		ZOP	9.2%
								PLA		PLA	25.3%

Global heterogeneity

Between study variance (τ^2): 0.000 (heterogeneity assessment: low)

Random-effects design-by-treatment interaction model

na

Local heterogeneity (I^2) and incoherence (SIDE test)

	NMA SMD	Direct SMD	I^2	Indirect SMD	P value (SIDE test)
ESZ vs PLA		-0.362 (-0.568, -0.155)	0.0%		

CINeMA confidence rating

Comparison	Number of studies	Within-study bias	Reporting bias	Indirectness	Imprecision	Heterogeneity	Incoherence	Confidence rating
DAR vs PLA	1	Some concerns	Some concerns	No concerns	Major concerns	No concerns	Major concerns	Very low
ESZ vs PLA	3	No concerns	Some concerns	No concerns	No concerns	Major concerns	Major concerns	Very low
FLU vs PLA	1	Some concerns	Some concerns	No concerns	Major concerns	No concerns	Major concerns	Very low
LEM vs PLA	1	Some concerns	Some concerns	No concerns	No concerns	Major concerns	Major concerns	Very low
RAM vs PLA	1	Some concerns	Some concerns	No concerns	No concerns	Major concerns	Major concerns	Very low
TEM vs PLA	1	No concerns	Some concerns	No concerns	Major concerns	No concerns	Major concerns	Very low
ZOL vs PLA	1	Some concerns	Some concerns	No concerns	Major concerns	No concerns	Major concerns	Very low
ZOP vs PLA	1	Some concerns	Some concerns	No concerns	Major concerns	No concerns	Major concerns	Very low
DAR vs ESZ	0	Some concerns	Some concerns	No concerns	Major concerns	No concerns	Major concerns	Very low
DAR vs FLU	0	Some concerns	Some concerns	No concerns	Major concerns	No concerns	Major concerns	Very low
DAR vs LEM	0	Some concerns	Some concerns	No concerns	Major concerns	No concerns	Major concerns	Very low
DAR vs RAM	0	Some concerns	Some concerns	No concerns	Major concerns	No concerns	Major concerns	Very low
DAR vs TEM	0	Some concerns	Some concerns	No concerns	Major concerns	No concerns	Major concerns	Very low
DAR vs ZOL	0	Some concerns	Some concerns	No concerns	Major concerns	No concerns	Major concerns	Very low
DAR vs ZOP	0	Some concerns	Some concerns	No concerns	Major concerns	No concerns	Major concerns	Very low
ESZ vs FLU	0	Some concerns	Some concerns	No concerns	Major concerns	No concerns	Major concerns	Very low
ESZ vs LEM	0	Some concerns	Some concerns	No concerns	Major concerns	No concerns	Major concerns	Very low
ESZ vs RAM	0	Some concerns	Some concerns	No concerns	Major concerns	No concerns	Major concerns	Very low
ESZ vs TEM	0	No concerns	Some concerns	No concerns	Major concerns	No concerns	Major concerns	Very low
ESZ vs ZOL	0	Some concerns	Some concerns	No concerns	Major concerns	No concerns	Major concerns	Very low
ESZ vs ZOP	0	Some concerns	Some concerns	No concerns	Major concerns	No concerns	Major concerns	Very low
FLU vs LEM	0	Some concerns	Some concerns	No concerns	Major concerns	No concerns	Major concerns	Very low
FLU vs RAM	0	Some concerns	Some concerns	No concerns	Major concerns	No concerns	Major concerns	Very low
FLU vs TEM	0	Some concerns	Some concerns	No concerns	Major concerns	No concerns	Major concerns	Very low
FLU vs ZOL	0	Some concerns	Some concerns	No concerns	Major concerns	No concerns	Major concerns	Very low
FLU vs ZOP	0	Some concerns	Some concerns	No concerns	Major concerns	No concerns	Major concerns	Very low
LEM vs RAM	0	Some concerns	Some concerns	No concerns	Major concerns	No concerns	Major concerns	Very low
LEM vs TEM	0	Some concerns	Some concerns	No concerns	Major concerns	No concerns	Major concerns	Very low
LEM vs ZOL	0	Some concerns	Some concerns	No concerns	Major concerns	No concerns	Major concerns	Very low
LEM vs ZOP	0	Some concerns	Some concerns	No concerns	No concerns	Major concerns	Major concerns	Very low
RAM vs TEM	0	Some concerns	Some concerns	No concerns	Major concerns	No concerns	Major concerns	Very low
RAM vs ZOL	0	Some concerns	Some concerns	No concerns	Major concerns	No concerns	Major concerns	Very low
RAM vs ZOP	0	Some concerns	Some concerns	No concerns	No concerns	Major concerns	Major concerns	Very low

TEM vs ZOL	0	Some concerns	Some concerns	No concerns	Major concerns	No concerns	Major concerns	Very low
TEM vs ZOP	0	Some concerns	Some concerns	No concerns	Major concerns	No concerns	Major concerns	Very low
ZOL vs ZOP	0	Some concerns	Some concerns	No concerns	Major concerns	No concerns	Major concerns	Very low

Sensitivity analysis excluding CPAP titration studies

League table (SMD with 95% confidence interval)

DAR	-0.265 (-1.088, 0.557)	-0.435 (-1.414, 0.543)	0.083 (-0.526, 0.691)	0.464 (-0.628, 1.556)	-0.453 (-1.290, 0.384)	-0.519 (-1.362, 0.324)	-0.830 (-1.814, 0.155)	-0.477 (-1.040, 0.086)
	ESZ	-0.170 (-1.170, 0.830)	0.348 (-0.295, 0.991)	0.730 (-0.382, 1.841)	-0.188 (-1.050, 0.675)	-0.254 (-1.122, 0.615)	-0.564 (-1.570, 0.442)	-0.212 (-0.812, 0.388)
		FLU	0.518 (-0.315, 1.351)	0.900 (-0.331, 2.131)	-0.018 (-1.030, 0.995)	-0.083 (-1.101, 0.934)	-0.394 (-1.531, 0.743)	-0.042 (-0.842, 0.759)
			LEM	0.382 (-0.582, 1.346)	-0.536 (-1.197, 0.126)	-0.602 (-1.271, 0.068)	**-0.912 (-1.752, -0.072)**	**-0.560 (-0.792, -0.328)**
				RAM	-0.917 (-2.040, 0.205)	-0.983 (-2.110, 0.144)	**-1.294 (-2.530, -0.058)**	**-0.942 (-1.877, -0.006)**
					TEM	-0.066 (-0.948, 0.816)	-0.377 (-1.395, 0.641)	-0.024 (-0.644, 0.596)
						ZOL	-0.311 (-1.334, 0.712)	0.042 (-0.586, 0.670)
							ZOP	0.353 (-0.455, 1.160)
								PLA

Global heterogeneity

Between study variance (τ^2): 0.000 (heterogeneity assessment: low)

Random-effects design-by-treatment interaction model

na

Local heterogeneity (I^2) and incoherence (SIDE test)

na

Subgroup analysis including studies involved only non-CPAP users

League table (SMD with 95% confidence interval)

ESZ	0.348 (-0.295, 0.991)	-0.254 (-1.122, 0.615)	-0.564 (-1.570, 0.442)	-0.212 (-0.812, 0.388)
	LEM	-0.602 (-1.271, 0.068)	**-0.912 (-1.752, -0.072)**	**-0.560 (-0.792, -0.328)**
		ZOL	-0.311 (-1.334, 0.712)	0.042 (-0.586, 0.670)
			ZOP	0.353 (-0.455, 1.160)
				PLA

Global heterogeneity

Between study variance (τ^2): 0.000 (heterogeneity assessment: low)

Random-effects design-by-treatment interaction model

na

Local heterogeneity (I^2) and incoherence (SIDE test)

na

Subgroup analysis including only CPAP titration

	Direct SMD	I^2
ESZ vs PLA	**-0.384 (-0.604, -0.164)**	0.0%

Appendix S4. Wake after sleep onset

10 studies, 921 participants

League table (SMD with 95% confidence interval)

DAR	-0.002 (-0.610, 0.606)	0.663 (-0.402, 1.728)	0.117 (-0.513, 0.746)	0.276 (-0.674, 1.226)	-0.003 (-0.679, 0.674)	-0.392 (-1.312, 0.527)	**-0.663 (-1.233, -0.092)**	
	ESZ	0.665 (-0.258, 1.588)	0.118 (-0.221, 0.458)	0.278 (-0.511, 1.066)	-0.001 (-0.421, 0.419)	-0.391 (-1.142, 0.360)	**-0.661 (-0.871, -0.450)**	
		FLU	-0.546 (-1.484, 0.391)	-0.388 (-1.565, 0.790)	-0.666 (-1.636, 0.304)	-1.056 (-2.208, 0.097)	**-1.326 (-2.225, -0.426)**	
			LEM	0.159 (-0.646, 0.964)	-0.119 (-0.570, 0.331)	-0.509 (-1.278, 0.259)	**-0.779 (-1.045, -0.513)**	
				TEM	-0.278 (-1.121, 0.564)	-0.668 (-1.715, 0.379)	**-0.938 (-1.698, -0.178)**	
					ZOL	-0.390 (-1.197, 0.418)	**-0.660 (-1.024, -0.296)**	
						ZOP	-0.270 (-0.991, 0.451)	
							PLA	

SUCRA

DAR	84.3%
ESZ	84.3%
FLU	94.1%
LEM	88.8%
TEM	92.3%
ZOL	82.3%
ZOP	55.3%
PLA	23.4%

Global heterogeneity

Between study variance (τ^2): 0.000 (heterogeneity assessment: low)

Random-effects design-by-treatment interaction model

na

Local heterogeneity (I^2) and incoherence (SIDE test)

	NMA SMD	Direct SMD	I^2	Indirect SMD	P value (SIDE test)
ESZ vs PLA		-0.661 (-0.871, -0.450)	0.0%		
ZOL vs PLA		-0.660 (-1.024, -0.296)	0.0%		

CINeMA confidence rating

Comparison	Number of studies	Within-study bias	Reporting bias	Indirectness	Imprecision	Heterogeneity	Incoherence	Confidence rating
DAR vs PLA	1	Some concerns	Some concerns	No concerns	No concerns	Major concerns	Major concerns	Very low
ESZ vs PLA	3	No concerns	Some concerns	No concerns	No concerns	No concerns	Major concerns	Low

FLU vs PLA	1	Some concerns	Some concerns	No concerns	No concerns	Major concerns	Major concerns	Very low
LEM vs PLA	1	Some concerns	Some concerns	No concerns	No concerns	No concerns	Major concerns	Low
TEM vs PLA	1	Some concerns	Some concerns	No concerns	No concerns	Major concerns	Major concerns	Very low
ZOL vs PLA	2	Major concerns	Some concerns	No concerns	No concerns	Major concerns	Major concerns	Very low
ZOP vs PLA	1	Some concerns	Some concerns	No concerns	Major concerns	No concerns	Major concerns	Very low
DAR vs ESZ	0	Some concerns	Some concerns	No concerns	Major concerns	No concerns	Major concerns	Very low
DAR vs FLU	0	Some concerns	Some concerns	No concerns	Major concerns	No concerns	Major concerns	Very low
DAR vs LEM	0	Some concerns	Some concerns	No concerns	Major concerns	No concerns	Major concerns	Very low
DAR vs TEM	0	Some concerns	Some concerns	No concerns	Major concerns	No concerns	Major concerns	Very low
DAR vs ZOL	0	Some concerns	Some concerns	No concerns	Major concerns	No concerns	Major concerns	Very low
DAR vs ZOP	0	Some concerns	Some concerns	No concerns	Major concerns	No concerns	Major concerns	Very low
ESZ vs FLU	0	Some concerns	Some concerns	No concerns	Major concerns	No concerns	Major concerns	Very low
ESZ vs LEM	0	Some concerns	Some concerns	No concerns	Major concerns	No concerns	Major concerns	Very low
ESZ vs TEM	0	Some concerns	Some concerns	No concerns	Major concerns	No concerns	Major concerns	Very low
ESZ vs ZOL	0	Some concerns	Some concerns	No concerns	Major concerns	No concerns	Major concerns	Very low
ESZ vs ZOP	0	Some concerns	Some concerns	No concerns	Major concerns	No concerns	Major concerns	Very low
FLU vs LEM	0	Some concerns	Some concerns	No concerns	Major concerns	No concerns	Major concerns	Very low
FLU vs TEM	0	Some concerns	Some concerns	No concerns	Major concerns	No concerns	Major concerns	Very low
FLU vs ZOL	0	Some concerns	Some concerns	No concerns	Major concerns	No concerns	Major concerns	Very low
FLU vs ZOP	0	Some concerns	Some concerns	No concerns	Major concerns	No concerns	Major concerns	Very low
LEM vs TEM	0	Some concerns	Some concerns	No concerns	Major concerns	No concerns	Major concerns	Very low
LEM vs ZOL	0	Some concerns	Some concerns	No concerns	Major concerns	No concerns	Major concerns	Very low
LEM vs ZOP	0	Some concerns	Some concerns	No concerns	Major concerns	No concerns	Major concerns	Very low
TEM vs ZOL	0	Some concerns	Some concerns	No concerns	Major concerns	No concerns	Major concerns	Very low
TEM vs ZOP	0	Some concerns	Some concerns	No concerns	Major concerns	No concerns	Major concerns	Very low
ZOL vs ZOP	0	Some concerns	Some concerns	No concerns	Major concerns	No concerns	Major concerns	Very low

Sensitivity analysis excluding CPAP titration studies

League table (SMD with 95% confidence interval)

DAR	-0.144 (-0.978, 0.691)	0.663 (-0.402, 1.728)	0.117 (-0.513, 0.746)	0.276 (-0.674, 1.226)	-0.003 (-0.679, 0.674)	-0.392 (-1.312, 0.527)	**-0.663 (-1.233, -0.092)**
	ESZ	0.807 (-0.279, 1.893)	0.260 (-0.404, 0.925)	0.419 (-0.554, 1.393)	0.141 (-0.568, 0.850)	-0.249 (-1.193, 0.695)	-0.519 (-1.128, 0.090)
		FLU	-0.546 (-1.484, 0.391)	-0.388 (-1.565, 0.790)	-0.666 (-1.636, 0.304)	-1.056 (-2.208, 0.097)	**-1.326 (-2.225, -0.426)**
			LEM	0.159 (-0.646, 0.964)	-0.119 (-0.570, 0.331)	-0.509 (-1.278, 0.259)	**-0.779 (-1.045, -0.513)**

				TEM	-0.278 (-1.121, 0.564)	-0.668 (-1.715, 0.379)	**-0.938 (-1.698, -0.178)**
					ZOL	-0.390 (-1.197, 0.418)	**-0.660 (-1.024, -0.296)**
						ZOP	-0.270 (-0.991, 0.451)
							PLA

Global heterogeneity

Between study variance (τ^2): 0.000 (heterogeneity assessment: low)

Random-effects design-by-treatment interaction model

na

Local heterogeneity (I^2) and incoherence (SIDE test)

na

Subgroup analysis including studies involved only non-CPAP users

League table (SMD with 95% confidence interval)

ESZ	0.260 (-0.404, 0.925)	0.141 (-0.568, 0.850)	-0.249 (-1.193, 0.695)	-0.519 (-1.128, 0.090)
	LEM	-0.119 (-0.570, 0.331)	-0.509 (-1.278, 0.259)	**-0.779 (-1.045, -0.513)**
		ZOL	-0.390 (-1.197, 0.418)	**-0.660 (-1.024, -0.296)**
			ZOP	-0.270 (-0.991, 0.451)
				PLA

Global heterogeneity

Between study variance (τ^2): 0.000 (heterogeneity assessment: low)

Random-effects design-by-treatment interaction model

na

Local heterogeneity (I^2) and incoherence (SIDE test)

na

Subgroup analysis including only CPAP titration

	Direct SMD	I^2
ESZ vs PLA	**-0.683 (-0.907, -0.459)**	0.0%

Appendix S5. Sleep efficiency

13 studies, 956 participants

League table (SMD with 95% confidence interval)

DAR	0.060 (-0.553, 0.673)	-0.989 (-2.123, 0.145)	-0.198 (-0.837, 0.441)	0.438 (-0.623, 1.499)	**0.796 (0.051, 1.540)**	0.048 (-0.824, 0.921)	0.315 (-0.480, 1.111)	**0.813 (0.234, 1.391)**	
	ESZ	**-1.049 (-2.045, -0.053)**	-0.258 (-0.597, 0.080)	0.378 (-0.534, 1.291)	**0.736 (0.225, 1.246)**	-0.012 (-0.696, 0.672)	0.255 (-0.327, 0.838)	**0.753 (0.550, 0.956)**	
		FLU	0.790 (-0.222, 1.803)	**1.427 (0.107, 2.747)**	**1.784 (0.703, 2.866)**	1.037 (-0.137, 2.211)	**1.304 (0.186, 2.422)**	**1.801 (0.826, 2.777)**	
			LEM	0.636 (-0.294, 1.566)	**0.994 (0.453, 1.535)**	0.246 (-0.460, 0.953)	0.514 (-0.096, 1.123)	**1.011 (0.740, 1.282)**	
				RAM	0.358 (-0.648, 1.363)	-0.390 (-1.494, 0.714)	-0.123 (-1.167, 0.921)	0.374 (-0.515, 1.264)	
					TEM	-0.748 (-1.551, 0.056)	-0.480 (-1.200, 0.239)	0.017 (-0.452, 0.486)	
						ZOL	0.267 (-0.584, 1.118)	**0.764 (0.112, 1.417)**	
							ZOP	0.497 (-0.049, 1.043)	
								PLA	

SUCRA

DAR	75.5%
ESZ	70.1%
FLU	91.3%
LEM	87.5%
RAM	38.0%
TEM	11.5%
ZOL	72.4%
ZOP	36.0%
PLA	8.5%

Global heterogeneity

Between study variance (τ^2): 0.000 (heterogeneity assessment: low)

Random-effects design-by-treatment interaction model

na

Local heterogeneity (I^2) and incoherence (SIDE test)

	NMA SMD	Direct SMD	I^2	Indirect SMD	P value (SIDE test)
ESZ vs PLA		0.753 (0.550, 0.956)	0.0%		
TEM vs PLA		0.017 (-0.452, 0.486)	0.0%		
ZOP vs PLA		0.497 (-0.049, 1.043)	0.0%		

CINeMA confidence rating

Comparison	Number of studies	Within-study bias	Reporting bias	Indirectness	Imprecision	Heterogeneity	Incoherence	Confidence rating
DAR vs PLA	1	Some concerns	Some concerns	No concerns	No concerns	Major concerns	Major concerns	Very low
ESZ vs PLA	4	No concerns	Some concerns	No concerns	No concerns	No concerns	Major concerns	Low
FLU vs PLA	1	Some concerns	Some concerns	No concerns	No concerns	No concerns	Major concerns	Low
LEM vs PLA	1	Some concerns	Some concerns	No concerns	No concerns	No concerns	Major concerns	Low
RAM vs PLA	1	Some concerns	Some concerns	No concerns	Major concerns	No concerns	Major concerns	Very low
TEM vs PLA	2	No concerns	Some concerns	No concerns	Major concerns	No concerns	Major concerns	Very low
ZOL vs PLA	1	Some concerns	Some concerns	No concerns	No concerns	Major concerns	Major concerns	Very low
ZOP vs PLA	2	Some concerns	Some concerns	No concerns	Major concerns	No concerns	Major concerns	Very low
DAR vs ESZ	0	Some concerns	Some concerns	No concerns	Major concerns	No concerns	Major concerns	Very low
DAR vs FLU	0	Some concerns	Some concerns	No concerns	Major concerns	No concerns	Major concerns	Very low
DAR vs LEM	0	Some concerns	Some concerns	No concerns	Major concerns	No concerns	Major concerns	Very low
DAR vs RAM	0	Some concerns	Some concerns	No concerns	Major concerns	No concerns	Major concerns	Very low
DAR vs TEM	0	Some concerns	Some concerns	No concerns	No concerns	Major concerns	Major concerns	Very low
DAR vs ZOL	0	Some concerns	Some concerns	No concerns	Major concerns	No concerns	Major concerns	Very low
DAR vs ZOP	0	Some concerns	Some concerns	No concerns	Major concerns	No concerns	Major concerns	Very low
ESZ vs FLU	0	Some concerns	Some concerns	No concerns	No concerns	Major concerns	Major concerns	Very low
ESZ vs LEM	0	Some concerns	Some concerns	No concerns	Major concerns	No concerns	Major concerns	Very low
ESZ vs RAM	0	Some concerns	Some concerns	No concerns	Major concerns	No concerns	Major concerns	Very low
ESZ vs TEM	0	No concerns	Some concerns	No concerns	No concerns	No concerns	Major concerns	Very low
ESZ vs ZOL	0	Some concerns	Some concerns	No concerns	Major concerns	No concerns	Major concerns	Very low
ESZ vs ZOP	0	Some concerns	Some concerns	No concerns	Major concerns	No concerns	Major concerns	Very low
FLU vs LEM	0	Some concerns	Some concerns	No concerns	Major concerns	No concerns	Major concerns	Very low
FLU vs RAM	0	Some concerns	Some concerns	No concerns	No concerns	Major concerns	Major concerns	Very low
FLU vs TEM	0	Some concerns	Some concerns	No concerns	No concerns	No concerns	Major concerns	Very low
FLU vs ZOL	0	Some concerns	Some concerns	No concerns	Major concerns	No concerns	Major concerns	Very low
FLU vs ZOP	0	Some concerns	Some concerns	No concerns	No concerns	Major concerns	Major concerns	Very low
LEM vs RAM	0	Some concerns	Some concerns	No concerns	Major concerns	No concerns	Major concerns	Very low
LEM vs TEM	0	Some concerns	Some concerns	No concerns	No concerns	No concerns	Major concerns	Very low
LEM vs ZOL	0	Some concerns	Some concerns	No concerns	Major concerns	No concerns	Major concerns	Very low
LEM vs ZOP	0	Some concerns	Some concerns	No concerns	Major concerns	No concerns	Major concerns	Very low
RAM vs TEM	0	Some concerns	Some concerns	No concerns	Major concerns	No concerns	Major concerns	Very low
RAM vs ZOL	0	Some concerns	Some concerns	No concerns	Major concerns	No concerns	Major concerns	Very low
RAM vs ZOP	0	Some concerns	Some concerns	No concerns	Major concerns	No concerns	Major concerns	Very low

TEM vs ZOL	0	Some concerns	Some concerns	No concerns	Major concerns	No concerns	Major concerns	Very low
TEM vs ZOP	0	Some concerns	Some concerns	No concerns	Major concerns	No concerns	Major concerns	Very low
ZOL vs ZOP	0	Some concerns	Some concerns	No concerns	Major concerns	No concerns	Major concerns	Very low

Sensitivity analysis excluding CPAP titration studies

League table (SMD with 95% confidence interval)

DAR	0.106 (-0.636, 0.847)	-0.989 (-2.123, 0.145)	-0.198 (-0.837, 0.441)	0.438 (-0.623, 1.499)	**0.796 (0.051, 1.540)**	0.048 (-0.824, 0.921)	0.315 (-0.480, 1.111)	**0.813 (0.234, 1.391)**
	ESZ	**-1.094 (-2.174, -0.015)**	-0.304 (-0.841, 0.233)	0.332 (-0.670, 1.335)	**0.690 (0.031, 1.349)**	-0.057 (-0.858, 0.743)	0.210 (-0.506, 0.926)	**0.707 (0.244, 1.170)**
		FLU	0.790 (-0.222, 1.803)	**1.427 (0.107, 2.747)**	**1.784 (0.703, 2.866)**	1.037 (-0.137, 2.211)	**1.304 (0.186, 2.422)**	**1.801 (0.826, 2.777)**
			LEM	0.636 (-0.294, 1.566)	**0.994 (0.453, 1.535)**	0.246 (-0.460, 0.953)	0.514 (-0.096, 1.123)	**1.011 (0.740, 1.282)**
				RAM	0.358 (-0.648, 1.363)	-0.390 (-1.494, 0.714)	-0.123 (-1.167, 0.921)	0.374 (-0.515, 1.264)
					TEM	-0.748 (-1.551, 0.056)	-0.480 (-1.200, 0.239)	0.017 (-0.452, 0.486)
						ZOL	0.267 (-0.584, 1.118)	**0.764 (0.112, 1.417)**
							ZOP	0.497 (-0.049, 1.043)
								PLA

Global heterogeneity

Between study variance (τ^2): 0.000 (heterogeneity assessment: low)

Random-effects design-by-treatment interaction model

na

Local heterogeneity (I^2) and incoherence (SIDE test)

	NMA SMD	Direct SMD	I^2	Indirect SMD	P value (SIDE test)
ESZ vs PLA		0.707 (0.244, 1.170)	0.0%		
TEM vs PLA		0.017 (-0.452, 0.486)	0.0%		
ZOP vs PLA		0.497 (-0.049, 1.043)	0.0%		

Subgroup analysis including studies involved only non-CPAP users

League table (SMD with 95% confidence interval)

ESZ	-0.304 (-0.841, 0.233)	-0.057 (-0.858, 0.743)	0.210 (-0.506, 0.926)	**0.707 (0.244, 1.170)**
	LEM	0.246 (-0.460, 0.953)	0.514 (-0.096, 1.123)	**1.011 (0.740, 1.282)**
		ZOL	0.267 (-0.584, 1.118)	**0.764 (0.112, 1.417)**
			ZOP	0.497 (-0.049, 1.043)
				PLA

Global heterogeneity

Between study variance (τ^2): 0.000 (heterogeneity assessment: low)

Random-effects design-by-treatment interaction model

na

Local heterogeneity (I^2) and incoherence (SIDE test)

	NMA SMD	Direct SMD	I^2	Indirect SMD	P value (SIDE test)
ESZ vs PLA		0.707 (0.244, 1.170)	0.0%		
ZOP vs PLA		0.497 (-0.049, 1.043)	0.0%		

Subgroup analysis including only CPAP titration

	Direct SMD	I^2
ESZ vs PLA	**0.767 (0.542, 0.993)**	0.0%

Appendix S6. Apnea-hypopnea index during total sleep time

20 studies, 1064 participants

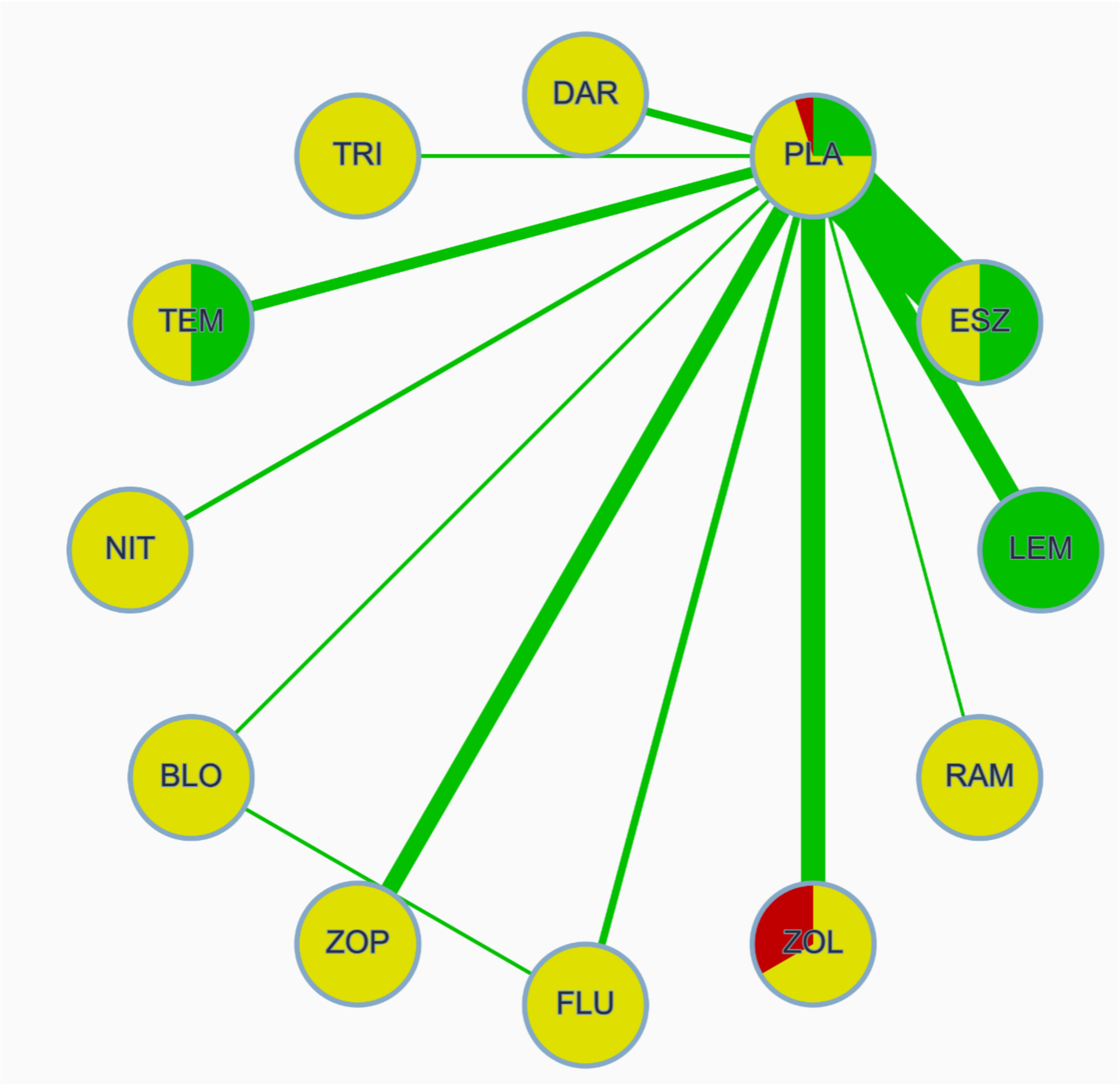

Node size by Equal size

Node color by risk of bias

Green: low overall risk of bias

Yellow: moderate overall risk of bias

Red: high overall risk of bias

Edge width by sample size

Edge color by average indirectness

Green: no indirectness

BLO: brotizolam, DAR: daridorexant, ESZ: eszopiclone, FLU: flurazepam, LEM: lemborexant, NIT: nitrazepam, PLA: placebo, RAM: ramelteon, TEM: temazepam, TRI: triazolam, ZOL: zolpidem, ZOP: zopiclone

League table (SMD with 95% confidence interval)

BLO	-0.221 (-1.154, 0.713)	0.256 (-0.521, 1.032)	-0.291 (-1.043, 0.461)	-0.106 (-0.927, 0.714)	-0.020 (-1.063, 1.024)	-0.315 (-1.474, 0.844)	-0.003 (-0.889, 0.883)	-0.378 (-1.478, 0.723)	0.003 (-0.812, 0.818)	-0.083 (-0.922, 0.757)	-0.114 (-0.865, 0.637)
	DAR	0.476 (-0.112, 1.065)	-0.070 (-0.864, 0.723)	0.114 (-0.532, 0.760)	0.201 (-0.711, 1.113)	-0.094 (-1.138, 0.949)	0.218 (-0.510, 0.945)	-0.157 (-1.134, 0.820)	0.223 (-0.415, 0.862)	0.138 (-0.532, 0.808)	0.107 (-0.448, 0.662)
		ESZ	-0.547 (-1.148, 0.054)	-0.362 (-0.748, 0.024)	-0.276 (-1.026, 0.475)	-0.571 (-1.476, 0.334)	-0.259 (-0.769, 0.252)	-0.633 (-1.462, 0.195)	-0.253 (-0.626, 0.120)	-0.338 (-0.762, 0.086)	**-0.370 (-0.567, -0.172)**
			FLU	0.185 (-0.472, 0.842)	0.271 (-0.649, 1.191)	-0.024 (-1.074, 1.026)	0.288 (-0.449, 1.025)	-0.087 (-1.071, 0.898)	0.294 (-0.356, 0.944)	0.208 (-0.472, 0.889)	0.177 (-0.390, 0.745)
				LEM	0.087 (-0.710, 0.883)	-0.209 (-1.152, 0.735)	0.103 (-0.472, 0.679)	-0.271 (-1.141, 0.599)	0.109 (-0.349, 0.567)	0.024 (-0.477, 0.524)	-0.007 (-0.339, 0.324)
					NIT	-0.295 (-1.438, 0.847)	0.017 (-0.847, 0.880)	-0.358 (-1.440, 0.724)	0.022 (-0.768, 0.813)	-0.063 (-0.878, 0.752)	-0.094 (-0.818, 0.630)
						RAM	0.312 (-0.689, 1.313)	-0.062 (-1.257, 1.132)	0.318 (-0.620, 1.256)	0.232 (-0.727, 1.192)	0.201 (-0.682, 1.085)
							TEM	-0.374 (-1.306, 0.557)	0.006 (-0.561, 0.573)	-0.080 (-0.681, 0.522)	-0.111 (-0.581, 0.360)
								TRI	0.380 (-0.484, 1.244)	0.295 (-0.593, 1.182)	0.264 (-0.540, 1.068)
									ZOL	-0.085 (-0.576, 0.405)	-0.116 (-0.433, 0.200)
										ZOP	-0.031 (-0.406, 0.344)
											PLA

Global heterogeneity

Between study variance (τ^2): 0.000 (heterogeneity assessment: low)

Random-effects design-by-treatment interaction model

p < 0.1 was considered as considerable global inconsistency.

χ^2 statistic: 0.137 (1 degrees of freedom), P value: 0.711

Local heterogeneity (I^2) and incoherence (SIDE test)

	NMA SMD	Direct SMD	I^2	Indirect SMD	P value (SIDE test)
BLO vs FLU	-0.291 (-1.043, 0.461)	-0.237 (-1.041, 0.566)		-0.668 (-2.800, 1.464)	0.711
BLO vs PLA	-0.114 (-0.865, 0.637)	-0.167 (-0.969, 0.635)		0.266 (-1.877, 2.409)	0.711
ESZ vs PLA		-0.370 (-0.567, -0.172)	0.0%		
FLU vs PLA		0.177 (-0.390, 0.745)	0.0%		
LEM vs PLA		-0.007 (-0.339, 0.324)	0.0%		

TEM vs PLA		-0.111 (-0.581, 0.360)	0.0%		
ZOL vs PLA		-0.116 (-0.433, 0.200)	0.0%		
ZOP vs PLA		-0.031 (-0.406, 0.344)	0.0%		

Funnel plot

As none of the comparisons included 10 or more studies, the assessment of publication bias was not performed.

Meta-regression analysis (the placebo was the control)

Potential moderators	Median β (SE)
Proportion of males	0.020 (-0.488, 0.551)
Mean age (years)	-33.387 (-87.143, 58.291)
Mean AHI (events/h)	-0.069 (-0.515, 0.382)
Mean BMI (kg/m^2)	-0.031 (-43.659, 69.872)
CPAP usage	-0.420 (-39.783, 3.840)
Study duration (d)	0.099 (-111.433 - 64.948)
Sponsorship	-45.551 (-92.390, 0.615)
Crossover trial	-21.418 (-76.344, 57.217)
Washout period (d)	4.256 (-79.858, 80.654)
Total number of participants	-26.364 (-62.266, 24.337)
Publication year	0.424 (-7.479, 74.654)
Overall risk of bias	-0.354 (-39.218, 35.751)

CINeMA confidence rating

Comparison	Number of studies	Within-study bias	Reporting bias	Indirectness	Imprecision	Heterogeneity	Incoherence	Confidence rating
BLO vs FLU	1	Some concerns	Some concerns	No concerns	Major concerns	No concerns	No concerns	Low
BLO vs PLA	1	Some concerns	Some concerns	No concerns	Major concerns	No concerns	No concerns	Low
DAR vs PLA	1	Some concerns	Some concerns	No concerns	Major concerns	No concerns	No concerns	Low
ESZ vs PLA	4	No concerns	Some concerns	No concerns	No concerns	No concerns	No concerns	Moderate
FLU vs PLA	2	Some concerns	Some concerns	No concerns	Major concerns	No concerns	No concerns	Low
LEM vs PLA	2	No concerns	Some concerns	No concerns	Major concerns	No concerns	No concerns	Low
NIT vs PLA	1	Some concerns	Some concerns	No concerns	Major concerns	No concerns	No concerns	Low

RAM vs PLA	1	Some concerns	Some concerns	No concerns	Major concerns	No concerns	No concerns	Low
TEM vs PLA	2	No concerns	Some concerns	No concerns	Major concerns	No concerns	No concerns	Low
TRI vs PLA	1	Some concerns	Some concerns	No concerns	Major concerns	No concerns	No concerns	Low
ZOL vs PLA	3	Major concerns	Some concerns	No concerns	Major concerns	No concerns	No concerns	Low
ZOP vs PLA	3	Some concerns	Some concerns	No concerns	Major concerns	No concerns	No concerns	Low
BLO vs DAR	0	Some concerns	Some concerns	No concerns	Major concerns	No concerns	No concerns	Very low
BLO vs ESZ	0	Some concerns	Some concerns	No concerns	Major concerns	No concerns	No concerns	Very low
BLO vs LEM	0	Some concerns	Some concerns	No concerns	Major concerns	No concerns	No concerns	Very low
BLO vs NIT	0	Some concerns	Some concerns	No concerns	Major concerns	No concerns	No concerns	Very low
BLO vs RAM	0	Some concerns	Some concerns	No concerns	Major concerns	No concerns	No concerns	Very low
BLO vs TEM	0	Some concerns	Some concerns	No concerns	Major concerns	No concerns	No concerns	Very low
BLO vs TRI	0	Some concerns	Some concerns	No concerns	Major concerns	No concerns	No concerns	Very low
BLO vs ZOL	0	Some concerns	Some concerns	No concerns	Major concerns	No concerns	No concerns	Very low
BLO vs ZOP	0	Some concerns	Some concerns	No concerns	Major concerns	No concerns	No concerns	Very low
DAR vs ESZ	0	Some concerns	Some concerns	No concerns	Major concerns	No concerns	No concerns	Very low
DAR vs FLU	0	Some concerns	Some concerns	No concerns	Major concerns	No concerns	No concerns	Very low
DAR vs LEM	0	Some concerns	Some concerns	No concerns	Major concerns	No concerns	No concerns	Very low
DAR vs NIT	0	Some concerns	Some concerns	No concerns	Major concerns	No concerns	No concerns	Very low
DAR vs RAM	0	Some concerns	Some concerns	No concerns	Major concerns	No concerns	No concerns	Very low
DAR vs TEM	0	Some concerns	Some concerns	No concerns	Major concerns	No concerns	No concerns	Very low
DAR vs TRI	0	Some concerns	Some concerns	No concerns	Major concerns	No concerns	No concerns	Very low
DAR vs ZOL	0	Some concerns	Some concerns	No concerns	Major concerns	No concerns	No concerns	Very low
DAR vs ZOP	0	Some concerns	Some concerns	No concerns	Major concerns	No concerns	No concerns	Very low
ESZ vs FLU	0	Some concerns	Some concerns	No concerns	Major concerns	No concerns	No concerns	Very low
ESZ vs LEM	0	No concerns	Some concerns	No concerns	Major concerns	No concerns	No concerns	Very low
ESZ vs NIT	0	Some concerns	Some concerns	No concerns	Major concerns	No concerns	No concerns	Very low
ESZ vs RAM	0	Some concerns	Some concerns	No concerns	Major concerns	No concerns	No concerns	Very low
ESZ vs TEM	0	No concerns	Some concerns	No concerns	Major concerns	No concerns	No concerns	Very low
ESZ vs TRI	0	Some concerns	Some concerns	No concerns	Major concerns	No concerns	No concerns	Very low
ESZ vs ZOL	0	Some concerns	Some concerns	No concerns	Major concerns	No concerns	No concerns	Very low
ESZ vs ZOP	0	Some concerns	Some concerns	No concerns	Major concerns	No concerns	No concerns	Very low
FLU vs LEM	0	Some concerns	Some concerns	No concerns	Major concerns	No concerns	No concerns	Very low
FLU vs NIT	0	Some concerns	Some concerns	No concerns	Major concerns	No concerns	No concerns	Very low
FLU vs RAM	0	Some concerns	Some concerns	No concerns	Major concerns	No concerns	No concerns	Very low
FLU vs TEM	0	Some concerns	Some concerns	No concerns	Major concerns	No concerns	No concerns	Very low

FLU vs TRI	0	Some concerns	Some concerns	No concerns	Major concerns	No concerns	No concerns	Very low
FLU vs ZOL	0	Some concerns	Some concerns	No concerns	Major concerns	No concerns	No concerns	Very low
FLU vs ZOP	0	Some concerns	Some concerns	No concerns	Major concerns	No concerns	No concerns	Very low
LEM vs NIT	0	Some concerns	Some concerns	No concerns	Major concerns	No concerns	No concerns	Very low
LEM vs RAM	0	Some concerns	Some concerns	No concerns	Major concerns	No concerns	No concerns	Very low
LEM vs TEM	0	No concerns	Some concerns	No concerns	Major concerns	No concerns	No concerns	Very low
LEM vs TRI	0	Some concerns	Some concerns	No concerns	Major concerns	No concerns	No concerns	Very low
LEM vs ZOL	0	Some concerns	Some concerns	No concerns	Major concerns	No concerns	No concerns	Very low
LEM vs ZOP	0	Some concerns	Some concerns	No concerns	Major concerns	No concerns	No concerns	Very low
NIT vs RAM	0	Some concerns	Some concerns	No concerns	Major concerns	No concerns	No concerns	Very low
NIT vs TEM	0	Some concerns	Some concerns	No concerns	Major concerns	No concerns	No concerns	Very low
NIT vs TRI	0	Some concerns	Some concerns	No concerns	Major concerns	No concerns	No concerns	Very low
NIT vs ZOL	0	Some concerns	Some concerns	No concerns	Major concerns	No concerns	No concerns	Very low
NIT vs ZOP	0	Some concerns	Some concerns	No concerns	Major concerns	No concerns	No concerns	Very low
RAM vs TEM	0	Some concerns	Some concerns	No concerns	Major concerns	No concerns	No concerns	Very low
RAM vs TRI	0	Some concerns	Some concerns	No concerns	Major concerns	No concerns	No concerns	Very low
RAM vs ZOL	0	Some concerns	Some concerns	No concerns	Major concerns	No concerns	No concerns	Very low
RAM vs ZOP	0	Some concerns	Some concerns	No concerns	Major concerns	No concerns	No concerns	Very low
TEM vs TRI	0	Some concerns	Some concerns	No concerns	Major concerns	No concerns	No concerns	Very low
TEM vs ZOL	0	Some concerns	Some concerns	No concerns	Major concerns	No concerns	No concerns	Very low
TEM vs ZOP	0	Some concerns	Some concerns	No concerns	Major concerns	No concerns	No concerns	Very low
TRI vs ZOL	0	Some concerns	Some concerns	No concerns	Major concerns	No concerns	No concerns	Very low
TRI vs ZOP	0	Some concerns	Some concerns	No concerns	Major concerns	No concerns	No concerns	Very low
ZOL vs ZOP	0	Some concerns	Some concerns	No concerns	Major concerns	No concerns	No concerns	Very low

Sensitivity analysis excluding CPAP titration studies

League table (SMD with 95% confidence interval)

BLO	-0.221 (-1.154, 0.713)	0.034 (-0.841, 0.908)	-0.291 (-1.043, 0.461)	-0.106 (-0.927, 0.714)	-0.020 (-1.063, 1.024)	-0.315 (-1.474, 0.844)	-0.003 (-0.889, 0.883)	-0.378 (-1.478, 0.723)	0.003 (-0.812, 0.818)	-0.083 (-0.922, 0.757)	-0.114 (-0.865, 0.637)
	DAR	0.254 (-0.459, 0.968)	-0.070 (-0.864, 0.723)	0.114 (-0.532, 0.760)	0.201 (-0.711, 1.113)	-0.094 (-1.138, 0.949)	0.218 (-0.510, 0.945)	-0.157 (-1.134, 0.820)	0.223 (-0.415, 0.862)	0.138 (-0.532, 0.808)	0.107 (-0.448, 0.662)
		ESZ	-0.325 (-1.048, 0.399)	-0.140 (-0.698, 0.418)	-0.053 (-0.905, 0.799)	-0.349 (-1.339, 0.642)	-0.037 (-0.687, 0.614)	-0.411 (-1.332, 0.510)	-0.031 (-0.580, 0.518)	-0.116 (-0.701, 0.468)	-0.147 (-0.596, 0.301)
			FLU	0.185 (-0.472, 0.842)	0.271 (-0.649, 1.191)	-0.024 (-1.074, 1.026)	0.288 (-0.449, 1.025)	-0.087 (-1.071, 0.898)	0.294 (-0.356, 0.944)	0.208 (-0.472, 0.889)	0.177 (-0.390, 0.745)
				LEM	0.087 (-0.710, 0.883)	-0.209 (-1.152, 0.735)	0.103 (-0.472, 0.679)	-0.271 (-1.141, 0.599)	0.109 (-0.349, 0.567)	0.024 (-0.477, 0.524)	-0.007 (-0.339, 0.324)
					NIT	-0.295 (-1.438, 0.847)	0.017 (-0.847, 0.880)	-0.358 (-1.440, 0.724)	0.022 (-0.768, 0.813)	-0.063 (-0.878, 0.752)	-0.094 (-0.818, 0.630)

						RAM	0.312 (-0.689, 1.313)	-0.062 (-1.257, 1.132)	0.318 (-0.620, 1.256)	0.232 (-0.727, 1.192)	0.201 (-0.682, 1.085)
							TEM	-0.374 (-1.306, 0.557)	0.006 (-0.561, 0.573)	-0.080 (-0.681, 0.522)	-0.111 (-0.581, 0.360)
								TRI	0.380 (-0.484, 1.244)	0.295 (-0.593, 1.182)	0.264 (-0.540, 1.068)
									ZOL	-0.085 (-0.576, 0.405)	-0.116 (-0.433, 0.200)
										ZOP	-0.031 (-0.406, 0.344)
											PLA

Global heterogeneity

Between study variance (τ^2): 0.000 (heterogeneity assessment: low)

Random-effects design-by-treatment interaction model

χ^2 statistic: 0.137 (1 degrees of freedom), P value: 0.711

Local heterogeneity (I^2) and incoherence (SIDE test)

	NMA SMD	Direct SMD	I^2	Indirect SMD	P value (SIDE test)
BLO vs FLU	-0.291 (-1.043, 0.461)	-0.237 (-1.041, 0.566)		-0.668 (-2.800, 1.464)	0.711
BLO vs PLA	-0.114 (-0.865, 0.637)	-0.167 (-0.969, 0.635)		0.266 (-1.877, 2.409)	0.711
ESZ vs PLA		-0.147 (-0.596, 0.301)	0.0%		
FLU vs PLA		0.177 (-0.390, 0.745)	0.0%		
LEM vs PLA		-0.007 (-0.339, 0.324)	0.0%		
TEM vs PLA		-0.111 (-0.581, 0.360)	0.0%		
ZOL vs PLA		-0.116 (-0.433, 0.200)	0.0%		
ZOP vs PLA		-0.031 (-0.406, 0.344)	0.0%d		

Subgroup analysis including studies involved only non-CPAP users

League table (SMD with 95% confidence interval)

ESZ	-0.140 (-0.698, 0.418)	-0.125 (-0.696, 0.446)	-0.116 (-0.701, 0.468)	-0.147 (-0.596, 0.301)
	LEM	0.015 (-0.470, 0.500)	0.024 (-0.477, 0.524)	-0.007 (-0.339, 0.324)

		ZOL	0.009 (-0.507, 0.524)	-0.022 (-0.376, 0.331)
			ZOP	-0.031 (-0.406, 0.344)
				PLA

Global heterogeneity

Between study variance (τ^2): 0.000 (heterogeneity assessment: low)

Random-effects design-by-treatment interaction model

na

Local heterogeneity (I^2) and incoherence (SIDE test)

	NMA SMD	Direct SMD	I^2	Indirect SMD	P value (SIDE test)
ESZ vs PLA		-0.147 (-0.596, 0.301)	0.0%		
LEM vs PLA		-0.007 (-0.339, 0.324)	0.0%		
ZOL vs PLA		-0.022 (-0.376, 0.331)	0.0%		
ZOP vs PLA		-0.031 (-0.406, 0.344)	0.0%		

Subgroup analysis including only CPAP titration

	Direct SMD	I^2
ESZ vs PLA	**-0.425 (-0.646, -0.205)**	0.0%

Network meta-analysis by classifying medications into their drug classes

League table (SMD with 95% confidence interval)

BENZs	-0.017 (-0.426, 0.392)	-0.196 (-1.126, 0.735)	0.102 (-0.261, 0.464)	0.006 (-0.288, 0.300)
	DORAs	-0.179 (-1.107, 0.749)	0.119 (-0.237, 0.474)	0.023 (-0.262, 0.307)
		RAM	0.297 (-0.611, 1.206)	0.201 (-0.682, 1.085)
			Z drugs	-0.096 (-0.309, 0.117)
				PLA

Global heterogeneity

Between study variance (τ^2): 0.000 (heterogeneity assessment: low)

Random-effects design-by-treatment interaction model

na

Local heterogeneity (I^2) and incoherence (SIDE test)

	NMA SMD	Direct SMD	I^2	Indirect SMD	P value (SIDE test)
BENZs vs PLA			0.0%		
DORAs vs PLA			0.0%		
Z drugs vs PLA			0.0%		

Appendix S7. Apnea-hypopnea index during non-rapid eye movement sleep

7 studies, 336 participants

League table (SMD with 95% confidence interval)

DAR	0.406 (-0.470, 1.281)	0.090 (-0.556, 0.736)	0.175 (-0.713, 1.063)	0.086 (-0.621, 0.793)	0.074 (-0.480, 0.629)	
	ESZ	-0.316 (-1.070, 0.438)	-0.231 (-1.200, 0.739)	-0.320 (-1.127, 0.487)	-0.332 (-1.009, 0.346)	
		LEM	0.086 (-0.683, 0.854)	-0.004 (-0.553, 0.546)	-0.015 (-0.347, 0.316)	
			ZOL	-0.089 (-0.910, 0.731)	-0.101 (-0.794, 0.593)	
				ZOP	-0.011 (-0.450, 0.427)	
					PLA	

SUCRA

DAR	36.9%
ESZ	75.7%
LEM	55.9%
ZOL	63.6%
ZOP	53.0%
PLA	50.8%

Global heterogeneity

Between study variance (τ^2): 0.000 (heterogeneity assessment: low)

Random-effects design-by-treatment interaction model

na

Local heterogeneity (I^2) and incoherence (SIDE test)

	NMA SMD	Direct SMD	I^2	Indirect SMD	P value (SIDE test)
LEM vs PLA		-0.015 (-0.347, 0.316)	0.0%		
ZOP vs PLA		-0.011 (-0.450, 0.427)	0.0%		

CINeMA confidence rating

Comparison	Number of studies	Within-study bias	Reporting bias	Indirectness	Imprecision	Heterogeneity	Incoherence	Confidence rating
DAR vs PLA	1	Some concerns	Some concerns	No concerns	Major concerns	No concerns	Major concerns	Very low
ESZ vs PLA	1	Some concerns	Some concerns	No concerns	Major concerns	No concerns	Major concerns	Very low
LEM vs PLA	2	No concerns	Some concerns	No concerns	Major concerns	No concerns	Major concerns	Very low
ZOL vs PLA	1	Some concerns	Some concerns	No concerns	Major concerns	No concerns	Major concerns	Very low
ZOP vs PLA	2	Some concerns	Some concerns	No concerns	Major concerns	No concerns	Major concerns	Very low

DAR vs ESZ	0	Some concerns	Some concerns	No concerns	Major concerns	No concerns	Major concerns	Very low
DAR vs LEM	0	Some concerns	Some concerns	No concerns	Major concerns	No concerns	Major concerns	Very low
DAR vs ZOL	0	Some concerns	Some concerns	No concerns	Major concerns	No concerns	Major concerns	Very low
DAR vs ZOP	0	Some concerns	Some concerns	No concerns	Major concerns	No concerns	Major concerns	Very low
ESZ vs LEM	0	Some concerns	Some concerns	No concerns	Major concerns	No concerns	Major concerns	Very low
ESZ vs ZOL	0	Some concerns	Some concerns	No concerns	Major concerns	No concerns	Major concerns	Very low
ESZ vs ZOP	0	Some concerns	Some concerns	No concerns	Major concerns	No concerns	Major concerns	Very low
LEM vs ZOL	0	Some concerns	Some concerns	No concerns	Major concerns	No concerns	Major concerns	Very low
LEM vs ZOP	0	Some concerns	Some concerns	No concerns	Major concerns	No concerns	Major concerns	Very low
ZOL vs ZOP	0	Some concerns	Some concerns	No concerns	Major concerns	No concerns	Major concerns	Very low

Sensitivity analysis excluding CPAP titration studies

League table (SMD with 95% confidence interval)

DAR	0.406 (-0.470, 1.281)	0.090 (-0.556, 0.736)	0.175 (-0.713, 1.063)	0.086 (-0.621, 0.793)	0.074 (-0.480, 0.629)
	ESZ	-0.316 (-1.070, 0.438)	-0.231 (-1.200, 0.739)	-0.320 (-1.127, 0.487)	-0.332 (-1.009, 0.346)
		LEM	0.086 (-0.683, 0.854)	-0.004 (-0.553, 0.546)	-0.015 (-0.347, 0.316)
			ZOL	-0.089 (-0.910, 0.731)	-0.101 (-0.794, 0.593)
				ZOP	-0.011 (-0.450, 0.427)
					PLA

Global heterogeneity

Between study variance (τ^2): 0.000 (heterogeneity assessment: low)

Random-effects design-by-treatment interaction model

na

Local heterogeneity (I^2) and incoherence (SIDE test)

	NMA SMD	Direct SMD	I^2	Indirect SMD	P value (SIDE test)
LEM vs PLA		-0.015 (-0.347, 0.316)	0.0%		
ZOP vs PLA		-0.011 (-0.450, 0.427)	0.0%		

Subgroup analysis including studies involved only non-CPAP users

League table (SMD with 95% confidence interval)

ESZ	-0.316 (-1.070, 0.438)	-0.320 (-1.127, 0.487)	-0.332 (-1.009, 0.346)
	LEM	-0.004 (-0.553, 0.546)	-0.015 (-0.347, 0.316)
		ZOP	-0.011 (-0.450, 0.427)
			PLA

Global heterogeneity

Between study variance (τ^2): 0.000 (heterogeneity assessment: low)

Random-effects design-by-treatment interaction model

na

Local heterogeneity (I^2) and incoherence (SIDE test)

	NMA SMD	Direct SMD	I^2	Indirect SMD	P value (SIDE test)
LEM vs PLA		-0.015 (-0.347, 0.316)	0.0%		
ZOP vs PLA		-0.011 (-0.450, 0.427)	0.0%		

Network meta-analysis by classifying medications into their drug classes

League table (SMD with 95% confidence interval)

DORAs	0.113 (-0.319, 0.545)	0.008 (-0.276, 0.293)
	Z drugs	-0.105 (-0.430, 0.220)
		PLA

Global heterogeneity

Between study variance (τ^2): 0.000 (heterogeneity assessment: low)

Random-effects design-by-treatment interaction model

na

Local heterogeneity (I^2) and incoherence (SIDE test)

	NMA SMD	Direct SMD	I^2	Indirect SMD	P value (SIDE test)
DORAs vs PLA		0.008 (-0.276, 0.293)	0.0%		
Z drugs vs PLA		-0.105 (-0.430, 0.220)	0.0%		

Appendix S8. Apnea-hypopnea index during rapid eye movement sleep

8 studies, 420 participants

League table (SMD with 95% confidence interval)

DAR	0.272 (-0.601, 1.144)	0.214 (-0.432, 0.861)	0.183 (-0.480, 0.847)	0.323 (-0.385, 1.031)	0.100 (-0.455, 0.655)	
	ESZ	-0.057 (-0.808, 0.694)	-0.088 (-0.854, 0.678)	0.051 (-0.753, 0.856)	-0.172 (-0.845, 0.502)	
		LEM	-0.031 (-0.524, 0.462)	0.108 (-0.442, 0.659)	-0.114 (-0.446, 0.217)	
			ZOL	0.139 (-0.432, 0.710)	-0.083 (-0.448, 0.281)	
				ZOP	-0.223 (-0.662, 0.217)	
					PLA	

SUCRA

DAR	36.7%
ESZ	68.7%
LEM	59.5%
ZOL	54.5%
ZOP	70.6%
PLA	44.7%

Global heterogeneity

Between study variance (τ^2): 0.000 (heterogeneity assessment: low)

Random-effects design-by-treatment interaction model

na

Local heterogeneity (I^2) and incoherence (SIDE test)

	NMA SMD	Direct SMD	I^2	Indirect SMD	P value (SIDE test)
LEM vs PLA		-0.114 (-0.446, 0.217)	0.0%		
ZOL vs PLA		-0.083 (-0.448, 0.281)	0.0%		
ZOP vs PLA		-0.223 (-0.662, 0.217)	0.0%		

CINeMA confidence rating

Comparison	Number of studies	Within-study bias	Reporting bias	Indirectness	Imprecision	Heterogeneity	Incoherence	Confidence rating
DAR vs PLA	1	Some concerns	Some concerns	No concerns	Major concerns	No concerns	Major concerns	Very low
ESZ vs PLA	1	Some concerns	Some concerns	No concerns	Major concerns	No concerns	Major concerns	Very low
LEM vs PLA	2	No concerns	Some concerns	No concerns	Major concerns	No concerns	Major concerns	Very low
ZOL vs PLA	2	Major concerns	Some concerns	No concerns	Major concerns	No concerns	Major concerns	Very low

ZOP vs PLA	2	Some concerns	Some concerns	No concerns	Major concerns	No concerns	Major concerns	Very low
DAR vs ESZ	0	Some concerns	Some concerns	No concerns	Major concerns	No concerns	Major concerns	Very low
DAR vs LEM	0	Some concerns	Some concerns	No concerns	Major concerns	No concerns	Major concerns	Very low
DAR vs ZOL	0	Some concerns	Some concerns	No concerns	Major concerns	No concerns	Major concerns	Very low
DAR vs ZOP	0	Some concerns	Some concerns	No concerns	Major concerns	No concerns	Major concerns	Very low
ESZ vs LEM	0	Some concerns	Some concerns	No concerns	Major concerns	No concerns	Major concerns	Very low
ESZ vs ZOL	0	Some concerns	Some concerns	No concerns	Major concerns	No concerns	Major concerns	Very low
ESZ vs ZOP	0	Some concerns	Some concerns	No concerns	Major concerns	No concerns	Major concerns	Very low
LEM vs ZOL	0	Some concerns	Some concerns	No concerns	Major concerns	No concerns	Major concerns	Very low
LEM vs ZOP	0	Some concerns	Some concerns	No concerns	Major concerns	No concerns	Major concerns	Very low
ZOL vs ZOP	0	Some concerns	Some concerns	No concerns	Major concerns	No concerns	Major concerns	Very low

Sensitivity analysis excluding CPAP titration studies

League table (SMD with 95% confidence interval)

DAR	0.272 (-0.601, 1.144)	0.214 (-0.432, 0.861)	0.183 (-0.480, 0.847)	0.323 (-0.385, 1.031)	0.100 (-0.455, 0.655)
	ESZ	-0.057 (-0.808, 0.694)	-0.088 (-0.854, 0.678)	0.051 (-0.753, 0.856)	-0.172 (-0.845, 0.502)
		LEM	-0.031 (-0.524, 0.462)	0.108 (-0.442, 0.659)	-0.114 (-0.446, 0.217)
			ZOL	0.139 (-0.432, 0.710)	-0.083 (-0.448, 0.281)
				ZOP	-0.223 (-0.662, 0.217)
					PLA

Global heterogeneity

Between study variance (τ^2): 0.000 (heterogeneity assessment: low)

Random-effects design-by-treatment interaction model

na

Local heterogeneity (I^2) and incoherence (SIDE test)

	NMA SMD	Direct SMD	I^2	Indirect SMD	P value (SIDE test)
LEM vs PLA		-0.114 (-0.446, 0.217)	0.0%		

ZOL vs PLA		-0.083 (-0.448, 0.281)	0.0%		
ZOP vs PLA		-0.223 (-0.662, 0.217)	0.0%		

Subgroup analysis including studies involved only non-CPAP users

League table (SMD with 95% confidence interval)

ESZ	-0.057 (-0.808, 0.694)	-0.071 (-0.869, 0.727)	0.051 (-0.753, 0.856)	-0.172 (-0.845, 0.502)
	LEM	-0.014 (-0.555, 0.528)	0.108 (-0.442, 0.659)	-0.114 (-0.446, 0.217)
		ZOL	0.122 (-0.492, 0.736)	-0.101 (-0.529, 0.327)
			ZOP	-0.223 (-0.662, 0.217)
				PLA

Global heterogeneity

Between study variance (τ^2): 0.000 (heterogeneity assessment: low)

Random-effects design-by-treatment interaction model

na

Local heterogeneity (I^2) and incoherence (SIDE test)

	NMA SMD	Direct SMD	I^2	Indirect SMD	P value (SIDE test)
LEM vs PLA		-0.114 (-0.446, 0.217)	0.0%		
ZOP vs PLA		-0.223 (-0.662, 0.217)	0.0%		

Network meta-analysis by classifying medications into their drug classes

League table (SMD with 95% confidence interval)

DORAs	0.087 (-0.298, 0.472)	-0.058 (-0.342, 0.227)
	Z drugs	-0.145 (-0.404, 0.114)
		PLA

Global heterogeneity

Between study variance (τ^2): 0.000 (heterogeneity assessment: low)

Random-effects design-by-treatment interaction model

na

Local heterogeneity (I^2) and incoherence (SIDE test)

	NMA SMD	Direct SMD	I^2	Indirect SMD	P value (SIDE test)
DORAs vs PLA		-0.058 (-0.342, 0.227)	0.0%		
Z drugs vs PLA		-0.145 (-0.404, 0.114)	0.0%		

Appendix S9. Mean SpO2 during total sleep time

12 studies, 555 participants

League table (SMD with 95% confidence interval) **SCURA**

BLO	-0.270 (-1.197, 0.657)	-0.553 (-1.561, 0.454)	0.215 (-0.527, 0.957)	-0.113 (-0.926, 0.699)	0.688 (-0.364, 1.740)	0.030 (-0.792, 0.852)	0.137 (-0.695, 0.969)	-0.089 (-0.830, 0.653)		BLO	46.1%
	DAR	-0.283 (-1.163, 0.597)	0.485 (-0.282, 1.252)	0.157 (-0.490, 0.804)	**0.958 (0.028, 1.888)**	0.300 (-0.358, 0.959)	0.407 (-0.265, 1.079)	0.181 (-0.374, 0.737)		DAR	60.4%
		ESZ	0.768 (-0.095, 1.631)	0.440 (-0.319, 1.199)	**1.241 (0.230, 2.252)**	0.584 (-0.185, 1.352)	0.690 (-0.090, 1.470)	0.464 (-0.218, 1.147)		ESZ	78.2%
			FLU	-0.328 (-0.952, 0.296)	0.473 (-0.441, 1.387)	-0.185 (-0.820, 0.451)	-0.078 (-0.727, 0.571)	-0.304 (-0.832, 0.224)		FLU	29.9%
				LEM	0.801 (-0.016, 1.618)	0.144 (-0.342, 0.629)	0.250 (-0.252, 0.753)	0.024 (-0.307, 0.356)		LEM	54.2%
					TEM	-0.658 (-1.483, 0.168)	-0.551 (-1.387, 0.285)	**-0.777 (-1.523, -0.031)**		TEM	10.2%
						ZOL	0.107 (-0.410, 0.624)	-0.119 (-0.473, 0.235)		ZOL	38.3%
							ZOP	-0.226 (-0.603, 0.151)		ZOP	29.6%
								PLA		PLA	53.2%

Global heterogeneity

Between study variance (τ^2): 0.000 (heterogeneity assessment: low)

Random-effects design-by-treatment interaction model

χ^2 statistic: 0.336 (1 degrees of freedom), P value: 0.562

Local heterogeneity (I^2) and incoherence (SIDE test)

	NMA SMD	Direct SMD	I^2	Indirect SMD	P value (SIDE test)
BLO vs FLU	0.215(-0.527,0.957)	0.126(-0.675,0.927)		0.755(-1.215,2.724)	0.562
BLO vs PLA	-0.089(-0.831,0.653)	0.000(-0.800,0.800)		-0.630(-2.606,1.345)	0.562
FLU vs PLA		-0.304 (-0.832, 0.224)	0.0%		
LEM vs PLA		0.024 (-0.307, 0.356)	0.0%		
ZOL vs PLA		-0.119 (-0.473, 0.235)	0.0%		

ZOP vs PLA		-0.226 (-0.603, 0.151)	0.0%		

CINeMA confidence rating

Comparison	Number of studies	Within-study bias	Reporting bias	Indirectness	Imprecision	Heterogeneity	Incoherence	Confidence rating
BLO vs FLU	1	Some concerns	Some concerns	No concerns	Major concerns	No concerns	No concerns	Low
BLO vs PLA	1	Some concerns	Some concerns	No concerns	Major concerns	No concerns	No concerns	Low
DAR vs PLA	1	Some concerns	Some concerns	No concerns	Major concerns	No concerns	No concerns	Low
ESZ vs PLA	1	Some concerns	Some concerns	No concerns	Major concerns	No concerns	No concerns	Low
FLU vs PLA	2	Some concerns	Some concerns	No concerns	Major concerns	No concerns	No concerns	Low
LEM vs PLA	2	No concerns	Some concerns	No concerns	Major concerns	No concerns	No concerns	Low
TEM vs PLA	1	Some concerns	Some concerns	No concerns	No concerns	Major concerns	No concerns	Low
ZOL vs PLA	2	Major concerns	Some concerns	No concerns	Major concerns	No concerns	No concerns	Very low
ZOP vs PLA	3	Some concerns	Some concerns	No concerns	Major concerns	No concerns	No concerns	Low
BLO vs DAR	0	Some concerns	Some concerns	No concerns	Major concerns	No concerns	No concerns	Very low
BLO vs ESZ	0	Some concerns	Some concerns	No concerns	Major concerns	No concerns	No concerns	Very low
BLO vs LEM	0	Some concerns	Some concerns	No concerns	Major concerns	No concerns	No concerns	Very low
BLO vs TEM	0	Some concerns	Some concerns	No concerns	Major concerns	No concerns	No concerns	Very low
BLO vs ZOL	0	Some concerns	Some concerns	No concerns	Major concerns	No concerns	No concerns	Very low
BLO vs ZOP	0	Some concerns	Some concerns	No concerns	Major concerns	No concerns	No concerns	Very low
DAR vs ESZ	0	Some concerns	Some concerns	No concerns	Major concerns	No concerns	No concerns	Very low
DAR vs FLU	0	Some concerns	Some concerns	No concerns	Major concerns	No concerns	No concerns	Very low
DAR vs LEM	0	Some concerns	Some concerns	No concerns	Major concerns	No concerns	No concerns	Very low
DAR vs TEM	0	Some concerns	Some concerns	No concerns	No concerns	Major concerns	No concerns	Very low
DAR vs ZOL	0	Some concerns	Some concerns	No concerns	Major concerns	No concerns	No concerns	Very low
DAR vs ZOP	0	Some concerns	Some concerns	No concerns	Major concerns	No concerns	No concerns	Very low
ESZ vs FLU	0	Some concerns	Some concerns	No concerns	Major concerns	No concerns	No concerns	Very low
ESZ vs LEM	0	Some concerns	Some concerns	No concerns	Major concerns	No concerns	No concerns	Very low
ESZ vs TEM	0	Some concerns	Some concerns	No concerns	No concerns	Major concerns	No concerns	Very low
ESZ vs ZOL	0	Some concerns	Some concerns	No concerns	Major concerns	No concerns	No concerns	Very low
ESZ vs ZOP	0	Some concerns	Some concerns	No concerns	Major concerns	No concerns	No concerns	Very low
FLU vs LEM	0	Some concerns	Some concerns	No concerns	Major concerns	No concerns	No concerns	Very low
FLU vs TEM	0	Some concerns	Some concerns	No concerns	Major concerns	No concerns	No concerns	Very low
FLU vs ZOL	0	Some concerns	Some concerns	No concerns	Major concerns	No concerns	No concerns	Very low
FLU vs ZOP	0	Some concerns	Some concerns	No concerns	Major concerns	No concerns	No concerns	Very low
LEM vs TEM	0	Some concerns	Some concerns	No concerns	Major concerns	No concerns	No concerns	Very low

LEM vs ZOL	0	Some concerns	Some concerns	No concerns	Major concerns	No concerns	No concerns	Very low
LEM vs ZOP	0	Some concerns	Some concerns	No concerns	Major concerns	No concerns	No concerns	Very low
TEM vs ZOL	0	Some concerns	Some concerns	No concerns	Major concerns	No concerns	No concerns	Very low
TEM vs ZOP	0	Some concerns	Some concerns	No concerns	Major concerns	No concerns	No concerns	Very low
ZOL vs ZOP	0	Some concerns	Some concerns	No concerns	Major concerns	No concerns	No concerns	Very low

Sensitivity analysis excluding CPAP titration studies

League table (SMD with 95% confidence interval)

BLO	-0.270 (-1.197, 0.657)	-0.553 (-1.561, 0.454)	0.215 (-0.527, 0.957)	-0.113 (-0.926, 0.699)	0.688 (-0.364, 1.740)	0.030 (-0.792, 0.852)	0.137 (-0.695, 0.969)	-0.089 (-0.830, 0.653)
	DAR	-0.283 (-1.163, 0.597)	0.485 (-0.282, 1.252)	0.157 (-0.490, 0.804)	**0.958 (0.028, 1.888)**	0.300 (-0.358, 0.959)	0.407 (-0.265, 1.079)	0.181 (-0.374, 0.737)
		ESZ	0.768 (-0.095, 1.631)	0.440 (-0.319, 1.199)	**1.241 (0.230, 2.252)**	0.584 (-0.185, 1.352)	0.690 (-0.090, 1.470)	0.464 (-0.218, 1.147)
			FLU	-0.328 (-0.952, 0.296)	0.473 (-0.441, 1.387)	-0.185 (-0.820, 0.451)	-0.078 (-0.727, 0.571)	-0.304 (-0.832, 0.224)
				LEM	0.801 (-0.016, 1.618)	0.144 (-0.342, 0.629)	0.250 (-0.252, 0.753)	0.024 (-0.307, 0.356)
					TEM	-0.658 (-1.483, 0.168)	-0.551 (-1.387, 0.285)	**-0.777 (-1.523, -0.031)**
						ZOL	0.107 (-0.410, 0.624)	-0.119 (-0.473, 0.235)
							ZOP	-0.226 (-0.603, 0.151)
								PLA

Global heterogeneity

Between study variance (τ^2): 0.000 (heterogeneity assessment: low)

Random-effects design-by-treatment interaction model

na

Local heterogeneity (I^2) and incoherence (SIDE test)

	NMA SMD	Direct SMD	I^2	Indirect SMD	P value (SIDE test)
BLO vs FLU	0.215(-0.527,0.957)	0.126(-0.675,0.927)		0.755(-1.215,2.724)	0.562
BLO vs PLA	-0.089(-0.831,0.653)	0.000(-0.800,0.800)		-0.630(-2.606,1.345)	0.562

FLU vs PLA		-0.304 (-0.832, 0.224)	0.0%		
LEM vs PLA		0.024 (-0.307, 0.356)	0.0%		
ZOL vs PLA		-0.119 (-0.473, 0.235)	0.0%		
ZOP vs PLA		-0.226 (-0.603, 0.151)	0.0%		

Subgroup analysis including studies involved only non-CPAP users

League table (SMD with 95% confidence interval)

ESZ	0.440 (-0.319, 1.199)	0.584 (-0.185, 1.352)	0.690 (-0.090, 1.470)	0.464 (-0.218, 1.147)
	LEM	0.144 (-0.342, 0.629)	0.250 (-0.252, 0.753)	0.024 (-0.307, 0.356)
		ZOL	0.107 (-0.410, 0.624)	-0.119 (-0.473, 0.235)
			ZOP	-0.226 (-0.603, 0.151)
				PLA

Global heterogeneity

Between study variance (τ^2): 0.000 (heterogeneity assessment: low)

Random-effects design-by-treatment interaction model

na

Local heterogeneity (I^2) and incoherence (SIDE test)

	NMA SMD	Direct SMD	I^2	Indirect SMD	P value (SIDE test)
LEM vs PLA		0.024 (-0.307, 0.356)	0.0%		
ZOL vs PLA		-0.119 (-0.473, 0.235)	0.0%		
ZOP vs PLA		-0.226 (-0.603, 0.151)	0.0%		

Network meta-analysis by classifying medications into their drug classes

League table (SMD with 95% confidence interval)

BENZs	-0.476 (-0.977, 0.024)	-0.320 (-0.798, 0.157)	**-0.410 (-0.822, -0.001)**

	DORAs	0.156 (-0.218, 0.529)	0.066 (-0.219, 0.351)
		Z drugs	-0.090 (-0.331, 0.152)
			PLA

Global heterogeneity

Between study variance (τ^2): 0.000 (heterogeneity assessment: low)

Random-effects design-by-treatment interaction model

na

Local heterogeneity (I^2) and incoherence (SIDE test)

	NMA SMD	Direct SMD	I^2	Indirect SMD	P value (SIDE test)
BENZs vs PLA		-0.410 (-0.822, -0.001)	0.0%		
DORAs vs PLA		0.066 (-0.219, 0.351)	0.0%		
Z drugs vs PLA		-0.090 (-0.331, 0.152)	0.0%		

Appendix S10. Mean SpO2 nadir during total sleep time

8 studies, 333 participants

League table (SMD with 95% confidence interval)

BLO	0.072 (-0.751, 0.895)	0.065 (-0.683, 0.814)	-0.255 (-1.228, 0.718)	-0.006 (-0.887, 0.876)	0.078 (-0.959, 1.116)	-0.065 (-0.814, 0.683)	
	ESZ	-0.006 (-0.669, 0.656)	-0.327 (-1.036, 0.382)	-0.077 (-0.655, 0.500)	0.007 (-0.789, 0.802)	-0.137 (-0.479, 0.205)	
		FLU	-0.320 (-1.162, 0.521)	-0.071 (-0.805, 0.663)	0.013 (-0.902, 0.928)	-0.131 (-0.698, 0.437)	
			TEM	0.249 (-0.527, 1.026)	0.333 (-0.616, 1.283)	0.190 (-0.432, 0.811)	
				ZOL	0.084 (-0.772, 0.940)	-0.060 (-0.525, 0.406)	
					ZOP	-0.144 (-0.862, 0.575)	
						PLA	

SUCRA

BLO	43.3%
ESZ	33.8%
FLU	39.2%
TEM	62.1%
ZOL	40.6%
ZOP	32.7%
PLA	48.8%

Global heterogeneity

Between study variance (τ^2): 0.000 (heterogeneity assessment: low)

Random-effects design-by-treatment interaction model

χ^2 statistic: 0.206 (1 degrees of freedom), P value: 0.650

Local heterogeneity (I^2) and incoherence (SIDE test)

	NMA SMD	Direct SMD	I^2	Indirect SMD	P value (SIDE test)
BLO vs FLU	0.065(-0.683,0.814)	0.000(-0.800,0.800)		0.525(-1.598,2.648)	0.650
BLO vs PLA	-0.065(-0.814,0.683)	0.000(-0.800,0.800)		-0.525(-2.648,1.598)	0.650
ESZ vs PLA		-0.137 (-0.479, 0.205)	0.0%		
FLU vs PLA		-0.131 (-0.698, 0.437)	0.0%		
ZOL vs PLA		-0.060 (-0.525, 0.406)	0.0%		

CINeMA confidence rating

Comparison	Number of studies	Within-study bias	Reporting bias	Indirectness	Imprecision	Heterogeneity	Incoherence	Confidence rating

BLO vs FLU	1	Some concerns	Some concerns	No concerns	Major concerns	No concerns	No concerns	Low
BLO vs PLA	1	Some concerns	Some concerns	No concerns	Major concerns	No concerns	No concerns	Low
ESZ vs PLA	2	No concerns	Some concerns	No concerns	Major concerns	No concerns	No concerns	Low
FLU vs PLA	2	Some concerns	Some concerns	No concerns	Major concerns	No concerns	No concerns	Low
TEM vs PLA	1	No concerns	Some concerns	No concerns	Major concerns	No concerns	No concerns	Low
ZOL vs PLA	2	Some concerns	Some concerns	No concerns	Major concerns	No concerns	No concerns	Low
ZOP vs PLA	1	Some concerns	Some concerns	No concerns	Major concerns	No concerns	No concerns	Low
BLO vs ESZ	0	Some concerns	Some concerns	No concerns	Major concerns	No concerns	No concerns	Very low
BLO vs TEM	0	Some concerns	Some concerns	No concerns	Major concerns	No concerns	No concerns	Very low
BLO vs ZOL	0	Some concerns	Some concerns	No concerns	Major concerns	No concerns	No concerns	Very low
BLO vs ZOP	0	Some concerns	Some concerns	No concerns	Major concerns	No concerns	No concerns	Very low
ESZ vs FLU	0	Some concerns	Some concerns	No concerns	Major concerns	No concerns	No concerns	Very low
ESZ vs TEM	0	No concerns	Some concerns	No concerns	Major concerns	No concerns	No concerns	Very low
ESZ vs ZOL	0	Some concerns	Some concerns	No concerns	Major concerns	No concerns	No concerns	Very low
ESZ vs ZOP	0	Some concerns	Some concerns	No concerns	Major concerns	No concerns	No concerns	Very low
FLU vs TEM	0	Some concerns	Some concerns	No concerns	Major concerns	No concerns	No concerns	Very low
FLU vs ZOL	0	Some concerns	Some concerns	No concerns	Major concerns	No concerns	No concerns	Very low
FLU vs ZOP	0	Some concerns	Some concerns	No concerns	Major concerns	No concerns	No concerns	Very low
TEM vs ZOL	0	Some concerns	Some concerns	No concerns	Major concerns	No concerns	No concerns	Very low
TEM vs ZOP	0	Some concerns	Some concerns	No concerns	Major concerns	No concerns	No concerns	Very low
ZOL vs ZOP	0	Some concerns	Some concerns	No concerns	Major concerns	No concerns	No concerns	Very low

Sensitivity analysis excluding CPAP titration studies

League table (SMD with 95% confidence interval)

BLO	-0.065 (-1.072, 0.941)	0.065 (-0.683, 0.814)	-0.255 (-1.228, 0.718)	-0.006 (-0.887, 0.876)	0.078 (-0.959, 1.116)	-0.065 (-0.814, 0.683)
	ESZ	0.131 (-0.749, 1.010)	-0.190 (-1.105, 0.726)	0.060 (-0.758, 0.877)	0.144 (-0.840, 1.127)	0.000 (-0.672, 0.672)
		FLU	-0.320 (-1.162, 0.521)	-0.071 (-0.805, 0.663)	0.013 (-0.902, 0.928)	-0.131 (-0.698, 0.437)
			TEM	0.249 (-0.527, 1.026)	0.333 (-0.616, 1.283)	0.190 (-0.432, 0.811)
				ZOL	0.084 (-0.772, 0.940)	-0.060 (-0.525, 0.406)
					ZOP	-0.144 (-0.862, 0.575)
						PLA

Global heterogeneity

Between study variance (τ^2): 0.000 (heterogeneity assessment: low)

Random-effects design-by-treatment interaction model

χ^2 statistic: 0.206 (1 degrees of freedom), P value: 0.650

Local heterogeneity (I^2) and incoherence (SIDE test)

	NMA SMD	Direct SMD	I^2	Indirect SMD	P value (SIDE test)
BLO vs FLU	0.065(-0.683,0.814)	0.000(-0.800,0.800)		0.525(-1.598,2.648)	0.650
BLO vs PLA	-0.065(-0.814,0.683)	0.000(-0.800,0.800)		-0.525(-2.648,1.598)	0.650
FLU vs PLA		-0.131 (-0.698, 0.437)	0.0%		
ZOL vs PLA		-0.060 (-0.525, 0.406)	0.0%		

Subgroup analysis including studies involved only non-CPAP users

League table (SMD with 95% confidence interval)

ESZ	-0.014 (-0.934, 0.906)	0.144 (-0.840, 1.127)	0.000 (-0.672, 0.672)
	ZOL	0.158 (-0.796, 1.112)	0.014 (-0.614, 0.642)
		ZOP	-0.144 (-0.862, 0.575)
			PLA

Network meta-analysis by classifying medications into their drug classes

League table (SMD with 95% confidence interval)

BENZs	0.078 (-0.460, 0.616)	0.015 (-0.404, 0.434)
	Z drugs	-0.063 (-0.401, 0.275)
		PLA

Global heterogeneity

Between study variance (τ^2): 0.000 (heterogeneity assessment: low)

Random-effects design-by-treatment interaction model

na

Local heterogeneity (I^2) and incoherence (SIDE test)

	NMA SMD	Direct SMD	I^2	Indirect SMD	P value (SIDE test)
BENZs vs PLA		0.015 (-0.404, 0.434)	0.0%		
Z drugs vs PLA		-0.063 (-0.401, 0.275)	0.0%		

Appendix S11. Arousal index during total sleep time

7 studies, 330 participants

League table (SMD with 95% confidence interval)

ESZ	-0.578 (-1.283, 0.128)	-0.175 (-0.888, 0.539)	-0.454 (-0.956, 0.048)	**-0.420 (-0.754, -0.086)**	
	TEM	0.403 (-0.482, 1.288)	0.124 (-0.601, 0.849)	0.158 (-0.463, 0.779)	
		ZOL	-0.279 (-1.012, 0.454)	-0.245 (-0.876, 0.385)	
			ZOP	0.034 (-0.341, 0.408)	
				PLA	

SUCRA

ESZ	74.6%
TEM	16.5%
ZOL	69.8%
ZOP	29.1%
PLA	38.7%

Global heterogeneity

Between study variance (τ^2): 0.000 (heterogeneity assessment: low)

Random-effects design-by-treatment interaction model

na

Local heterogeneity (I^2) and incoherence (SIDE test)

	NMA SMD	Direct SMD	I^2	Indirect SMD	P value (SIDE test)
ESZ vs PLA		-0.420 (-0.754, -0.086)	0.0%		
ZOP vs PLA		0.034 (-0.341, 0.408)	0.0%		

CINeMA confidence rating

Comparison	Number of studies	Within-study bias	Reporting bias	Indirectness	Imprecision	Heterogeneity	Incoherence	Confidence rating
ESZ vs PLA	2	No concerns	Some concerns	No concerns	No concerns	Major concerns	Major concerns	Very low
TEM vs PLA	1	No concerns	Some concerns	No concerns	Major concerns	No concerns	Major concerns	Very low
ZOL vs PLA	1	Some concerns	Some concerns	No concerns	Major concerns	No concerns	Major concerns	Very low
ZOP vs PLA	3	Some concerns	Some concerns	No concerns	Major concerns	No concerns	Major concerns	Very low
ESZ vs TEM	0	No concerns	Some concerns	No concerns	Major concerns	No concerns	Major concerns	Very low
ESZ vs ZOL	0	Some concerns	Some concerns	No concerns	Major concerns	No concerns	Major concerns	Very low

ESZ vs ZOP	0	Some concerns	Some concerns	No concerns	Major concerns	No concerns	Major concerns	Very low
TEM vs ZOL	0	Some concerns	Some concerns	No concerns	Major concerns	No concerns	Major concerns	Very low
TEM vs ZOP	0	Some concerns	Some concerns	No concerns	Major concerns	No concerns	Major concerns	Very low
ZOL vs ZOP	0	Some concerns	Some concerns	No concerns	Major concerns	No concerns	Major concerns	Very low

Sensitivity analysis excluding CPAP titration studies

League table (SMD with 95% confidence interval)

ESZ	-0.359 (-1.222, 0.504)	0.044 (-0.827, 0.914)	-0.236 (-0.942, 0.471)	-0.202 (-0.801, 0.398)
	TEM	0.403 (-0.482, 1.288)	0.124 (-0.601, 0.849)	0.158 (-0.463, 0.779)
		ZOL	-0.279 (-1.012, 0.454)	-0.245 (-0.876, 0.385)
			ZOP	0.034 (-0.341, 0.408)
				PLA

Global heterogeneity

Between study variance (τ^2): 0.000 (heterogeneity assessment: low)

Random-effects design-by-treatment interaction model

na

Local heterogeneity (I^2) and incoherence (SIDE test)

	NMA SMD	Direct SMD	I^2	Indirect SMD	P value (SIDE test)
ESZ vs PLA		-0.202 (-0.801, 0.398)	0.0%		

Subgroup analysis including studies involved only non-CPAP users

League table (SMD with 95% confidence interval)

ESZ	0.044 (-0.827, 0.914)	-0.236 (-0.942, 0.471)	-0.202 (-0.801, 0.398)
	ZOL	-0.279 (-1.012, 0.454)	-0.245 (-0.876, 0.385)
		ZOP	0.034 (-0.341, 0.408)

			PLA

Network meta-analysis by classifying medications into their drug classes

League table (SMD with 95% confidence interval)

BENZs	0.233 (-0.450, 0.916)	0.158 (-0.463, 0.779)
	Z drugs	-0.075 (-0.359, 0.208)
		PLA

Global heterogeneity

Between study variance (τ^2): 0.000 (heterogeneity assessment: low)

Random-effects design-by-treatment interaction model

na

Local heterogeneity (I^2) and incoherence (SIDE test)

	NMA SMD	Direct SMD	I^2	Indirect SMD	P value (SIDE test)
Z drugs vs PLA		-0.075 (-0.359, 0.208)	0.0%		

Appendix S12. All-cause discontinuation

17 studies, 1313 participants

League table (OR with 95% confidence interval) **SUCRA**

DAR	0.296 (0.012, 7.624)	0.481 (0.004, 52.615)	0.689 (0.036, 13.057)	0.148 (0.007, 3.341)	0.481 (0.004, 51.951)	0.481 (0.004, 52.615)	0.575 (0.006, 59.603)	0.320 (0.019, 5.405)	0.334 (0.017, 6.711)	0.481 (0.041, 5.636)		DAR	75.4%
	ESZ	1.625 (0.018, 149.980)	2.326 (0.162, 33.378)	0.500 (0.029, 8.688)	1.625 (0.018, 148.013)	1.625 (0.018, 149.980)	1.942 (0.022, 169.542)	1.081 (0.086, 13.643)	1.128 (0.074, 17.257)	1.625 (0.195, 13.539)		ESZ	19.0%
		FLU	1.431 (0.019, 106.612)	0.308 (0.004, 25.860)	1.000 (0.004, 282.280)	1.000 (0.004, 285.260)	1.195 (0.004, 326.131)	0.665 (0.010, 45.828)	0.694 (0.009, 53.801)	1.000 (0.018, 54.467)		FLU	41.1%
			LEM	0.215 (0.018, 2.623)	0.699 (0.010, 51.326)	0.699 (0.009, 52.039)	0.835 (0.012, 58.668)	0.465 (0.107, 2.022)	0.485 (0.046, 5.111)	0.699 (0.139, 3.505)		LEM	71.3%
				RAM	3.250 (0.039, 269.509)	3.250 (0.039, 273.144)	3.884 (0.049, 308.432)	2.163 (0.203, 22.993)	2.256 (0.173, 29.462)	3.250 (0.480, 21.997)		RAM	12.1%
					TEM	1.000 (0.004, 282.280)	1.195 (0.004, 322.660)	0.665 (0.010, 45.187)	0.694 (0.009, 53.069)	1.000 (0.019, 53.662)		TEM	42.1%
						TRI	1.195 (0.004, 326.131)	0.665 (0.010, 45.828)	0.694 (0.009, 53.801)	1.000 (0.018, 54.467)		TRI	43.1%
							zal	0.557 (0.009, 36.133)	0.581 (0.008, 42.487)	0.837 (0.016, 42.790)		ZAL	66.7%
								ZOL	1.043 (0.115, 9.493)	1.503 (0.374, 6.032)		ZOL	25.3%
									ZOP	1.441 (0.259, 8.017)		ZOP	30.6%
										PLA		PLA	44.8%

Global heterogeneity

Between study variance (τ^2): 0.000 (heterogeneity assessment: low)

Random-effects design-by-treatment interaction model

χ^2 statistic: 0.342 (2 degrees of freedom), P value: 0.843

Local heterogeneity (I^2) and incoherence (SIDE test)

	NMA OR	Direct OR	I^2	Indirect OR	P value (SIDE test)
LEM vs ZOL	0.465(0.107,2.022)	0.507(0.101,2.553)		0.307(0.009,10.542)	0.801
LEM vs PLA	0.699(0.139,3.505)	0.711(0.110,4.582)	0.0%	0.663(0.026,16.608)	0.970
ZOL vs PLA	1.503(0.374,6.032)	1.688(0.395,7.212)		0.428(0.004,50.973)	0.591

ESZ vs PLA		1.625 (0.195, 13.539)	0.0%		

CINeMA confidence rating

Comparison	Number of studies	Within-study bias	Reporting bias	Indirectness	Imprecision	Heterogeneity	Incoherence	Confidence rating
DAR vs PLA	1	Some concerns	Some concerns	No concerns	Major concerns	No concerns	No concerns	Low
ESZ vs PLA	3	Some concerns	Some concerns	No concerns	Major concerns	No concerns	No concerns	Low
FLU vs PLA	1	Some concerns	Some concerns	No concerns	Major concerns	No concerns	No concerns	Low
LEM vs ZOL	1	Some concerns	Some concerns	No concerns	Major concerns	No concerns	No concerns	Low
LEM vs PLA	2	Some concerns	Some concerns	No concerns	Major concerns	No concerns	No concerns	Low
RAM vs PLA	1	Some concerns	Some concerns	No concerns	Major concerns	No concerns	No concerns	Low
TEM vs PLA	1	Some concerns	Some concerns	No concerns	Major concerns	No concerns	No concerns	Low
TRI vs PLA	1	Some concerns	Some concerns	No concerns	Major concerns	No concerns	No concerns	Low
ZAL vs PLA	1	Some concerns	Some concerns	No concerns	Major concerns	No concerns	No concerns	Low
ZOL vs PLA	4	Some concerns	Some concerns	No concerns	Major concerns	No concerns	No concerns	Low
ZOP vs PLA	3	Some concerns	Some concerns	No concerns	Major concerns	No concerns	No concerns	Low
DAR vs ESZ	0	Some concerns	Some concerns	No concerns	Major concerns	No concerns	No concerns	Very low
DAR vs FLU	0	Some concerns	Some concerns	No concerns	Major concerns	No concerns	No concerns	Very low
DAR vs LEM	0	Some concerns	Some concerns	No concerns	Major concerns	No concerns	No concerns	Very low
DAR vs RAM	0	Some concerns	Some concerns	No concerns	Major concerns	No concerns	No concerns	Very low
DAR vs TEM	0	Some concerns	Some concerns	No concerns	Major concerns	No concerns	No concerns	Very low
DAR vs TRI	0	Some concerns	Some concerns	No concerns	Major concerns	No concerns	No concerns	Very low
DAR vs ZAL	0	Some concerns	Some concerns	No concerns	Major concerns	No concerns	No concerns	Very low
DAR vs ZOL	0	Some concerns	Some concerns	No concerns	Major concerns	No concerns	No concerns	Very low
DAR vs ZOP	0	Some concerns	Some concerns	No concerns	Major concerns	No concerns	No concerns	Very low
ESZ vs FLU	0	Some concerns	Some concerns	No concerns	Major concerns	No concerns	No concerns	Very low
ESZ vs LEM	0	Some concerns	Some concerns	No concerns	Major concerns	No concerns	No concerns	Very low
ESZ vs RAM	0	Some concerns	Some concerns	No concerns	Major concerns	No concerns	No concerns	Very low
ESZ vs TEM	0	Some concerns	Some concerns	No concerns	Major concerns	No concerns	No concerns	Very low
ESZ vs TRI	0	Some concerns	Some concerns	No concerns	Major concerns	No concerns	No concerns	Very low
ESZ vs ZAL	0	Some concerns	Some concerns	No concerns	Major concerns	No concerns	No concerns	Very low
ESZ vs ZOL	0	Some concerns	Some concerns	No concerns	Major concerns	No concerns	No concerns	Very low
ESZ vs ZOP	0	Some concerns	Some concerns	No concerns	Major concerns	No concerns	No concerns	Very low
FLU vs LEM	0	Some concerns	Some concerns	No concerns	Major concerns	No concerns	No concerns	Very low
FLU vs RAM	0	Some concerns	Some concerns	No concerns	Major concerns	No concerns	No concerns	Very low
FLU vs TEM	0	Some concerns	Some concerns	No concerns	Major concerns	No concerns	No concerns	Very low

FLU vs TRI	0	Some concerns	Some concerns	No concerns	Major concerns	No concerns	No concerns	Very low
FLU vs ZAL	0	Some concerns	Some concerns	No concerns	Major concerns	No concerns	No concerns	Very low
FLU vs ZOL	0	Some concerns	Some concerns	No concerns	Major concerns	No concerns	No concerns	Very low
FLU vs ZOP	0	Some concerns	Some concerns	No concerns	Major concerns	No concerns	No concerns	Very low
LEM vs RAM	0	Some concerns	Some concerns	No concerns	Major concerns	No concerns	No concerns	Very low
LEM vs TEM	0	Some concerns	Some concerns	No concerns	Major concerns	No concerns	No concerns	Very low
LEM vs TRI	0	Some concerns	Some concerns	No concerns	Major concerns	No concerns	No concerns	Very low
LEM vs ZAL	0	Some concerns	Some concerns	No concerns	Major concerns	No concerns	No concerns	Very low
LEM vs ZOP	0	Some concerns	Some concerns	No concerns	Major concerns	No concerns	No concerns	Very low
RAM vs TEM	0	Some concerns	Some concerns	No concerns	Major concerns	No concerns	No concerns	Very low
RAM vs TRI	0	Some concerns	Some concerns	No concerns	Major concerns	No concerns	No concerns	Very low
RAM vs ZAL	0	Some concerns	Some concerns	No concerns	Major concerns	No concerns	No concerns	Very low
RAM vs ZOL	0	Some concerns	Some concerns	No concerns	Major concerns	No concerns	No concerns	Very low
RAM vs ZOP	0	Some concerns	Some concerns	No concerns	Major concerns	No concerns	No concerns	Very low
TEM vs TRI	0	Some concerns	Some concerns	No concerns	Major concerns	No concerns	No concerns	Very low
TEM vs ZAL	0	Some concerns	Some concerns	No concerns	Major concerns	No concerns	No concerns	Very low
TEM vs ZOL	0	Some concerns	Some concerns	No concerns	Major concerns	No concerns	No concerns	Very low
TEM vs ZOP	0	Some concerns	Some concerns	No concerns	Major concerns	No concerns	No concerns	Very low
TRI vs ZAL	0	Some concerns	Some concerns	No concerns	Major concerns	No concerns	No concerns	Very low
TRI vs ZOL	0	Some concerns	Some concerns	No concerns	Major concerns	No concerns	No concerns	Very low
TRI vs ZOP	0	Some concerns	Some concerns	No concerns	Major concerns	No concerns	No concerns	Very low
ZAL vs ZOL	0	Some concerns	Some concerns	No concerns	Major concerns	No concerns	No concerns	Very low
ZAL vs ZOP	0	Some concerns	Some concerns	No concerns	Major concerns	No concerns	No concerns	Very low
ZOL vs ZOP	0	Some concerns	Some concerns	No concerns	Major concerns	No concerns	No concerns	Very low

Appendix S13. Adverse event-related discontinuation

18 studies, 1491 participants

League table (OR with 95% confidence interval) **SUCRA**

DAR	0.556 (0.007, 43.371)	1.000 (0.004, 276.663)	2.068 (0.027, 158.539)	0.806 (0.003, 221.938)	1.000 (0.004, 273.746)	1.000 (0.004, 276.663)	1.195 (0.005, 316.208)	0.837 (0.012, 60.576)	0.935 (0.007, 120.121)	1.000 (0.019, 52.154)		DAR	43.9%
	ESZ	1.798 (0.022, 145.853)	3.719 (0.288, 47.952)	1.450 (0.018, 116.828)	1.798 (0.022, 143.897)	1.798 (0.022, 145.853)	2.149 (0.028, 164.613)	1.505 (0.129, 17.577)	1.682 (0.058, 48.371)	1.798 (0.289, 11.193)		ESZ	26.1%
		FLU	2.068 (0.026, 164.943)	0.806 (0.003, 228.835)	1.000 (0.004, 282.280)	1.000 (0.004, 285.260)	1.195 (0.004, 326.131)	0.837 (0.011, 63.055)	0.935 (0.007, 124.449)	1.000 (0.018, 54.467)		FLU	46.5%
			LEM	0.390 (0.005, 30.880)	0.483 (0.006, 38.031)	0.483 (0.006, 38.552)	0.578 (0.008, 43.502)	0.405 (0.049, 3.367)	0.452 (0.016, 12.718)	0.483 (0.081, 2.887)		LEM	76.9%
				RAM	1.240 (0.004, 348.173)	1.240 (0.004, 351.848)	1.482 (0.005, 402.261)	1.038 (0.014, 77.649)	1.160 (0.009, 153.377)	1.240 (0.023, 67.034)		RAM	37.5%
					TEM	1.000 (0.004, 282.280)	1.195 (0.004, 322.660)	0.837 (0.011, 62.190)	0.935 (0.007, 122.940)	1.000 (0.019, 53.662)		TEM	42.5%
						TRI	1.195 (0.004, 326.131)	0.837 (0.011, 63.055)	0.935 (0.007, 124.449)	1.000 (0.018, 54.467)		TRI	47.0%
							ZAL	0.701 (0.010, 49.774)	0.783 (0.006, 98.919)	0.837 (0.016, 42.790)		ZAL	66.2%
								ZOL	1.117 (0.043, 29.137)	1.194 (0.231, 6.169)		ZOL	39.9%
									ZOP	1.069 (0.064, 17.902)		ZOP	42.4%
										PLA		PLA	63.8%

Global heterogeneity

Between study variance (τ^2): 0.000 (heterogeneity assessment: low)

Random-effects design-by-treatment interaction model

χ^2 statistic: 0.243 (2 degrees of freedom), P value: 0.886

Local heterogeneity (I^2) and incoherence (SIDE test)

	NMA OR	Direct OR	I^2	Indirect OR	P value (SIDE test)
LEM vs ZOL	0.405(0.049,3.367)	0.511(0.032,8.253)		0.293(0.011,7.703)	0.799
LEM vs PLA	0.483(0.081,2.887)	0.429(0.067,2.759)	0.0%	1.984(0.003,1195.357)	0.653
ZOL vs PLA	1.194(0.231,6.169)	1.180(0.218,6.397)	0.0%	1.459(0.001,1494.579)	0.954

ZOP vs PLA			0.0%		
ESZ vs PLA			0.0%		

CINeMA confidence rating

Comparison	Number of studies	Within-study bias	Reporting bias	Indirectness	Imprecision	Heterogeneity	Incoherence	Confidence rating
DAR vs PLA	1	Some concerns	Some concerns	No concerns	Major concerns	No concerns	No concerns	Low
ESZ vs PLA	4	No concerns	Some concerns	No concerns	Major concerns	No concerns	No concerns	Low
FLU vs PLA	1	Some concerns	Some concerns	No concerns	Major concerns	No concerns	No concerns	Low
LEM vs ZOL	1	Some concerns	Some concerns	No concerns	Major concerns	No concerns	No concerns	Low
LEM vs PLA	3	Some concerns	Some concerns	No concerns	Major concerns	No concerns	No concerns	Low
RAM vs PLA	1	Some concerns	Some concerns	No concerns	Major concerns	No concerns	No concerns	Low
TEM vs PLA	1	Some concerns	Some concerns	No concerns	Major concerns	No concerns	No concerns	Low
TRI vs PLA	1	Some concerns	Some concerns	No concerns	Major concerns	No concerns	No concerns	Low
ZAL vs PLA	1	Some concerns	Some concerns	No concerns	Major concerns	No concerns	No concerns	Low
ZOL vs PLA	4	Some concerns	Some concerns	No concerns	Major concerns	No concerns	No concerns	Low
ZOP vs PLA	2	Some concerns	Some concerns	No concerns	Major concerns	No concerns	No concerns	Low
DAR vs ESZ	0	Some concerns	Some concerns	No concerns	Major concerns	No concerns	No concerns	Very low
DAR vs FLU	0	Some concerns	Some concerns	No concerns	Major concerns	No concerns	No concerns	Very low
DAR vs LEM	0	Some concerns	Some concerns	No concerns	Major concerns	No concerns	No concerns	Very low
DAR vs RAM	0	Some concerns	Some concerns	No concerns	Major concerns	No concerns	No concerns	Very low
DAR vs TEM	0	Some concerns	Some concerns	No concerns	Major concerns	No concerns	No concerns	Very low
DAR vs TRI	0	Some concerns	Some concerns	No concerns	Major concerns	No concerns	No concerns	Very low
DAR vs ZAL	0	Some concerns	Some concerns	No concerns	Major concerns	No concerns	No concerns	Very low
DAR vs ZOL	0	Some concerns	Some concerns	No concerns	Major concerns	No concerns	No concerns	Very low
DAR vs ZOP	0	Some concerns	Some concerns	No concerns	Major concerns	No concerns	No concerns	Very low
ESZ vs FLU	0	Some concerns	Some concerns	No concerns	Major concerns	No concerns	No concerns	Very low
ESZ vs LEM	0	Some concerns	Some concerns	No concerns	Major concerns	No concerns	No concerns	Very low
ESZ vs RAM	0	Some concerns	Some concerns	No concerns	Major concerns	No concerns	No concerns	Very low
ESZ vs TEM	0	Some concerns	Some concerns	No concerns	Major concerns	No concerns	No concerns	Very low
ESZ vs TRI	0	Some concerns	Some concerns	No concerns	Major concerns	No concerns	No concerns	Very low
ESZ vs ZAL	0	Some concerns	Some concerns	No concerns	Major concerns	No concerns	No concerns	Very low
ESZ vs ZOL	0	Some concerns	Some concerns	No concerns	Major concerns	No concerns	No concerns	Very low
ESZ vs ZOP	0	Some concerns	Some concerns	No concerns	Major concerns	No concerns	No concerns	Very low
FLU vs LEM	0	Some concerns	Some concerns	No concerns	Major concerns	No concerns	No concerns	Very low

FLU vs RAM	0	Some concerns	Some concerns	No concerns	Major concerns	No concerns	No concerns	Very low
FLU vs TEM	0	Some concerns	Some concerns	No concerns	Major concerns	No concerns	No concerns	Very low
FLU vs TRI	0	Some concerns	Some concerns	No concerns	Major concerns	No concerns	No concerns	Very low
FLU vs ZAL	0	Some concerns	Some concerns	No concerns	Major concerns	No concerns	No concerns	Very low
FLU vs ZOL	0	Some concerns	Some concerns	No concerns	Major concerns	No concerns	No concerns	Very low
FLU vs ZOP	0	Some concerns	Some concerns	No concerns	Major concerns	No concerns	No concerns	Very low
LEM vs RAM	0	Some concerns	Some concerns	No concerns	Major concerns	No concerns	No concerns	Very low
LEM vs TEM	0	Some concerns	Some concerns	No concerns	Major concerns	No concerns	No concerns	Very low
LEM vs TRI	0	Some concerns	Some concerns	No concerns	Major concerns	No concerns	No concerns	Very low
LEM vs ZAL	0	Some concerns	Some concerns	No concerns	Major concerns	No concerns	No concerns	Very low
LEM vs ZOP	0	Some concerns	Some concerns	No concerns	Major concerns	No concerns	No concerns	Very low
RAM vs TEM	0	Some concerns	Some concerns	No concerns	Major concerns	No concerns	No concerns	Very low
RAM vs TRI	0	Some concerns	Some concerns	No concerns	Major concerns	No concerns	No concerns	Very low
RAM vs ZAL	0	Some concerns	Some concerns	No concerns	Major concerns	No concerns	No concerns	Very low
RAM vs ZOL	0	Some concerns	Some concerns	No concerns	Major concerns	No concerns	No concerns	Very low
RAM vs ZOP	0	Some concerns	Some concerns	No concerns	Major concerns	No concerns	No concerns	Very low
TEM vs TRI	0	Some concerns	Some concerns	No concerns	Major concerns	No concerns	No concerns	Very low
TEM vs ZAL	0	Some concerns	Some concerns	No concerns	Major concerns	No concerns	No concerns	Very low
TEM vs ZOL	0	Some concerns	Some concerns	No concerns	Major concerns	No concerns	No concerns	Very low
TEM vs ZOP	0	Some concerns	Some concerns	No concerns	Major concerns	No concerns	No concerns	Very low
TRI vs ZAL	0	Some concerns	Some concerns	No concerns	Major concerns	No concerns	No concerns	Very low
TRI vs ZOL	0	Some concerns	Some concerns	No concerns	Major concerns	No concerns	No concerns	Very low
TRI vs ZOP	0	Some concerns	Some concerns	No concerns	Major concerns	No concerns	No concerns	Very low
ZAL vs ZOL	0	Some concerns	Some concerns	No concerns	Major concerns	No concerns	No concerns	Very low
ZAL vs ZOP	0	Some concerns	Some concerns	No concerns	Major concerns	No concerns	No concerns	Very low
ZOL vs ZOP	0	Some concerns	Some concerns	No concerns	Major concerns	No concerns	No concerns	Very low

Appendix S14. At least one adverse event

11 studies, 999 participants

League table (OR with 95% confidence interval)

BLO	1.571 (0.029, 85.166)	1.000 (0.024, 42.073)	0.722 (0.017, 31.318)	1.957 (0.008, 461.739)	0.308 (0.005, 20.518)	1.000 (0.004, 235.804)	1.195 (0.005, 272.135)	0.684 (0.016, 29.809)	1.000 (0.024, 42.073)
	DAR	0.636 (0.027, 14.911)	0.459 (0.104, 2.018)	1.245 (0.018, 84.911)	0.196 (0.018, 2.094)	0.636 (0.009, 43.350)	0.761 (0.012, 49.511)	0.436 (0.098, 1.931)	0.636 (0.157, 2.579)
		FLU	0.722 (0.041, 12.697)	1.957 (0.015, 258.760)	0.308 (0.010, 9.338)	1.000 (0.008, 132.132)	1.195 (0.009, 151.851)	0.684 (0.039, 12.099)	1.000 (0.059, 16.890)
			LEM	2.712 (0.049, 150.010)	0.426 (0.059, 3.066)	1.386 (0.025, 76.585)	1.656 (0.031, 87.261)	0.949 (0.604, 1.490)	1.386 (0.854, 2.249)
				NIT	0.157 (0.002, 13.055)	0.511 (0.002, 142.879)	0.611 (0.002, 165.059)	0.350 (0.006, 19.412)	0.511 (0.010, 27.456)
					RAM	3.250 (0.039, 269.509)	3.884 (0.049, 308.432)	2.224 (0.307, 16.095)	3.250 (0.480, 21.997)
						TEM	1.195 (0.004, 322.660)	0.684 (0.012, 37.940)	1.000 (0.019, 53.662)
							ZAL	0.573 (0.011, 30.265)	0.837 (0.016, 42.790)
								ZOL	1.461 (0.877, 2.433)
									PLA

SUCRA

BLO	54.4%
DAR	70.5%
FLU	53.3%
LEM	46.1%
NIT	75.3%
RAM	28.4%
TEM	55.8%
ZAL	63.5%
ZOL	44.7%
PLA	58.0%

Global heterogeneity

Between study variance (τ^2): 0.000 (heterogeneity assessment: low)

Random-effects design-by-treatment interaction model

χ^2 statistic: 0.097 (1 degrees of freedom), P value: 0.756

Local heterogeneity (I^2) and incoherence (SIDE test)

	NMA OR	Direct OR	I^2	Indirect OR	P value (SIDE test)
BLO vs FLU	1.000(0.024,42.073)	1.000(0.018,54.467)		1.000(0.000,39202.665)	1.000
BLO vs PLA	1.000(0.024,42.073)	1.000(0.018,54.467)		1.000(0.000,39202.665)	1.000
LEM vs ZOL	0.949(0.604,1.490)	0.831(0.513,1.346)		2.387(0.668,8.528)	0.129
LEM vs PLA	1.386(0.854,2.249)	1.646(0.976,2.777)	0.0%	0.491(0.136,1.773)	0.087

ZOL vs PLA	1.461(0.877,2.433)	1.420(0.829,2.433)	64.6%	1.869(0.383,9.122)	0.748
FLU vs PLA		1.000 (0.059, 16.890)	0.0%		

CINeMA confidence rating

Comparison	Number of studies	Within-study bias	Reporting bias	Indirectness	Imprecision	Heterogeneity	Incoherence	Confidence rating
BLO vs FLU	1	Some concerns	Some concerns	No concerns	Major concerns	No concerns	No concerns	Low
BLO vs PLA	1	Some concerns	Some concerns	No concerns	Major concerns	No concerns	No concerns	Low
DAR vs PLA	1	Some concerns	Some concerns	No concerns	Major concerns	No concerns	No concerns	Low
FLU vs PLA	2	Some concerns	Some concerns	No concerns	Major concerns	No concerns	No concerns	Low
LEM vs ZOL	1	Some concerns	Some concerns	No concerns	Major concerns	No concerns	No concerns	Low
LEM vs PLA	3	Some concerns	Some concerns	No concerns	Major concerns	No concerns	No concerns	Low
NIT vs PLA	1	Some concerns	Some concerns	No concerns	Major concerns	No concerns	No concerns	Low
RAM vs PLA	1	Some concerns	Some concerns	No concerns	Major concerns	No concerns	No concerns	Low
TEM vs PLA	1	Some concerns	Some concerns	No concerns	Major concerns	No concerns	No concerns	Low
ZAL vs PLA	1	Some concerns	Some concerns	No concerns	Major concerns	No concerns	No concerns	Low
ZOL vs PLA	2	Some concerns	Some concerns	No concerns	Major concerns	No concerns	No concerns	Low
BLO vs DAR	0	Some concerns	Some concerns	No concerns	Major concerns	No concerns	No concerns	Very low
BLO vs LEM	0	Some concerns	Some concerns	No concerns	Major concerns	No concerns	No concerns	Very low
BLO vs NIT	0	Some concerns	Some concerns	No concerns	Major concerns	No concerns	No concerns	Very low
BLO vs RAM	0	Some concerns	Some concerns	No concerns	Major concerns	No concerns	No concerns	Very low
BLO vs TEM	0	Some concerns	Some concerns	No concerns	Major concerns	No concerns	No concerns	Very low
BLO vs ZAL	0	Some concerns	Some concerns	No concerns	Major concerns	No concerns	No concerns	Very low
BLO vs ZOL	0	Some concerns	Some concerns	No concerns	Major concerns	No concerns	No concerns	Very low
DAR vs FLU	0	Some concerns	Some concerns	No concerns	Major concerns	No concerns	No concerns	Very low
DAR vs LEM	0	Some concerns	Some concerns	No concerns	Major concerns	No concerns	No concerns	Very low
DAR vs NIT	0	Some concerns	Some concerns	No concerns	Major concerns	No concerns	No concerns	Very low
DAR vs RAM	0	Some concerns	Some concerns	No concerns	Major concerns	No concerns	No concerns	Very low
DAR vs TEM	0	Some concerns	Some concerns	No concerns	Major concerns	No concerns	No concerns	Very low
DAR vs ZAL	0	Some concerns	Some concerns	No concerns	Major concerns	No concerns	No concerns	Very low
DAR vs ZOL	0	Some concerns	Some concerns	No concerns	Major concerns	No concerns	No concerns	Very low
FLU vs LEM	0	Some concerns	Some concerns	No concerns	Major concerns	No concerns	No concerns	Very low
FLU vs NIT	0	Some concerns	Some concerns	No concerns	Major concerns	No concerns	No concerns	Very low
FLU vs RAM	0	Some concerns	Some concerns	No concerns	Major concerns	No concerns	No concerns	Very low
FLU vs TEM	0	Some concerns	Some concerns	No concerns	Major concerns	No concerns	No concerns	Very low

FLU vs ZAL	0	Some concerns	Some concerns	No concerns	Major concerns	No concerns	No concerns	Very low
FLU vs ZOL	0	Some concerns	Some concerns	No concerns	Major concerns	No concerns	No concerns	Very low
LEM vs NIT	0	Some concerns	Some concerns	No concerns	Major concerns	No concerns	No concerns	Very low
LEM vs RAM	0	Some concerns	Some concerns	No concerns	Major concerns	No concerns	No concerns	Very low
LEM vs TEM	0	Some concerns	Some concerns	No concerns	Major concerns	No concerns	No concerns	Very low
LEM vs ZAL	0	Some concerns	Some concerns	No concerns	Major concerns	No concerns	No concerns	Very low
NIT vs RAM	0	Some concerns	Some concerns	No concerns	Major concerns	No concerns	No concerns	Very low
NIT vs TEM	0	Some concerns	Some concerns	No concerns	Major concerns	No concerns	No concerns	Very low
NIT vs ZAL	0	Some concerns	Some concerns	No concerns	Major concerns	No concerns	No concerns	Very low
NIT vs ZOL	0	Some concerns	Some concerns	No concerns	Major concerns	No concerns	No concerns	Very low
RAM vs TEM	0	Some concerns	Some concerns	No concerns	Major concerns	No concerns	No concerns	Very low
RAM vs ZAL	0	Some concerns	Some concerns	No concerns	Major concerns	No concerns	No concerns	Very low
RAM vs ZOL	0	Some concerns	Some concerns	No concerns	Major concerns	No concerns	No concerns	Very low
TEM vs ZAL	0	Some concerns	Some concerns	No concerns	Major concerns	No concerns	No concerns	Very low
TEM vs ZOL	0	Some concerns	Some concerns	No concerns	Major concerns	No concerns	No concerns	Very low
ZAL vs ZOL	0	Some concerns	Some concerns	No concerns	Major concerns	No concerns	No concerns	Very low

Appendix S15. Headache

6 studies, 923 participants

League table (OR with 95% confidence interval)

ESZ	0.714 (0.209, 2.440)	0.954 (0.259, 3.515)	0.276 (0.029, 2.575)	0.622 (0.285, 1.360)
	LEM	1.337 (0.497, 3.595)	0.386 (0.039, 3.844)	0.872 (0.338, 2.251)
		ZOL	0.289 (0.028, 2.996)	0.652 (0.230, 1.852)
			ZOP	2.258 (0.278, 18.318)
				PLA

SCURA

ESZ	73.7%
LEM	69.7%
ZOL	74.9%
ZOP	27.7%
PLA	53.9%

Global heterogeneity

Between study variance (τ^2): 0.000 (heterogeneity assessment: low)

Random-effects design-by-treatment interaction model

χ^2 statistic: 0.286 (1 degrees of freedom), P value: 0.593

Local heterogeneity (I^2) and incoherence (SIDE test)

	NMA OR	Direct OR	I^2	Indirect OR	P value (SIDE test)
LEM vs ZOL	1.337(0.497,3.595)	1.468(0.515,4.184)		0.613(0.030,12.559)	0.593
LEM vs PLA	0.872(0.338,2.251)	0.804(0.298,2.172)		1.997(0.083,47.947)	0.593
ESZ vs PLA			0.0%		
ZOL vs PLA			0.0%		
ZOP vs PLA			0.0%		

CINeMA confidence rating

Comparison	Number of studies	Within-study bias	Reporting bias	Indirectness	Imprecision	Heterogeneity	Incoherence	Confidence rating
ESZ vs PLA	2	No concerns	Some concerns	No concerns	Major concerns	No concerns	No concerns	Low
LEM vs ZOL	1	Some concerns	Some concerns	No concerns	Major concerns	No concerns	No concerns	Low
LEM vs PLA	1	Some concerns	Some concerns	No concerns	Major concerns	No concerns	No concerns	Low

ZOL vs PLA	2	Some concerns	Some concerns	No concerns	Major concerns	No concerns	No concerns	Low
ZOP vs PLA	2	Some concerns	Some concerns	No concerns	Major concerns	No concerns	No concerns	Low
ESZ vs LEM	0	Some concerns	Some concerns	No concerns	Major concerns	No concerns	No concerns	Very low
ESZ vs ZOL	0	Some concerns	Some concerns	No concerns	Major concerns	No concerns	No concerns	Very low
ESZ vs ZOP	0	Some concerns	Some concerns	No concerns	Major concerns	No concerns	No concerns	Very low
LEM vs ZOP	0	Some concerns	Some concerns	No concerns	Major concerns	No concerns	No concerns	Very low
ZOL vs ZOP	0	Some concerns	Some concerns	No concerns	Major concerns	No concerns	No concerns	Very low

Appendix S16. Somnolence

5 studies, 656 participants

League table (OR with 95% confidence interval)

LEM	2.519 (0.393, 16.163)	3.816 (0.556, 26.201)	3.816 (0.902, 16.153)	
	ZOL	1.515 (0.145, 15.844)	1.515 (0.211, 10.862)	
		ZOP	1.000 (0.279, 3.584)	
			PLA	

SUCRA

LEM	22.8%
ZOL	58.7%
ZOP	75.2%
PLA	83.2%

Global heterogeneity

Between study variance (τ^2): 0.000 (heterogeneity assessment: low)

Random-effects design-by-treatment interaction model

χ^2 statistic: 0.645 (2 degrees of freedom), P value: 0.724

Local heterogeneity (I^2) and incoherence (SIDE test)

	NMA OR	Direct OR	I^2	Indirect OR	P value (SIDE test)
LEM vs ZOL	2.519(0.393,16.163)	3.140(0.373,26.406)		1.245(0.028,56.182)	0.678
LEM vs PLA	3.816(0.902,16.153)	3.331(0.727,15.264)	0.0%	12.718(0.137,1177.796)	0.583
ZOL vs PLA	1.515(0.211,10.862)	1.297(0.156,10.755)	0.0%	4.175(0.019,924.359)	0.693

CINeMA confidence rating

Comparison	Number of studies	Within-study bias	Reporting bias	Indirectness	Imprecision	Heterogeneity	Incoherence	Confidence rating
LEM vs ZOL	1	Some concerns	Some concerns	No concerns	Major concerns	No concerns	No concerns	Low
LEM vs PLA	3	Some concerns	Some concerns	No concerns	Major concerns	No concerns	No concerns	Low
ZOL vs PLA	2	Some concerns	Some concerns	No concerns	Major concerns	No concerns	No concerns	Low
ZOP vs PLA	1	Some concerns	Some concerns	No concerns	Major concerns	No concerns	No concerns	Low
LEM vs ZOP	0	Some concerns	Some concerns	No concerns	Major concerns	No concerns	No concerns	Very low
ZOL vs ZOP	0	Some concerns	Some concerns	No concerns	Major concerns	No concerns	No concerns	Very low

Appendix S17. CPAP use per night on all nights

3 studies, 282 participants

League table (SMD with 95% confidence interval)

ESZ	2.426 (-4.115, 8.968)	2.541 (-1.235, 6.316)	
	ZOL	0.114 (-5.228, 5.457)	
		PLA	

SCURA

ESZ	81.2%
ZOL	34.1%
PLA	40.1%

Global heterogeneity

Between study variance (τ^2): 7.436 (heterogeneity assessment: high)

Random-effects design-by-treatment interaction model

na

Local heterogeneity (I^2) and incoherence (SIDE test)

	NMA OR	Direct OR	I^2	Indirect OR	P value (SIDE test)
ESZ vs ZOL		2.541 (-1.235, 6.316)	99,0%		

CINeMA confidence rating

Comparison	Number of studies	Within-study bias	Reporting bias	Indirectness	Imprecision	Heterogeneity	Incoherence	Confidence rating
ESZ vs PLA	2	No concerns	Some concerns	No concerns	Major concerns	Major concerns	Major concerns	Very low
ZOL vs PLA	1	Major concerns	Some concerns	No concerns	Major concerns	No concerns	Major concerns	Very low
ESZ vs ZOL	0	Some concerns	Some concerns	No concerns	Major concerns	No concerns	Major concerns	Very low
